# Interspecific Mating Is Trivial and Asymmetrical Between Two Destructive *Anoplophora* Beetles

**DOI:** 10.3390/insects16040352

**Published:** 2025-03-27

**Authors:** Tian Xu, Wenbo Wang, Xiaoyuan Chen, Jing Ma, Ruixu Chen, Xue Sun, Yang Yang, Guohao Li, Yadi Deng, Dejun Hao

**Affiliations:** 1 Co-Innovation Center for the Sustainable Forestry in Southern China, Nanjing Forestry University, Nanjing 210037, China; wbwang@njfu.edu.cn (W.W.); cxy20429@163.com (X.C.); jingma1229@163.com (J.M.); sunxue24@mails.ucas.ac.cn (X.S.); yyang@njfu.edu.cn (Y.Y.); njfulgh@126.com (G.L.); dengyadi1@gmail.com (Y.D.); 2 School of Landscape Architecture, Jiangsu Vocational College of Agriculture and Forestry, Zhenjiang 212499, China; chenruixu@njfu.edu.cn

**Keywords:** *Anoplophora glabripennis*, *Anoplophora chinensis*, wood-boring pests, invasive species, interspecific mating, pheromone

## Abstract

The Asian longhorn beetle (ALB), *Anoplophora glabripennis*, and citrus longhorn beetle (CLB), *Anoplophora chinensis*, are two destructive invasive wood-boring pests, with high similarities in morphology, geographical distribution, host range, life cycle, adult behaviors and male-produced pheromone, implying a potential existence of interspecific interactions. Matings have been found to occur across females and males of the two species when manually paired in confined spaces. However, interspecific mating and its regulating factors are unclear between sympatric populations on hosts. Herein, by observing mountings and tracking the beetles that freely coexisted on host branches in cages, we found that the majority of mountings appeared within species; however, interspecific mountings occasionally occurred between male CLBs and female ALBs. The CLB was more active than the ALB at night. It seems that males actively searched for female ALBs, while the inverse was the case with CLBs. The main release periods of shared pheromone components overlapped between the two species, while compound ratios had significant differences. Our results unveil a trivial and asymmetrical interspecific mating between ALBs and CLBs, implying a risk of co-outbreaks of the two species in either native or invaded areas.

## Simple Summary

The Asian longhorn beetle (ALB), *Anoplophora glabripennis*, and citrus longhorn beetle (CLB), *Anoplophora chinensis*, are two destructive invasive wood-boring pests, with high similarities in morphology, geographical distribution, host range, life cycle, adult behaviors and male-produced pheromone components, implying a potential existence of interspecific interactions. Matings have been found to occur across females and males of the two species when manually paired in confined spaces. However, interspecific mating and its regulating factors are unclear between sympatric populations on hosts. In the present study, by observing mountings and tracking the beetles that freely coexisted on host branches in cages, we found that the majority of mountings appeared within species, but interspecific mountings occasionally occurred, mainly between male CLBs and female ALBs. The CLB showed higher activities than the ALB at night, and the two species likely had distinct mate-searching strategies. Moreover, we found that the main release periods of shared pheromone components overlapped between species, while compound ratios had significant differences. Our results unveil a trivial and asymmetrical interspecific mating in the sympatric populations of the ALB and the CLB. The differences in adult behaviors and the pheromone compound ratio can provide valuable references for improving pest control techniques.

## 1. Introduction

Longhorn beetles (Order: Coleoptera, Family: Cerambycidae) are a group of xylophagous insects, with the larvae primarily feeding on the tissues of bark, cambium and wood of multiple plant parts [1]. The boring of cerambycid larvae can cause irretrievable damage to host plants, particularly those that are live and healthy with high economic and ecological values [2]. However, the hidden living style in cerambycid larvae makes early detection of the infestation difficult, resulting in outbreaks and the global spread of some species [3,4].

The Asian longhorn beetle (ALB), *Anoplophora glabripennis*, and citrus longhorn beetle (CLB), *Anoplophora chinensis*, are such cerambycid species (subfamily: Lamiinae), which have both caused severe damages in native ranges and invaded various countries of multiple continents through international transportation of solid wood-packing materials or nursery stocks [5,6,7,8]. Both ALBs and CLBs are native to East Asia, mainly China and the Korean peninsula [9]. Outside native ranges, the ALB has been found or intercepted in at least 15 countries, with known populations still present in the United States, France, Italy, Switzerland, Japan and Lebanon [10]; the CLB has been found in at least 10 countries, with known populations still existing in Croatia, Italy and Turkey [11]. The white-spotted longhorn beetle, *Anoplophora malasiaca*, distributed throughout Japan, is usually considered a synonymy of CLB [9]. However, a recent study analyzing the phylogeny of multiple *Anoplophora* species using a PCR-based target enrichment museomics approach suggests that *A. malasiaca* is likely a distinct species from CLB [12].

**Figure 1:**
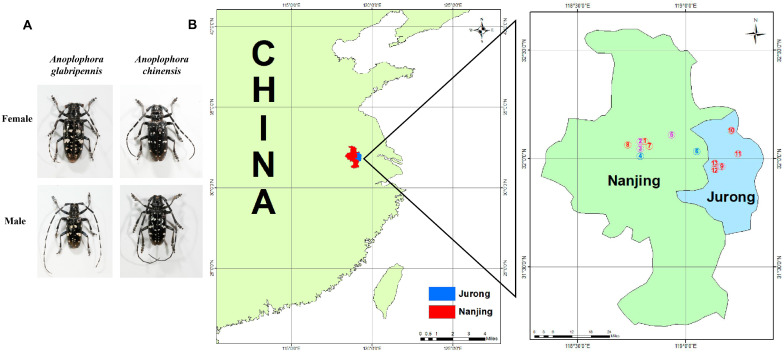
Morphology of female and male *Anoplophora glabripennis* (ALB) and *Anoplophora chinensis* (CLB) adults (photo credit: Yadi Deng) (**A**) and sites for beetle collection in 2023 (**B**). The numbers in (**B**) correspond to those in Table 1. Red numbers (1, 7–13) represent the sites where only CLB adults were collected; blue numbers (4 and 6) represent the sites where only ALB adults were collected; Purple numbers (2, 3 and 5) represent the sites where both species were captured.

As congeners, ALBs, and CLBs share a high similarity in adult external morphology (Figure 1A), with the presence of small projections or tubercles on the basal part of the elytra in CLBs the major distinction [8]. In China, they also have broadly overlapped geographic distribution and host ranges. Although the notorious damage caused by ALBs was mainly reported in northern China [6], its distribution records cover all provinces, autonomous regions, and municipalities, with the exception of Taiwan, Hainan, Hong Kong, and Macao [13]. The CLB is a serious pest in orchards, shelterbelts, and street trees in southern China [14], but its distribution records indicate its presence in the provinces and municipalities in North China [15]. Both species have over 100 reported host species, with many in the genera *Populus*, *Acer* and *Salix* as common preferred hosts [14,16]. The co-occurrence of ALBs and CLBs has been reported in some cities or even at the same sites in Shanghai, Zhejiang, Jiangsu and Anhui in eastern China [17,18,19,20]. Additionally, the life cycles of the ALB and CLB are similar, with June to July as the main overlapped adult period [8]. After eclosion, the adults both feed on young twigs [8], and males release 4-(*n*-heptyloxy)butan-1-ol and 4-(*n*-heptyloxy)butanal as the aggregation pheromone for reproduction [21,22].

Given the high similarities in geographical distribution, host range, life cycle, adult behaviors and pheromone components, whether there are interactions such as reproductive interference between CLBs and ALBs has been studied in recent years. In the United States, by using the individuals from the populations (the CLB was possibly a mixture of *A. chinensis* and *A. malasiaca*) that had been artificially reared for generations, Wang and Keena found that female ALBs did not mate with male CLBs, whereas matings occurred between female CLBs and male ALBs, which, however, did not produce any hatched eggs. The female ALB and male *A. malasiaca* mated and produced hatched eggs, while the female *A. malasiaca* and male ALB did not mate [23]. In contrast, in China, by placing three pairs of female and male ALB and CLB adults collected in the field together in a small box (28 cm × 21 cm × 17 cm), Qin et al. found that interspecific mating occurred across females and males of both species, with occurrence rates between male ALBs and female CLBs and between male CLBs and female ALBs at 44.6% and 30.2%, respectively. After interspecific matings, none of the laid eggs hatched [20]. In Japan, Sunamura et al. reported that, when placed in a container (22 cm × 30 cm × 6 cm) in pairs, 40% of the male *A. malasiaca* copulated with female ALBs, but male ALBs never mounted the female *A. malasiaca* [24]. The reason is still unclear [25]. Although some are contradictory, these results indicate that reproductive isolation probably exists between CLBs and ALBs, but interspecific mating could occur when they have an encounter.

It is noteworthy that the above studies were all conducted in very confined spaces, where the two species were manually placed together and could, thus, easily encounter each other. Whether interspecific mating occurs and how frequent it is among the sympatric populations of ALBs and CLBs under more natural conditions, such as when both freely living on a host, remain unknown. In insects, related species with high overlaps in living habitat, reproductive season, and pheromone components can exploit the differentiation in the diel rhythms of behavioral activities, in particular mating and pheromone release, and the ratios of pheromone components to minimize interspecific mating [26,27,28,29]. However, these are all unclear in ALBs and CLBs.

To control these pests and prevent their further spread, extensive studies have been carried out for developing effective pest management strategies for decades [5,6], including pheromone-based trapping techniques since a number of putative pheromone components have been identified in ALBs and CLBs [21,22,30,31,32,33,34]. Nevertheless, the trap catch numbers were low using these compounds in the field, which is insufficient for practical application [21,35]. To improve the control techniques, investigations are needed for a more comprehensive understanding of the behaviors, chemical communication systems, as well as the potential interactions between these destructive invasive wood-boring pests. Therefore, in the present study, we carried out experiments to investigate (1) the diel rhythms of mating and movement activities and the occurrence of interspecific mating in the ALB and CLB adults that freely coexisted on host branches in relatively larger spaces, and (2) the diel rhythms of releasing amounts and ratios of the shared aggregation-sex pheromone components in the males of both species.

## 2. Materials and Methods

### 2.1. Sources of Insects and Plants

ALB and CLB adults were hand collected on multiple tree species in Nanjing and Jurong, Jiangsu Province, China, from May to July 2023. The detailed site and host information is shown in Figure 1B and Table 1. All captured beetles were individually kept in 500 mL plastic cups in the laboratory of Nanjing Forestry University (NJFU) (approximately 25 °C, 40–50% RH, 14 h light and 10 h darkness). Three to four *Acer negundo* twigs (<10 mm diam, about 8 cm long) were supplied as food for each beetle, and refreshed every 2–3 days. Host branches were obtained at a nursery of Jiangsu Agricultural Expo Park, Jurong, Jiangsu Province, China (119°14′26.52″ E, 32°01′15.6″ N). The branches were cut into twigs and stored in a refrigerator at 4 °C for rearing beetles, or immediately used for the experiment.

### 2.2. Mounting and Movement Activity of Anoplophora Adults in a Cage

A nylon cage (1 m × 1 m × 1 m) was divided into 4 regions (0.5 m × 0.5 m × 1 m each). A 500 mL glass flask filled with fresh water was placed in the center of each region. Two *A. negundo* branches (~1 m long, <20 mm diam.) with leaves were held in each flask, with the cutting ends submerged in water. The top of the cage was covered with a black sunshade net.

**Figure 2:**
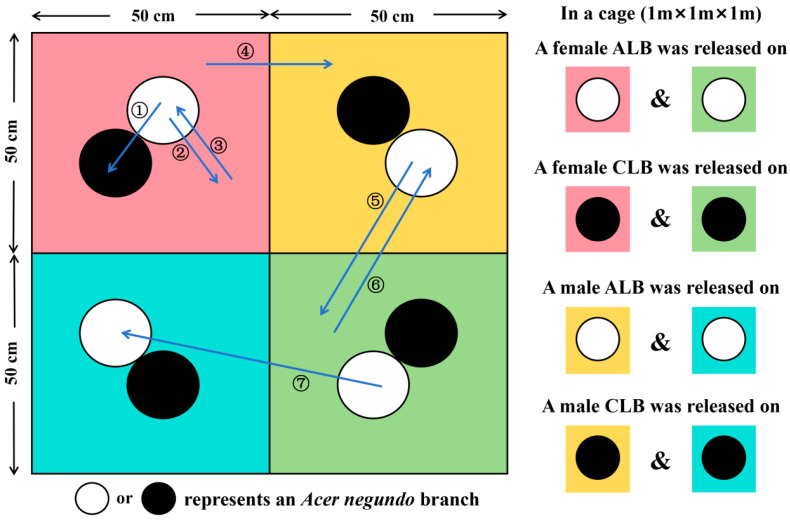
The schematic plan view of a cage used for observing the mountings and movements of *Anoplophora glabripennis* (ALB) and *Anoplophora chinensis* (CLB). The cage was divided into 4 regions (red, yellow, blue and green). The white or black circles represent a host branch (*Acer negundo*) placed in each region. At the beginning of the experiment, two female ALBs were individually released on the branches (white) in red and green regions; two female CLBs were individually released on the branches (black) in red and green regions; two male ALBs were individually released on the branches (white) in yellow and blue regions; two male CLBs were individually released on the branches (black) in yellow and blue regions. The arrows in various lengths and the numbers beside are representative examples for different types of position shifts listed in Table 2.

Two pairs of female and male ALB adults and two pairs of female and male CLB adults were marked with a spot (~0.5 cm diam.) of different acrylic colors on the middle part of an elytron. The basal parts of elytra were not covered by acrylic colors; thus, the two species can be discriminated by the naked eye. Two females in each species were marked red and green, respectively, and two males in each species were marked yellow and blue, respectively. The beetles with the same color mark were individually released on the branches in a region, and the regions were also labeled by attaching a zip tie (2.8 mm × 200 mm, in the same color with the beetles released) to the flasks. Within a region, the branches with ALBs and CLBs released were marked with white and black acrylic colors on bark, correspondingly. Thus, a cage was divided into multiple discernible parts (shown in Figure 2), which, together with the labeling of beetles, were used to track the beetles’ position shifts (between branches and regions) during the experiment.

The experiments were carried out twice on 23 July and 1 August 2023 on vacant land next to the Building of Forestry on the campus of NJFU (118°48′33″ E, 32°04′43″ N). The tree species surrounding the experimental area included *Prunus serrulata*, *Magnolia denudata*, *Acer buergerianum*, *Cedrus deodara*, *Acer coriaceifolium*, *Acer cordatum*, *Cercis chinensis*, *Osmanthus fragrans*, *Sambucus nigra*, and *Metasequoia glyptostroboides*. Five cages with 10 pairs of ALBs and CLBs and six cages with 12 pairs of ALBs and CLBs were set up on 23 July and 1 August. The beetles were released in the cages at 12 p.m. and the experiment lasted for 48 h. Every 2 h, the status (mounting or not) and position of each beetle were checked and recorded without direct touch. Copulation data were partially collected and not presented because copulations were difficult to confirm in some cases, i.e., the view was blocked by leaves or twigs, particularly at night from a distance without disrupting the beetles.

The movement activities of beetles were numerically evaluated by scoring the position changes of each beetle between two adjacent observation points. The scores were standardized from 1 to 4 according to the distances of position shifts (Table 2). However, by using the labeling methods described above, the movements of beetles on a branch or on the cage within a region were not distinguishable from no position shift; thus, all were scored as zero.

### 2.3. Aggregation-Sex Pheromone Release in Male Anoplophora Adults

Male ALB (*n* = 16) and CLB (*n* = 16) adults were individually aerated in 1000 mL Erlenmeyer flasks which contained two *A. negundo* twigs (~5 mm diam, ~10 cm long) for beetle feeding. Volatile chemicals were trapped on 200 mg Porapak Q (80/100 mesh, Supelco, Bellefonte, PA, USA). Charcoal-filtered air was pumped through the flasks at a constant flow rate of 200 mL/min by an air pump (QC-1B, Beijing Kean Labor Protection New Technology Co., Beijing, China). The aeration began at noon (12 p.m.) and lasted for 32 h. Every 4 h, trapped chemicals were eluted with 500 μL dichloromethane (DCM) (HPLC grade) and collected in a 1.5 mL glass vial. The volatile collections were totally performed 8 times. To minimize the potential disruptions of beetle relocation and new environment to pheromone release, the first two collections were excluded from data analyses. One male ALB and one male CLB did not release detectable pheromone components during 24 h (see Tables S2 and S3), which were also excluded from data analyses. The aeration was carried out under outdoor conditions (29–32 °C, 45–65% RH, approximately 14 h light and 10 h darkness).

The collected volatiles were then analyzed by a gas chromatograph (Agilent 8890 GC) fitted with an HP-5 MS column (30 m × 0.32 mm i.d., 0.25 μm film thickness), interfaced to a mass spectrometer (5977B, GC/MSD; Agilent Technologies, Santa Clara, CA, USA). Injections (3 μL) were made in splitless mode with an injector temperature of 250 °C. The oven was programmed from 60 °C, ramped at a rate of 10 °C/min to 250 °C and held at 250 °C for 5 min. Helium was used as the carrier gas. The MS ion source temperature was 230 °C, and mass spectra were obtained using electron impact (EI, 70 eV). 4-(*n*-Heptyloxy)butan-1-ol and 4-(*n*-heptyloxy)butanal in the samples were identified by matching their retention times and mass spectra with those of authentic standards that were purchased from Nanjing Zhilin Biotechnology Co., Ltd. (Nanjing, China). Quantifications of pheromone components in the samples were performed by developing calibration curves using authentic standards.

### 2.4. Data Analyses

The differences in the scores for position shifts between the males and females of the two species (*n* = 22) at each time point were analyzed using a generalized linear model, with the cage as a covariate, followed by Bonferroni adjusted pairwise comparisons. The difference between the numbers of male and female ALBs and CLBs that were observed performing interspecific mounting was analyzed by using a Chi-square test.

By comparing the positions of paired beetles when mounting started with their positions at the previous time point, the potential orientations of beetle movements for mounting were inferred. If a discernible position shift was observed in the male but not in the female, it was counted as “male oriented to female” and vice versa; if both sexes changed their positions for mounting, it was counted as “oriented to each other”. The difference between the numbers categorized into three conditions occurred in the intraspecific mountings of CLBs and ALBs was analyzed by using a Chi-square test.

The differences in the amounts of 4-(*n*-heptyloxy)butan-1-ol (alcohol) and 4-(*n*-heptyloxy)butanal (aldehyde), and the ratios (zero values excluded) of alcohol versus aldehyde released by male ALBs or CLBs in different periods of a day were analyzed using a generalized linear model followed by Bonferroni adjusted pairwise comparisons. The interspecific differences of those in each period were analyzed using Mann–Whitney U tests.

All data analyses were performed using the SPSS 22.0 statistic package (SPSS, Armonk, NY, USA).

## 3. Results

### 3.1. Mounting and Movement Activity of Anoplophora Adults in a Cage

#### 3.1.1. Mounting

**Figure 3:**
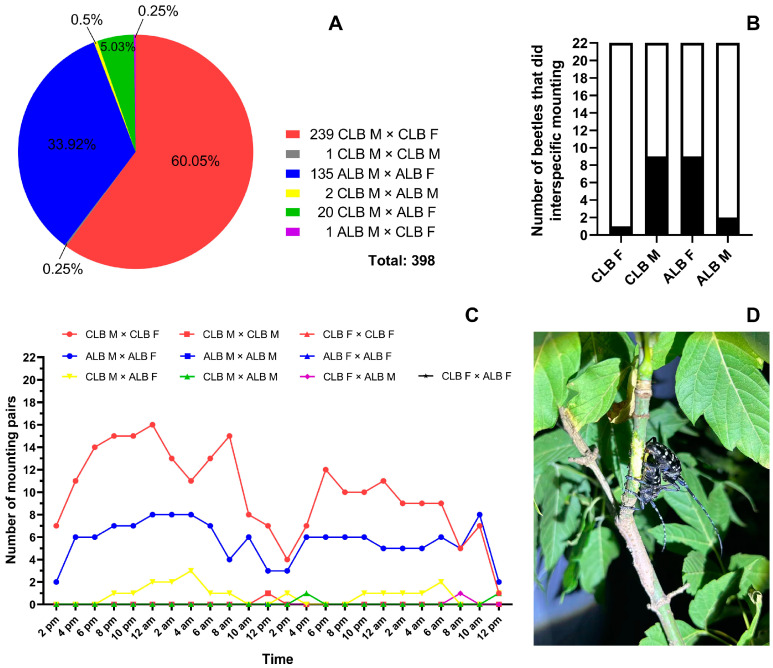
The observed mounting events among the male (M) and female (F) *Anoplophora glabripennis* (ALB) and *Anoplophora chinensis* (CLB) in the cages. (**A**) The proportions (in the pie chart) and numbers (at the lower right corner) of different types of mountings recorded in 24 observations (every 2 h) during 48 h. (**B**) The numbers of male and female CLB and ALB observed performing interspecific mounting during 48 h. (**C**) The numbers of different types of mountings observed at each time point during 48 h. (**D**) A copulation between a pair of beetles (photo credit: Xiaoyuan Chen and Jing Ma).

In the 24 observations during 48 h, a total of 398 mounting events (including possible repeated counts for the same pairs that lasted across time points) were observed. Most mountings were intraspecific (375/398, accounting for 94.22% of the total events), including 239 (60.05%) between male and female CLBs, 135 (33.92%) between male and female ALBs, and one between a pair of male CLBs. However, only 23 interspecific mounting events were observed (5.78% of the total events), most of which occurred between male CLBs and female ALBs (20/23). The mounting between the male ALB and female CLB was only observed once, and two events were observed between a male CLB and a male ALB. No mounting was observed between male ALBs or between the females of two species (Figure 3A). Among the tested beetles, nine of 22 male CLBs performed interspecific mountings, including eight paired with nine of 22 female ALBs and one paired with one male ALB. Only one of 22 female CLBs was mounted by a male ALB. Thus, the numbers of male and female ALBs and CLBs that did engage in interspecific mounting had statistically significant differences (Chi-square test: *df* = 3, *χ*^2^ = 14.2, *p* = 0.0026; Figure 3B).

The diel rhythms of intraspecific mountings were generally similar between the two species (Figure 3C). The number of mounting pairs in ALBs increased mainly between 12 p.m. and 4 p.m., followed by a relatively stable number until the next morning. However, the number of mounting pairs in CLBs kept increasing from 12 p.m. until midnight, then decreased. In both species, there was another peak at 8 a.m. or 10 a.m. Both species showed the lowest mounting numbers around noon (at 12 p.m. or 2 p.m.). Most of the interspecific mountings between male CLBs and female ALBs were observed between 8 p.m. and 8 a.m., with a peak in the early morning (at 4 a.m. or 6 a.m.).

The detailed mounting pairs observed in each cage at each time point can be found in Supporting Information.

#### 3.1.2. Movement

**Figure 4:**
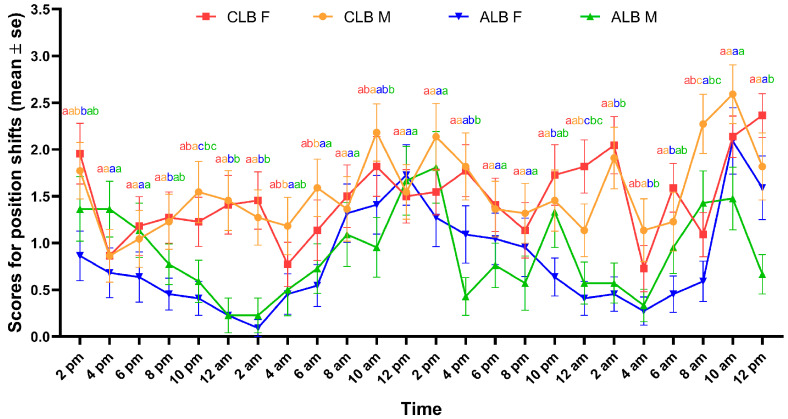
The scores for position shifts of male (M) and female (F) *Anoplophora glabripennis* (ALB) and *Anoplophora chinensis* (CLB) in the cages during 48 h. The colors of the lowercase letters above lines correspond to the colors of the lines that represent female (red) and male (orange) CLB, female (blue) and male (green) ALB. Different letters indicate statistically significant differences at the same time point (*p* < 0.05; Bonferroni adjusted pairwise comparisons following a generalized linear model). See detailed statistical results in Table S1.

Both CLB and ALB adults showed peaks for movement activity around noon (between 10 a.m. and 2 p.m.). However, the CLB was more active than the ALB at night, with one more movement activity peak appeared around midnight (between 10 p.m. and 2 a.m.), whereas the ALB had the least movement activity right after midnight (between 12 a.m. and 4 a.m.). Within species, no statistically significant difference in movement activity was detected between the sexes at almost all time points (Figure 4). The detailed statistical results are shown in Table S1.

**Figure 5:**
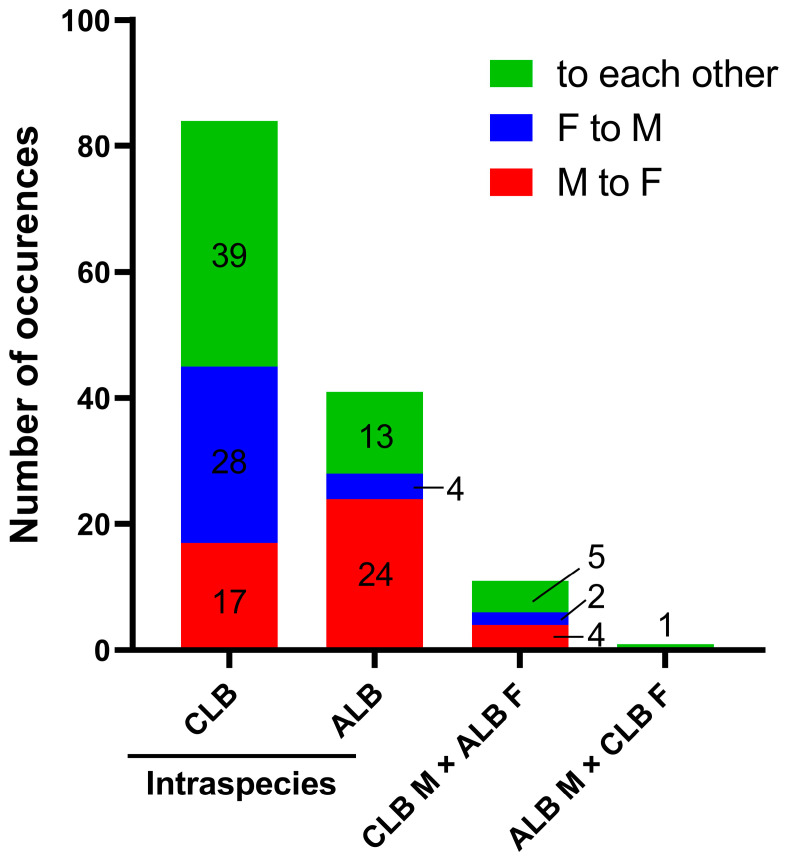
The numbers of three types of movement directions, including orientations of both sexes to each other (green), female to male (blue) and male to female (red), before intraspecific and interspecific mountings began among male (M) and female (F) *Anoplophora glabripennis* (ALB) and *Anoplophora chinensis* (CLB) in the cages. The orientations were inferred by comparing the positions of paired beetles when mounting started with their positions at the previous time point. The exact numbers for each type are shown in or by the bars.

The orientations of beetles potentially for mountings were inferred in 84, 41, 11 and one mounting events between male and female CLBs, male and female ALBs, male CLBs and female ALBs, and male ALBs and female CLBs, respectively. In the intraspecific mountings of CLBs, the orientations of both sexes to each other were the major type (39/84), followed by those of females to males (28/84) and males to females (17/84). However, males likely oriented to females before 24 of 41 ALB intraspecific mountings began, and the orientations of both sexes to each other and females to males were inferred in 13 and four mounting events, respectively. The potential orientations of sexes for intraspecific mountings had statistically significant differences between the two species (Chi-square test: *df* = 2, *χ*^2^ = 19.74, *p* < 0.001; Figure 5). In the interspecific mountings between male CLBs and female ALBs, five, four and two belonged to the orientations of both sexes to each other, males to females, and females to males, respectively. Both beetles had discernible position shifts for the mounting between a male ALB and a female CLB (Figure 5).

The detailed positions of each beetle recorded at each time point can be found in Supporting Information.

### 3.2. Aggregation-Sex Pheromone Release in Male Anoplophora Adults

**Figure 6:**
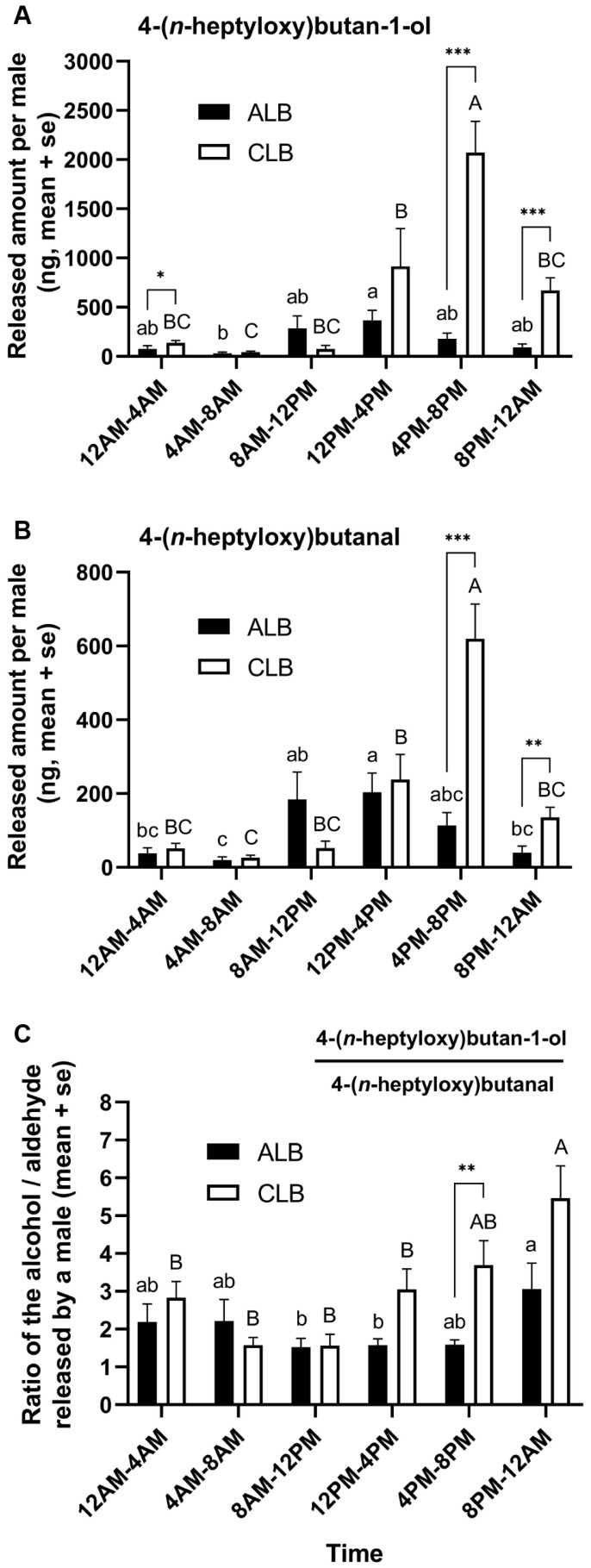
The amounts of male-produced aggregation-sex pheromone components, 4-(*n*-heptyloxy)butan-1-ol (**A**), 4-(*n*-heptyloxy)butanal (**B**), and the ratios of 4-(*n*-heptyloxy)butan-1-ol versus 4-(*n*-heptyloxy)butanal (**C**), released by male adults of *Anoplophora glabripennis* (ALB) and *Anoplophora chinensis* (CLB) in different periods of a day. The lowercase letters and uppercase letters indicate statistically significant differences among different time periods in male ALBs and CLBs, respectively (*p* < 0.05; Bonferroni adjusted pairwise comparisons following generalized linear model). The asterisk(s) indicate statistically significant differences between species in the same period (* 0.01 < *p* < 0.05, ** 0.001 ≤ *p* ≤ 0.01, *** *p* < 0.001; Mann–Whitney U test). The values for the amounts of 4-(*n*-heptyloxy)butan-1-ol and 4-(*n*-heptyloxy)butanal quantified in the volatiles released by male ALBs and CLBs in each period are shown in Tables S2 and S3.

Both male ALBs and CLBs showed significant variations in the releasing amounts of male-produced aggregation-sex pheromone components in different periods of a day (generalized linear mode: ALB, alcohol, *df* = 5, *χ*^2^ = 17.29, *p* = 0.004; aldehyde: *df* = 5, *χ*^2^ = 20.737, *p* = 0.001; CLB, alcohol, *df* = 5, *χ*^2^ = 73.816, *p* < 0.001; aldehyde: *df* = 5, *χ*^2^ = 111.058, *p* < 0.001; Figure 6A,B). In male ALBs, the major pheromone release period was from 8 a.m. to 8 p.m., with the peaks between 12 p.m. and 4 p.m. (alcohol: 365.36 ng, aldehyde: 203.99 ng, in average); the lowest amounts were found between 4 a.m. and 8 a.m. The main pheromone release period in male CLBs was from 12 p.m. to 12 a.m., with the peaks between 4 p.m. and 8 p.m. (alcohol: 2072.28 ng, aldehyde: 619.87 ng, on average); the lowest amounts were also found between 4 a.m. and 8 a.m. Between species, male CLBs released significantly more alcohol than male ALBs from 4 p.m. to 4 a.m. (Mann–Whitney U test: between 4 p.m. and 8 p.m., *p* < 0.001; between 8 p.m. and 12 a.m., *p* < 0.001; between 12 a.m. and 4 a.m., *p* = 0.016); male CLBs released significantly more aldehyde than male ALBs from 4 p.m. to 12 a.m. (Mann–Whitney U test: between 4 p.m. and 8 p.m., *p* < 0.001; between 8 p.m. and 12 a.m., *p* = 0.002).

In the two species, the ratios (alcohol versus aldehyde) of the released pheromone components also varied in different periods of a day (generalized linear mode: ALB, *df* = 5, *χ*^2^ = 14.125, *p* = 0.015; CLB, *df* = 5, *χ*^2^ = 34.945, *p* < 0.001; Figure 6C). The compound ratios in male ALBs ranged from 1.52 to 3.06, on average, with significant differences detected in the comparisons of 8 p.m. to 12 a.m. with the periods from 8 a.m. to 4 p.m. (Bonferroni adjusted pairwise comparisons: *p* < 0.05). The compound ratios in male CLB ranged from 1.56 to 5.46, on average, with significant differences found in the comparisons of 8 p.m. to 12 a.m. with the periods from 12 a.m. to 4 p.m. (Bonferroni adjusted pairwise comparisons: *p* < 0.05). Between species, the ratio in CLB (average 3.69) was significantly higher than that in ALB (average 1.59) between 4 p.m. and 8 p.m. (Mann–Whitney U test: *p* = 0.007), and the difference was marginally significant between 12 p.m. and 4 p.m. (Mann–Whitney U test: *p* = 0.067).

## 4. Discussion

The interspecific matings have been found across females and males of ALBs and CLBs after they encounter each other among the populations distributed in Bengbu, Anhui Province, and Cixi, Zhejiang Province, in eastern China [20]. The interspecific matings had lower occurrence rates compared with intraspecific matings, which may be attributed to a potential differentiation in the compositions of cuticular hydrocarbons (CHCs) between the two species [20,25]. In the present study, the results indicate that, when both sexes of ALB and CLB adults freely coexisted on host branches in cages, mountings occurred mainly within species; interspecific mountings occasionally occurred, mostly between male CLBs and female ALBs. These results were different from those observed in confined spaces, suggesting that, with the exception of the role of CHCs in reducing the occurrences of interspecific matings after encountering, there probably exist other factors lowering the encounter rate between species. Since our experiment was carried out in cages, the encounter rates of the beetles should be even lower in the field. Surprisingly, the interspecific mounting pattern found here is highly similar to that reported between ALB and *A. malasiaca* [23,24]. The beetles used in this study were collected from the sites in Nanjing and Jurong, Jiangsu Province that are also in eastern China. However, we currently cannot exclude a probability that the CLB used in our study was *A. malasiaca*, which needs further clarification.

The diel rhythms of intraspecific mountings in the two species showed a similar pattern, observed mainly from noon to the next morning. The mounting between a pair of beetles can last for several hours. However, a difference was found in the increasing periods of mounting numbers between the two species, which were primarily between 12 p.m. and 4 p.m. in ALB but from 12 p.m. to midnight in CLB, suggesting possible differentiation in the mate searching rhythms. Both species had an additional peak of mounting numbers in the morning, but the mountings did not last long.

CLB and ALB adults showed a significant difference in the diel rhythms of movement activity. ALBs were only active in the daytime, while CLBs were also active at night. Notably, most interspecific mountings between male CLB and female ALB occurred at night and in the early morning when CLB was more active than ALB. In longhorn beetles, mounting behavior is induced after the antenna of a male touches the body of a female by perceiving the CHCs on the body surface. The touch of the female antenna on the male body does not trigger mating behavior, and the encounters of the same sexes often lead to avoidance behavior or fighting [26,36]. Therefore, the interspecific mountings may primarily be induced in a part of male CLBs when they actively encounter female ALBs, but not when female CLBs encounter male ALBs at night. In the daytime, the interspecific mountings may be minimized with the beginning of the major pheromone release period in both species (discussed below) and the increased competition of male ALBs for mating when they become active [37,38].

Diel variations in pheromone release and behavioral responses to pheromones have been reported in cerambycids, which are considered to be able to minimize the cross-attraction between the species sharing the same pheromone components [39,40]. Our results show that males of both ALBs and CLBs can release the aggregation-sex pheromone in any period of the day, but in varying amounts and ratios. The major release period, in quantity, was from 8 a.m. to 8 p.m. in ALB and from 12 p.m. to 12 a.m. in CLB. Thus, the pheromone releases were not temporally staggered between the two species, with an overlap between 12 p.m. and 8 p.m. The major pheromone release periods coincided with the main periods of increasing mounting in both species, with the exception of a peak in CLBs that appeared in the morning. The matches in the time of pheromone release and mounting suggest a potential role of the male-produced pheromone in regulating mate locations, at least in a short distance [33]. The extra mounting peak in CLB may be due to the high movement activity of this species in the morning in space-confined cages. Moreover, the behavioral responses of pheromone receivers to constantly released pheromones can also have diel variations in longhorn beetles [39], which needs further investigation in ALBs and CLBs.

Using distinct ratios of pheromone components for intraspecific chemical communication is another common strategy for the sympatric insect species with the same pheromone components to avoid cross-attraction [28,29]. The ratios of two male-produced pheromone components in ALBs and CLBs both varied in different periods of the day, ranging from 1.52 to 3.06 in ALBs and from 1.56 to 5.46 in CLBs (alcohol versus aldehyde). During the main pheromone release periods, lower ratios (about 1.5) were found in ALBs, but, in contrast, the ratios became elevated in CLBs (greater than three). The significant difference in the ratios of the shared pheromone components may help the two species reduce the occurrence of interspecific mating. Meng et al. reported that the traps baited with a blend of synthetic male pheromone components at the ratio of 1:1 combined with host volatiles captured more female ALBs than those at the ratios of 1:8, 1:4, 4:1 and 8:1, and only the traps with the pheromone blend at 1:1 caught significantly more females than the control [41]. These results indicate that the behavioral responses of ALBs, at least females, to male-produced pheromones are probably ratio dependent, which may help them reduce the attraction of the pheromone released by CLBs. However, in CLBs, field trapping experiments showed that synthetic alcohol and aldehyde mixed at the ratio of 1:1 were significantly attractive to both sexes [21], suggesting that CLBs may be attracted to the pheromone released by ALBs [30]. Notably, there have not been any studies reporting the attractions of the pheromone blends at other ratios to CLBs. Thus, further investigations are strongly recommended to test whether the CLB-specific ratio can enhance the pheromone attraction to this beetle, which is not only important for clarifying the role of the pheromone compound ratio in mediating the chemical communication of the two species but also valuable for improving the semiochemical-based trapping technique used in pest management.

In the present study, tracking the positions of beetles afforded us an opportunity to estimate the potential orientations of male and female ALBs and CLBs for mating on hosts. By using our standards, different patterns were found between the two species in the movement directions before mountings began. In ALBs, the majority of mountings occurred after males had discernible movements toward females, suggesting that males may have played the main role in mate searching on hosts [37]. The searching behavior of males may be random [42,43] or regulated by the attractive compounds emitted by female adults [31,33,34]. Compared with ALBs, our results suggest that females are likely more active in mate searching on hosts in CLBs. To our knowledge, the volatile pheromones or attractants have been found only in the males of CLBs (not involving *A. malasiaca*) so far [21,30], which may mediate this mate-searching strategy. Similarly, the females of *A. malasiaca* have been reported to actively orient to males in a short distance [44]. Nevertheless, these preliminary results were generated from the analyses of beetle position shifts in relatively long-time intervals (2 h), which need to be confirmed by more precise observations. Whether the likely distinct mate-searching strategies between ALBs and CLBs can help reduce interspecific mating also deserves evaluation.

In summary, our results imply that interspecific mating is trivial and occurs asymmetrically between male CLBs and female ALBs when the two species freely coexist on host branches, which may be caused by complex factors, such as the differences in the diel rhythms of behavioral activities, the ratios of pheromone components, and mate-searching strategies. Considering the fact that both sexes of ALB and CLB adults can mate and females can oviposit repeatedly [37,45], we suppose that the occasional interspecific matings do not have significant impacts on the populations of the two species. Furthermore, ALBs typically initiate oviposition along the upper trunk and main branches, but CLBs usually lay eggs along the lower trunk and exposed roots, indicating a clear differentiation in the larval spatial niches between the two species [8]. Hence, co-outbreaks of these destructive pests are possible in either native ranges or invaded areas.

## Figures and Tables

**Table 1:** The site and host information for beetle collection in 2023.

City	#	Site	Longitude	Latitude	CLB	ALB	Host Species *
Nanjing	1	Campus of Nanjing Forestry University	118°48′28.44″E	32°04′44.76″ N	×		*Platanus* sp. *Lagerstroemia indica* *Melia azedarach*
	2	Xuanwuhu Park	118°47′34.44″ E	32°04′41.16″ N	×	×	*Salix babylonica*
	3	Zhenghe Park	118°47′20.04″ E	32°02′36.6″ N	×	×	*Salix babylonica* *Acer* sp.
	4	Bailuzhou Park	118°47′21.48″ E	32°0′38.52″ N		×	*Salix babylonica*
	5	Yangshan Park	118°56′0.6″ E	32°06′32.76″ N	×	×	*Salix babylonica*
	6	Tangshan outlets	119°02′57.48″ E	32°01′52.68″ N		×	*Salix babylonica* *Acer* sp.
	7	Nanjing Botanical Garden	118°49′53.76″ E	32°03′22.32″ N	×		*Acer negundo* *Lagerstroemia indica*
	8	Lijiang Road	118°43′52.32″ E	32°03′45″ N	×		*Platanus* sp.
Jurong	9	Campus of Jiangsu Vocational College of Agriculture and Forestry	119°09′55.44″ E	31°57′46.44″ N	×		*Lagerstroemia indica* *Salix babylonica*
	10	Xiashu Forestry Station	119°12′38.16″ E	32°07′39.36″ N	×		*Koelreuteria bipinnata* *Populus* sp.
	11	Jiangsu Agricultural Expo Park	119°14′26.52″ E	32°01′15.6″ N	×		*Acer negundo*
	12	Shishi Road	119°08′1.32″ E	31°57′13.68″ N	×		*Lagerstroemia indica* *Melia azedarach* *Koelreuteria bipinnata* *Acer* sp.
	13	Fudi Road	119°08′0.24″ E	31°58′14.52″ N	×		*Lagerstroemia indica*

* Host species indicate the trees on which the beetles were collected.

**Table 2:** The scoring standards for the positions shifts of beetles.

Score	Position Shift *
1	① from a branch to the other within a region ② from a branch to the cage within a region ③ from the cage to a branch within a region
2	④ between the cages of different regions
3	⑤ from a branch to the cage of other regions ⑥ from the cage to a branch in other regions
4	⑦ from a branch to a branch across regions

* ①–⑦ represent 7 kinds of discernible position shifts with different distances (also shown in Figure 2).

## Data Availability

All data supporting reported results in the present study can be found in the main text and Supporting materials.

## Supplementary Materials

The following supporting information can be downloaded at: https://www.mdpi.com/article/10.3390/insects16040352/s1. Table S1: Results from the statistical analyses of the differences between the scores for position shifts in female (F) and male (M) *Anoplophora glabripennis* (ALB) and *Anoplophora chinensis* (CLB) by using a generalized linear model followed by Bonferroni adjusted pairwise comparisons. Table S2: The amounts (ng) of male-produced aggregation-sex pheromone components, 4-(*n*-heptyloxy)butanal and 4-(*n*-heptyloxy)butan-1-ol, released by 16 male *Anoplophora glabripennis* adults in different periods of a day. Table S3: The amounts (ng) of male-produced aggregation-sex pheromone components, 4-(*n*-heptyloxy)butanal and 4-(*n*-heptyloxy)butan-1-ol, released by 16 male *Anoplophora chinensis* adults in different periods of a day. Figure S1: the positions and mounting pairs of beetles observed in the cages at the time points from 23 July to 25 July 2023. Figure S2: the positions and mounting pairs of beetles observed in the cages at the time points from 1 August to 3 August 2023.

## Abbreviations

The following abbreviations are used in this manuscript:

ALB	Asian longhorn beetle
CLB	Citrus longhorn beetle
