# Supplementary material for: Interspecific Mating Is Trivial and Asymmetrical Between Two Destructive Anoplophora Beetles
Source: Insects. 2025 Mar 27;16(4):352. doi: 10.3390/insects16040352 (PMC12027497; doi:10.3390/insects16040352)
Supplement: Supplementary file 1 [file insects-16-00352-s001.zip › insects-3546936-supplementary.pdf]

Supporting materials for

Interspecific Mating Is Trivial and Asymmetrical Between Two Destructive *Anoplophora* Beetles

Table S1. Results from the statistical analyses of the differences between the scores for position shifts in female (F) and male (M) *Anoplophora glabripennis* (ALB) and *Anoplophora chinensis* (CLB) by using Generalized linear model followed with Bonferroni adjusted pairwise comparisons

**Observation time points**	**Generalized linear model**				***P* values (Bonferroni adjusted pairwise comparisons)**					
	**Source of variation**	***d.f.***	χ^2	***P***	**CLBF *vs.* CLBM**	**CLBF *vs.* ALBF**	**CLBF *vs.* ALBM**	**CLBM *vs.* ALBF**	**CLBM *vs.* ALBM**	**ALBF *vs.* ALBM**
2 pm	Intercept	1	6.295	0.012	1	0.014	0.769	0.052	1	0.697
	Treatment	3	10.006	0.019						
	Covariate (cages)	1	0.477	0.49						
4 pm	Intercept	1	0.216	0.642	1	1	0.697	1	0.697	0.152
	Treatment	3	5.854	0.119						
	Covariate	1	0.942	0.332						
6 pm	Intercept	1	0.149	0.699	1	0.347	1	0.834	1	0.469
	Treatment	3	3.983	0.263						
	Covariate	1	0.092	0.762						
8 pm	Intercept	1	0.731	0.393	1	0.021	0.606	0.031	0.79	1
	Treatment	3	10.108	0.018						
	Covariate	1	0.078	0.78						
10 pm	Intercept	1	2.019	0.155	1	0.016	0.161	0.001	0.013	1
	Treatment	3	18.006	< 0.001						
	Covariate	1	0.582	0.445						
12 am	Intercept	1	9.899	0.002	1	< 0.001	< 0.001	< 0.001	< 0.001	1
	Treatment	3	29.256	< 0.001						
	Covariate	1	2.632	0.105						
2 am	Intercept	1	10.213	0.001	1	< 0.001	< 0.001	< 0.001	< 0.001	1
	Treatment	3	27.958	< 0.001						
	Covariate	1	1.006	0.316						

Observation time points	Generalized linear model				P values (Bonferroni adjusted pairwise comparisons)					
	Source of variation	*d.f.*	χ^2	*P*	CLBF *vs.* CLBM	CLBF *vs.* ALBF	CLBF *vs.* ALBM	CLBM *vs.* ALBF	CLBM *vs.* ALBM	ALBF *vs.* ALBM
4 am	Intercept	1	0.093	0.76	1	1	1	0.047	0.084	1
	Treatment	3	9.58	0.022						
	Covariate	1	4.327	0.038						
6 am	Intercept	1	0.045	0.832	1	0.196	0.959	0.005	0.047	1
	Treatment	3	13.416	0.004						
	Covariate	1	0.028	0.866						
8 am	Intercept	1	2.741	0.098	1	1	1	1	1	1
	Treatment	3	1.44	0.696						
	Covariate	1	0.104	0.747						
10 am	Intercept	1	14.714	< 0.001	1	1	0.09	0.335	0.007	0.993
	Treatment	3	11.18	0.011						
	Covariate	1	2.562	0.109						
12 pm	Intercept	1	18.024	< 0.001	1	1	1	1	1	1
	Treatment	3	0.446	0.931						
	Covariate	1	2.648	0.104						
2 pm	Intercept	1	10.853	0.001	0.892	1	1	0.169	1	0.959
	Treatment	3	5.227	0.156						
	Covariate	1	0.193	0.661						
4 pm	Intercept	1	0.421	0.516	1	0.353	< 0.001	0.273	< 0.001	0.073
	Treatment	3	18.949	< 0.001						
	Covariate	1	0.035	0.851						

Observation time points	Generalized linear model				*P* values (Bonferroni adjusted pairwise comparisons)					
	**Source of variation**	***d.f.***	χ^2	***P***	**CLBF *vs.* CLBM**	**CLBF *vs.* ALBF**	**CLBF *vs.* ALBM**	**CLBM *vs.* ALBF**	**CLBM *vs.* ALBM**	**ALBF *vs.* ALBM**
6 pm	Intercept	1	11.335	0.001	1	1	0.218	1	0.293	1
	Treatment	3	5.244	0.155						
	Covariate	1	9.265	0.002						
8 pm	Intercept	1	1.634	0.201	1	1	0.248	1	0.063	0.85
	Treatment	3	6.432	0.092						
	Covariate	1	2.834	0.092						
10 pm	Intercept	1	1.745	0.187	1	0.005	1	0.048	1	0.134
	Treatment	3	10.405	0.015						
	Covariate	1	0.194	0.66						
12 am	Intercept	1	0.504	0.478	0.377	< 0.001	0.001	0.036	0.265	1
	Treatment	3	23.883	< 0.001						
	Covariate	1	0.003	0.953						
2 am	Intercept	1	2.18	0.14	1	< 0.001	< 0.001	< 0.001	< 0.001	1
	Treatment	3	32.418	< 0.001						
	Covariate	1	2.945	0.086						
4 am	Intercept	1	1.957	0.162	0.96	0.199	0.434	0.004	0.011	1
	Treatment	3	15.094	0.002						
	Covariate	1	0.952	0.329						
6 am	Intercept	1	0.783	0.376	1	0.001	0.355	0.031	1	0.331
	Treatment	3	13.185	0.004						
	Covariate	1	1.509	0.219						

Observation time points	Generalized linear model				*P* values (Bonferroni adjusted pairwise comparisons)					
	**Source of variation**	***d.f.***	**χ^2**	***P***	**CLBF *vs*. CLBM**	**CLBF *vs*. ALBF**	**CLBF *vs*. ALBM**	**CLBM *vs*. ALBF**	**CLBM *vs*. ALBM**	**ALBF *vs*. ALBM**
8 am	Intercept	1	4.809	0.028	0.015	0.424	1	< 0.001	0.23	0.042
	Treatment	3	22.44	< 0.001						
	Covariate	1	2.002	0.157						
10 am	Intercept	1	45.553	< 0.001	1	1	0.588	1	0.054	0.723
	Treatment	3	6.582	0.086						
	Covariate	1	4.996	0.025						
12 pm	Intercept	1	27.61	< 0.001	1	0.412	< 0.001	1	0.003	0.022
	Treatment	3	18.541	< 0.001						
	Covariate	1	11.168	0.001						

Table S2. The amounts (ng) of male-produced aggregation-sex pheromone components, 4-(*n*-heptyloxy)butanal and 4-(*n*-heptyloxy)butan-1-ol, released by 16 male *Anoplophora glabripennis* adults in different periods of a day

Beetle #	The amounts of 4-(*n*-heptyloxy)butanal released within 4h						The amounts of 4-(*n*-heptyloxy)butan-1-ol released within 4h					
	12AM-4AM	4AM-8AM	8AM-12PM	12PM-4PM	4PM-8PM	8PM-12AM	12AM-4AM	4AM-8AM	8AM-12PM	12PM-4PM	4PM-8PM	8PM-12AM
1	0	0	0	51.9	0	0	0	0	0	42.34	0	0
2	0	0	0	0	0	0	0	0	0	0	0	0
3	20.56	14.84	144.81	455.04	175.64	49.9	81.12	30.69	165.13	706.85	257.43	196.55
4	0	12.16	0	0	0	0	0	0	0	0	0	16.33
5	12.1	0	41.43	184.43	28.08	11.05	19.69	0	45.26	286.78	52.49	27.47
6	58.48	24.55	132.67	312.4	37.24	137.83	224.56	62.48	317.55	762.9	76.91	474.71
7	62.57	27.83	1121.7	404.54	206.67	18.88	123.28	138.4	1866.49	942.76	379.87	102.48
8	0	0	83.44	239.69	30.46	0	0	0	26.09	236.03	25.97	0
9	0	0	16.2	0	0	0	0	0	30.31	0	0	0
10	0	0	0	18.73	71.49	0	0	0	16.99	38.17	147.64	0
11	0	0	7.6	0	0	0	0	0	23.42	19.63	0	0
12	0	0	59.93	54.65	112.73	0	0	0	43.14	46.33	123.55	0
13	65.87	24.47	262.55	341.94	278.82	64.21	29.97	17.18	614.21	473.56	352.81	47.52
14	112.66	122.94	304.51	389.74	333.6	246.09	59.2	46.24	366.12	797.39	534.74	184.18
15	36.06	10.15	258.07	18.25	15.31	66.37	99.97	25.09	189.49	18.55	0	305.01
16	205.69	59.61	334.77	588.52	410.58	0	491.32	142.16	590.29	1109.17	714.52	0

Table S3. The amounts (ng) of male-produced aggregation-sex pheromone components, 4-(*n*-heptyloxy)butanal and 4-(*n*-heptyloxy)butan-1-ol, released by 16 male *Anoplophora chinensis* adults in different periods of a day

Beetle #	The amounts of 4-(*n*-heptyloxy)butanal released within 4h						The amounts of 4-(*n*-heptyloxy)butan-1-ol released within 4h					
	12AM-4AM	4AM-8AM	8AM-12PM	12PM-4PM	4PM-8PM	8PM-12AM	12AM-4AM	4AM-8AM	8AM-12PM	12PM-4PM	4PM-8PM	8PM-12AM
1	0	15.37	0	33.77	144.08	58.16	203.98	40.21	34.94	226.65	1543.27	656.07
2	0	0	0	958.47	59.51	0	0	0	0	5863.08	43.99	0
3	31.39	0	18.27	53.47	364.52	109.19	178.61	106.23	67.53	339.7	2209.93	1229.7
4	36.21	9.99	60.66	148.84	460.07	46.7	103.36	17.97	53.95	805.15	1814.55	200.14
5	46.4	33.4	16.21	405.58	609.37	46.77	208.25	50.17	16.85	1398.9	1522.56	339.14
6	43.73	41.75	19.04	130.29	886.6	255.93	153.66	85.81	24.18	293.96	4583.53	644.32
7	0	0	0	0	0	0	0	0	0	0	0	0
8	55.84	22.55	19.31	13.45	140.25	154.15	244.1	44.98	26.13	15.99	170.3	1439.24
9	69.23	50.29	23.95	190.73	1122.44	249.29	254.94	126.69	31.91	411.3	3204.23	1113.36
10	50.23	14.74	265	643.61	675.89	68.78	50.83	14.25	534.37	2204.28	1483.69	219.62
11	12.33	0	0	66.54	1094.76	19.56	20.7	0	0	83.4	1501.85	25.27
12	45.1	51.22	14.72	318.87	999.67	175.77	39.78	33.16	16.88	748.63	1979.33	441.29
13	24.9	20.03	149.17	110.43	514.73	116.89	83.59	28.87	236.43	166.19	1959.28	694.24
14	196.91	93.47	133.47	328.55	680.05	395.57	150.67	31.34	32.09	631.38	2741.67	1481.1
15	131.31	27.77	39.05	23.79	1105.82	208.7	300.41	47.53	30.66	17.08	4061.53	1018.07
16	32.07	17.42	21.69	149.83	440.25	127.33	72.31	23.11	20.9	504.21	2264.53	573.73

Figure S1: the positions and mounting pairs of beetles observed in the cages at the time points from July 23 to July 25 2023

In figure:

AF: female *Anoplophora glabripennis*

AM: male *Anoplophora glabripennis*

CF: female *Anoplophora chinensis*

CM: male *Anoplophora chinensis*

"×": mounting

1

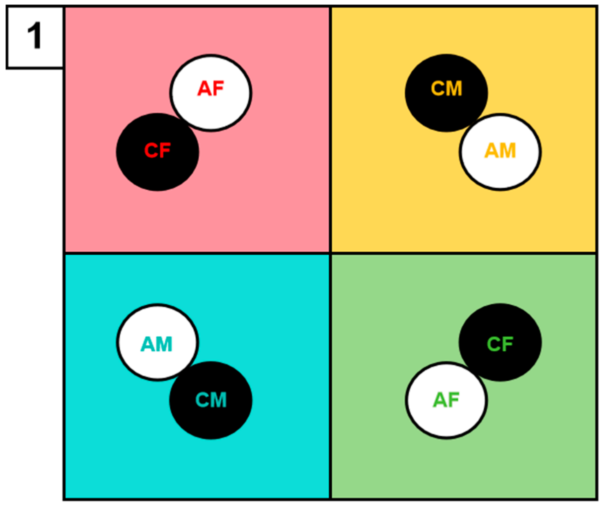

2

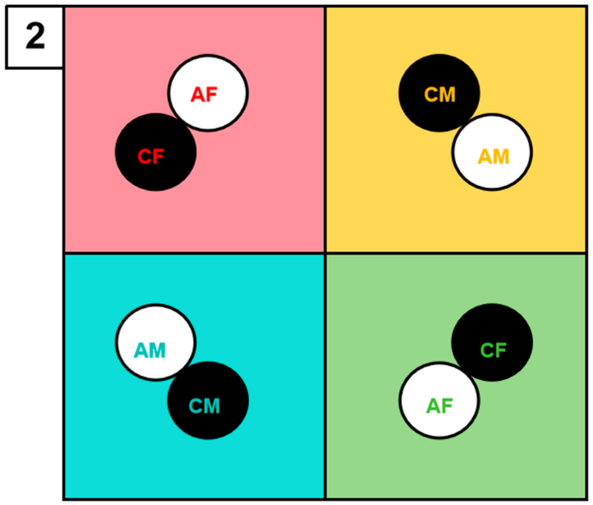

3

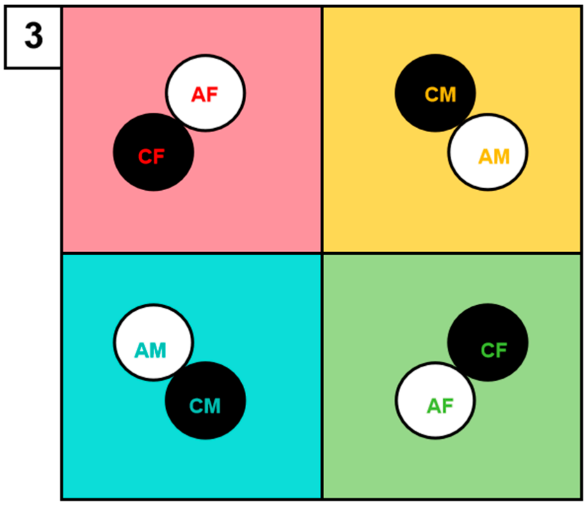

4

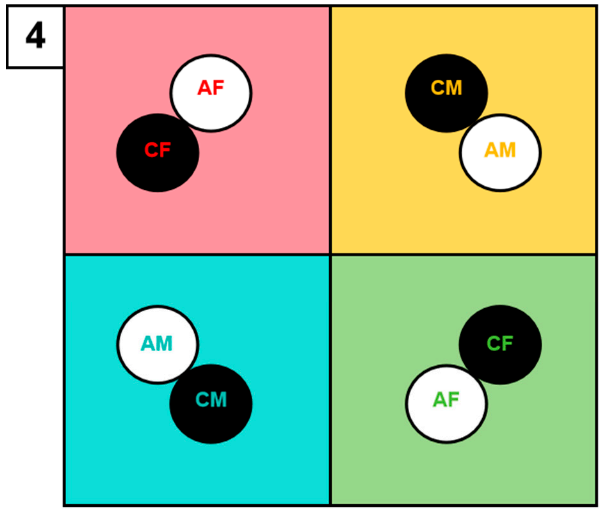

5

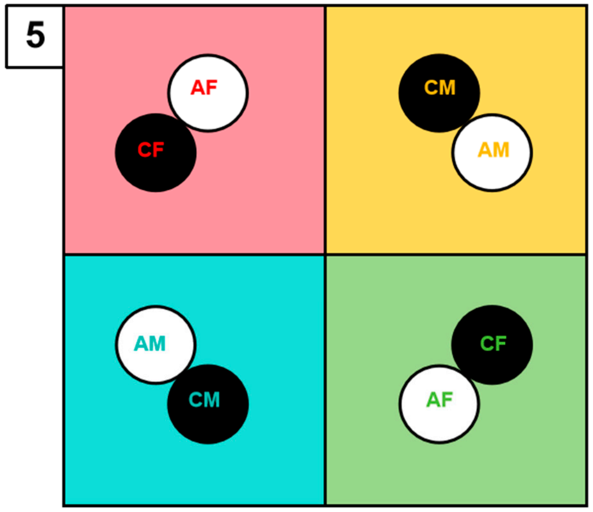

2023.7.23

12 PM

Release point

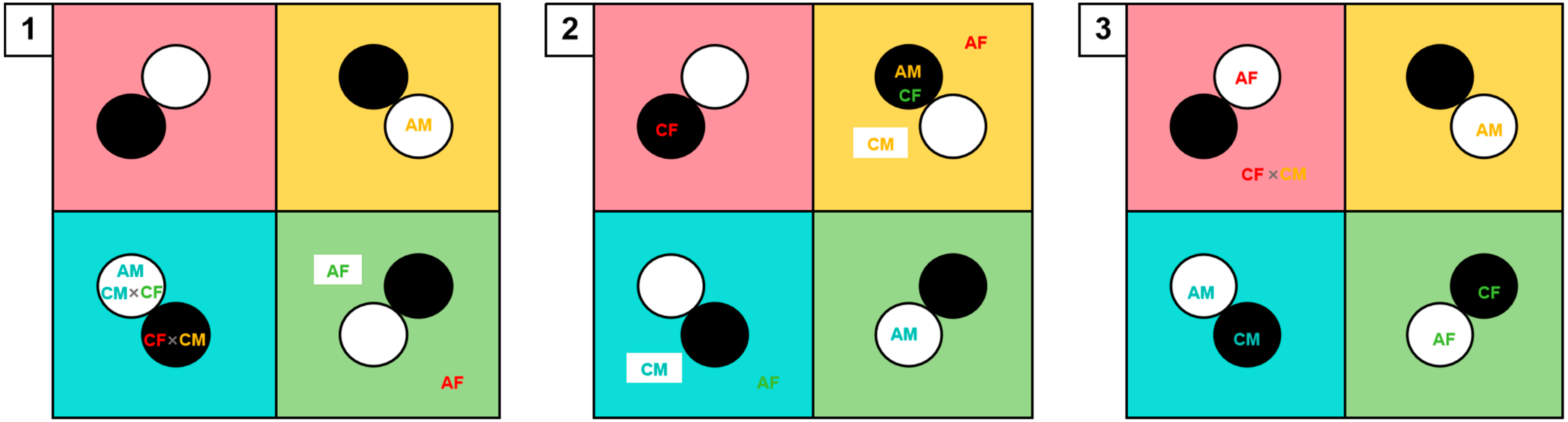

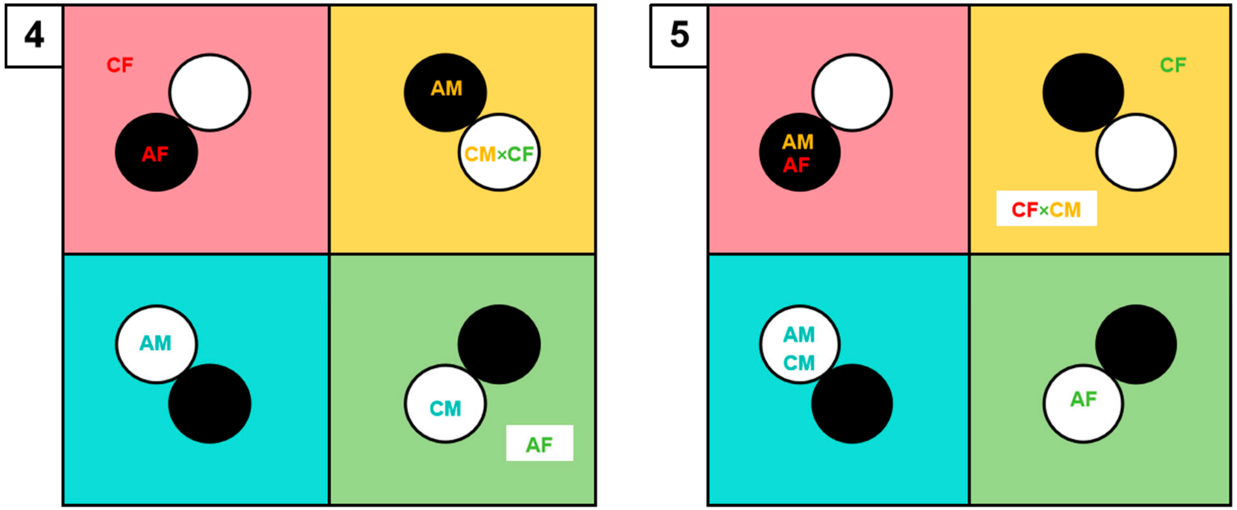

2023.7.23

2 PM

27.3℃, 72%RH

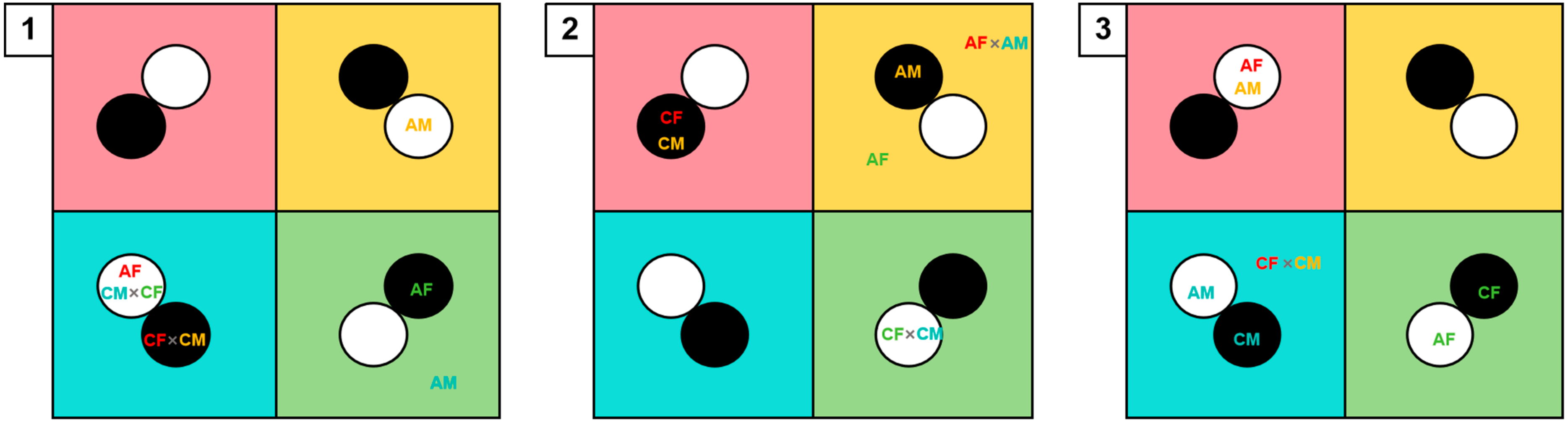

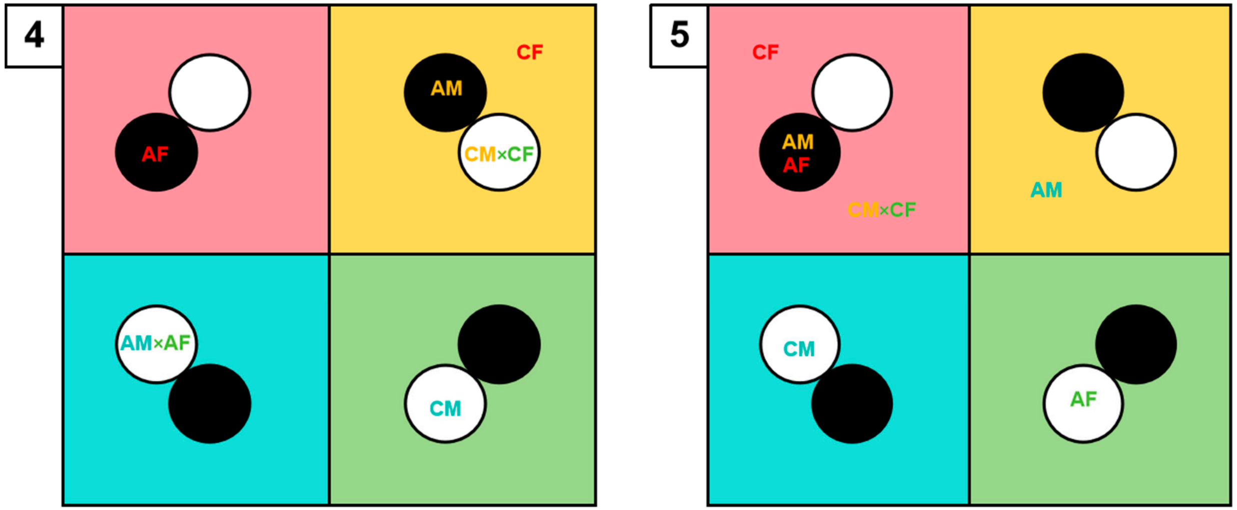

2023.7.23

4 PM

27.7℃, 69%RH

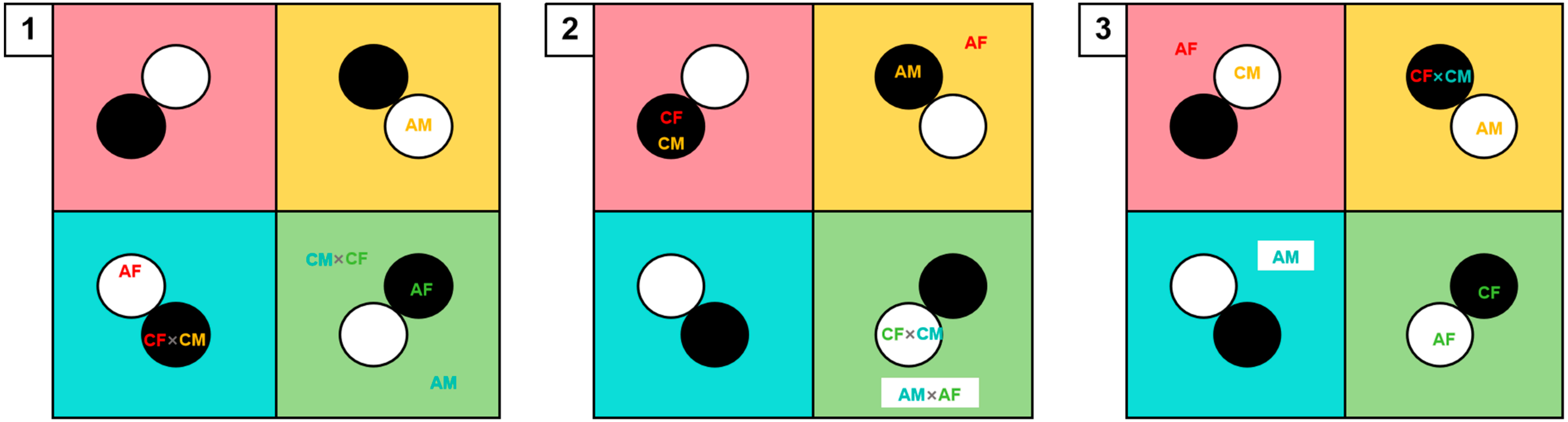

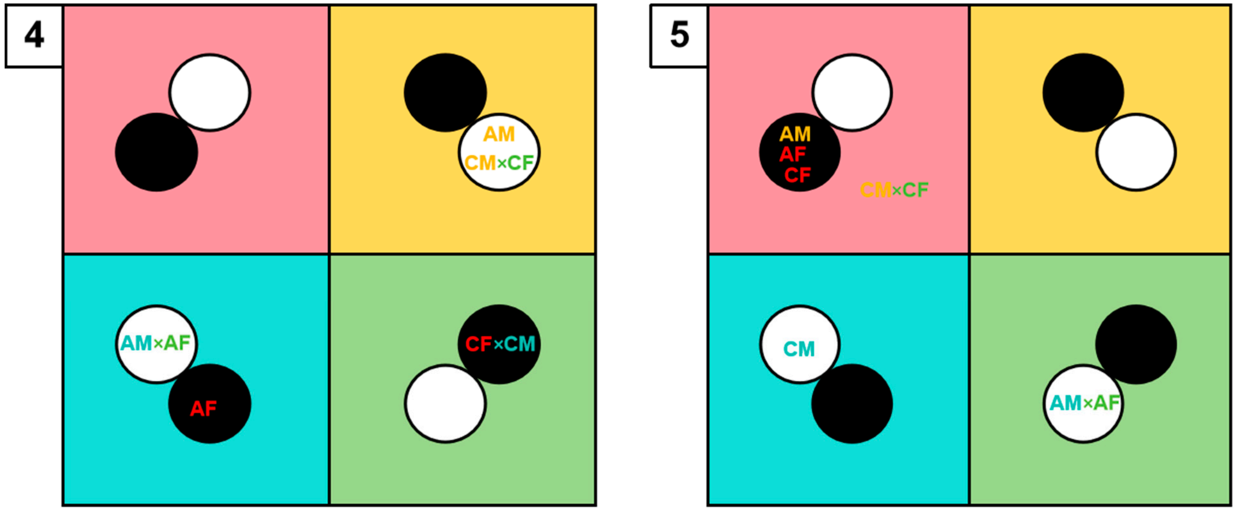

2023.7.23

6 PM

27℃, 78%RH

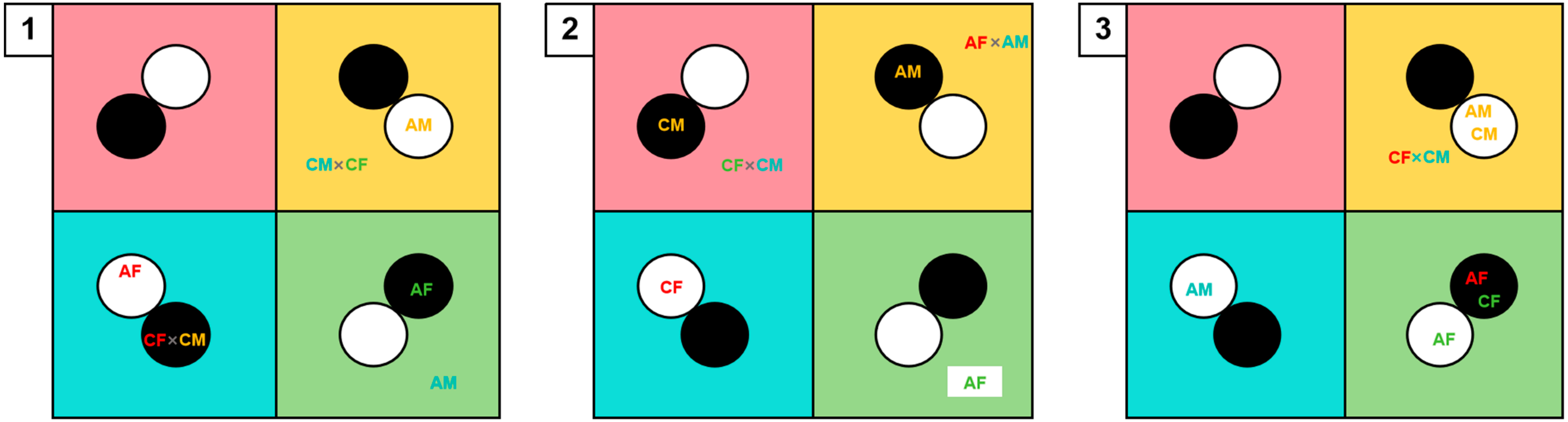

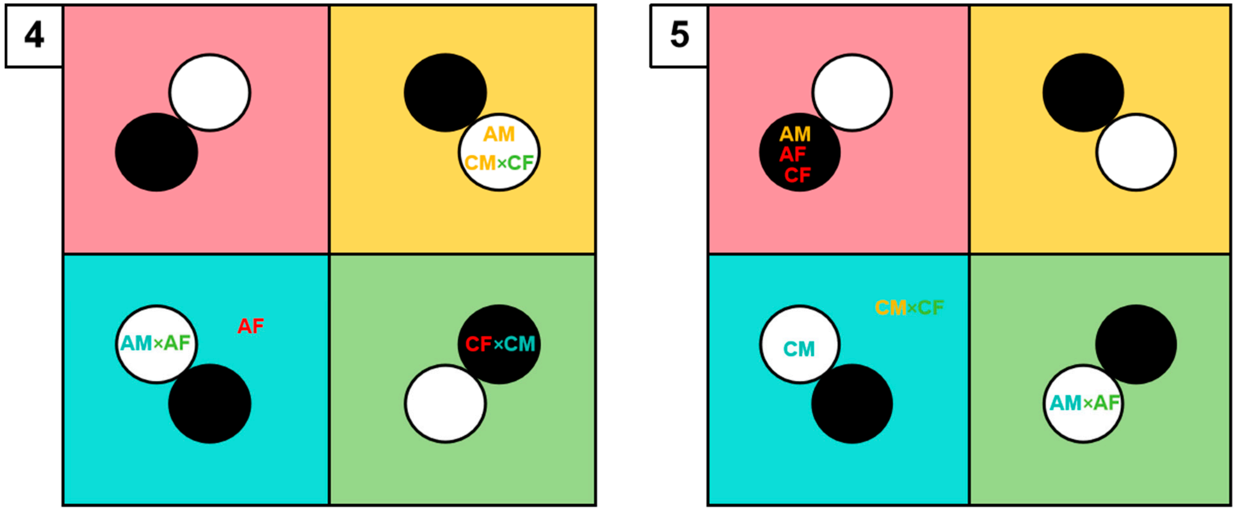

2023.7.23

8 PM

26.3℃, 86%RH

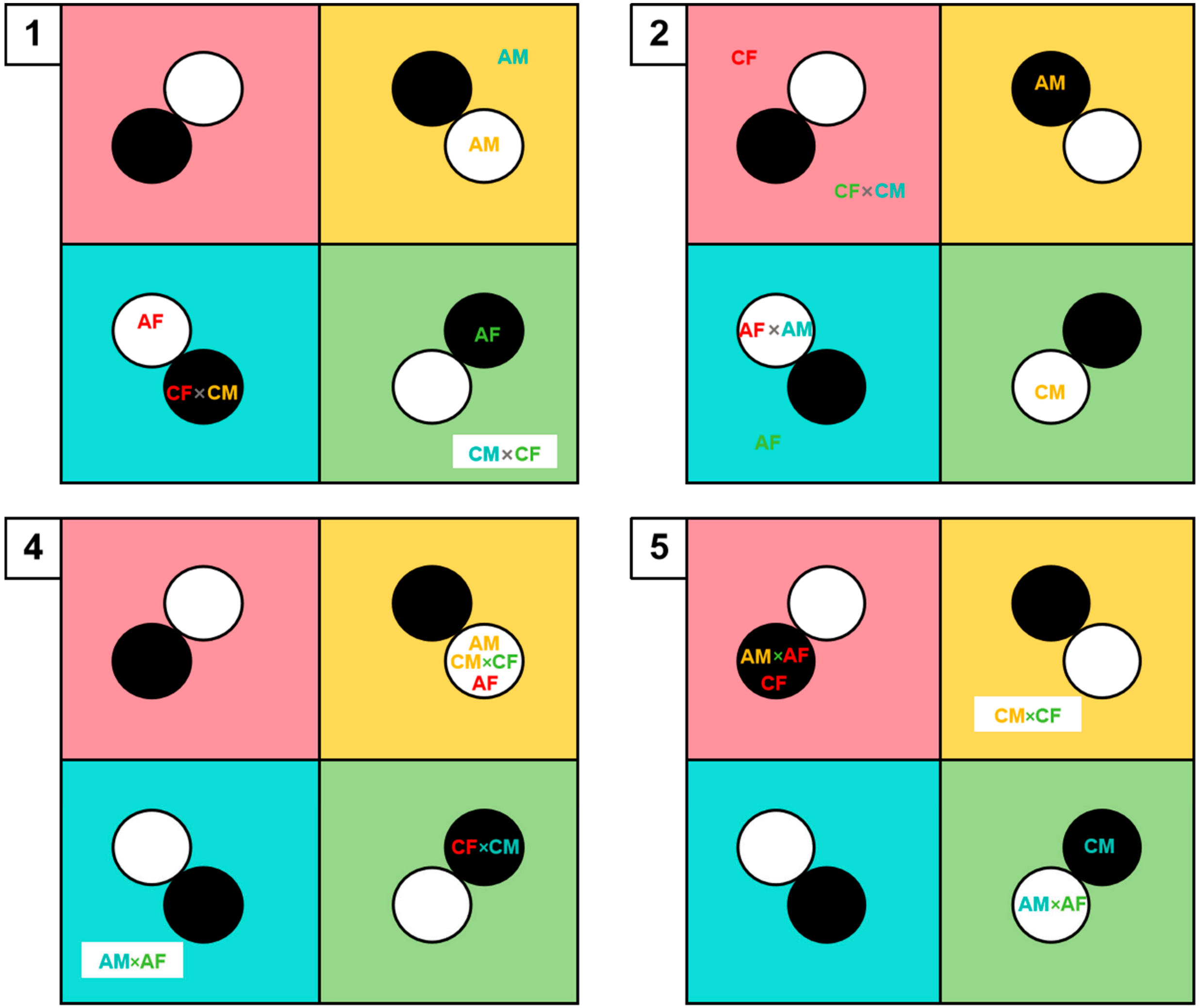

2023.7.23

10 PM

25.9℃, 93%RH

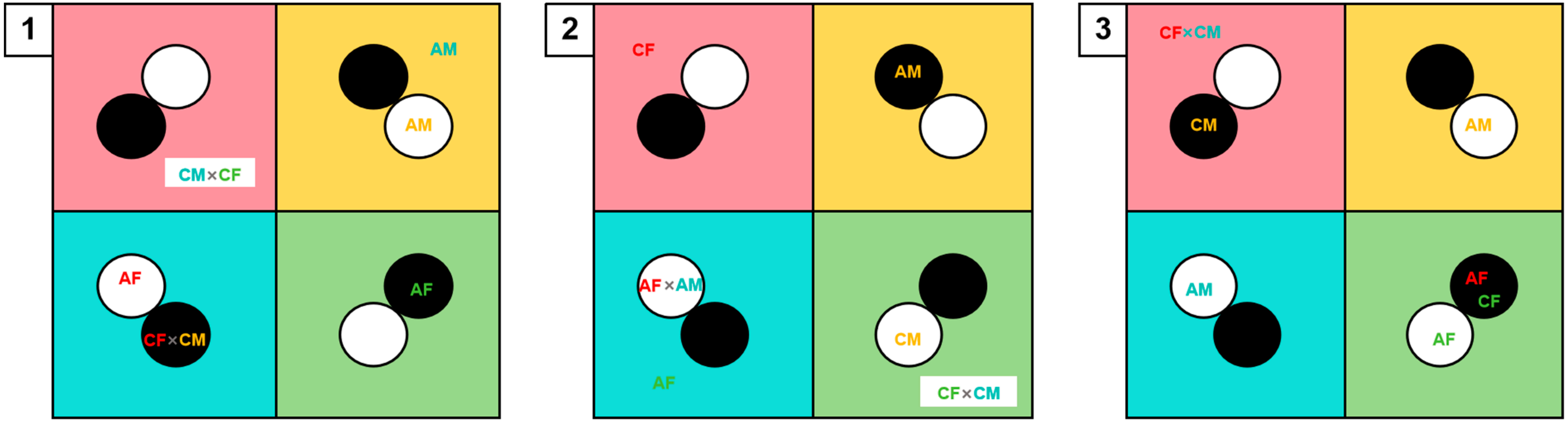

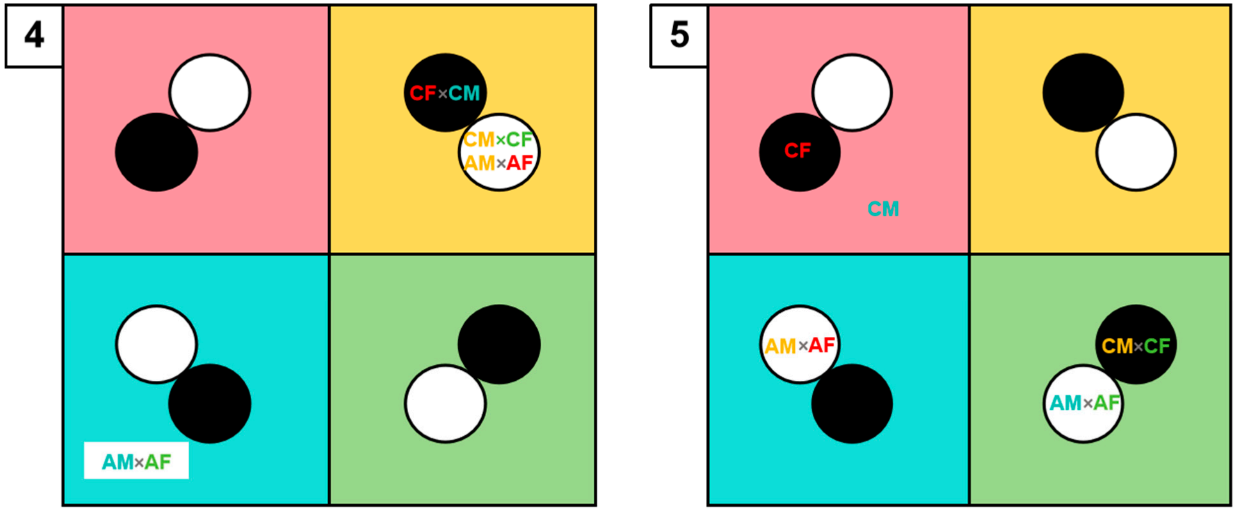

2023.7.24

12 AM

25.6℃, 97%RH

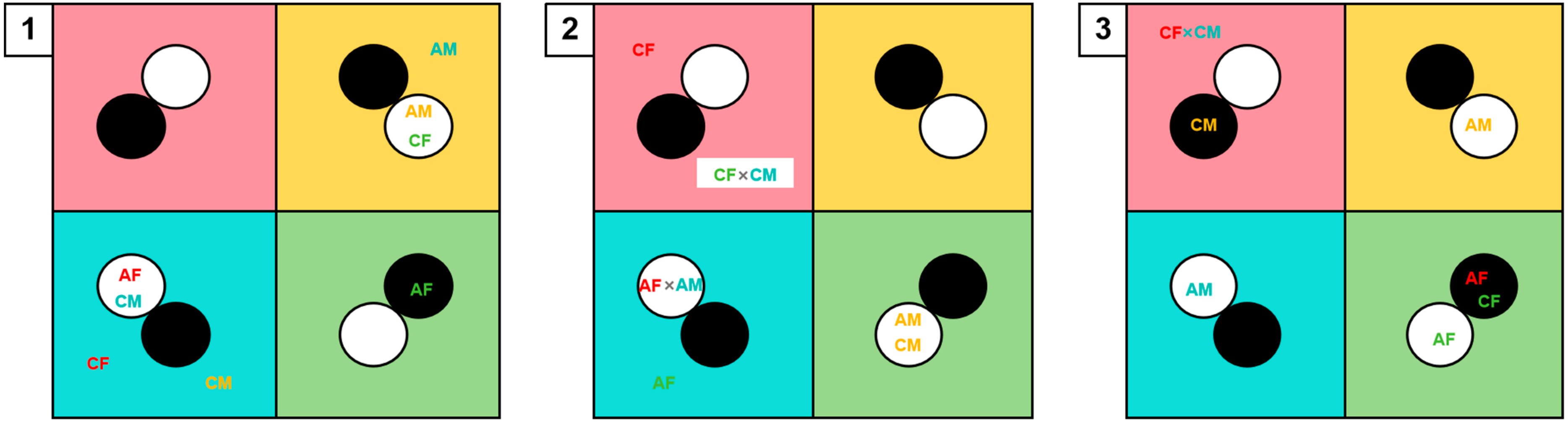

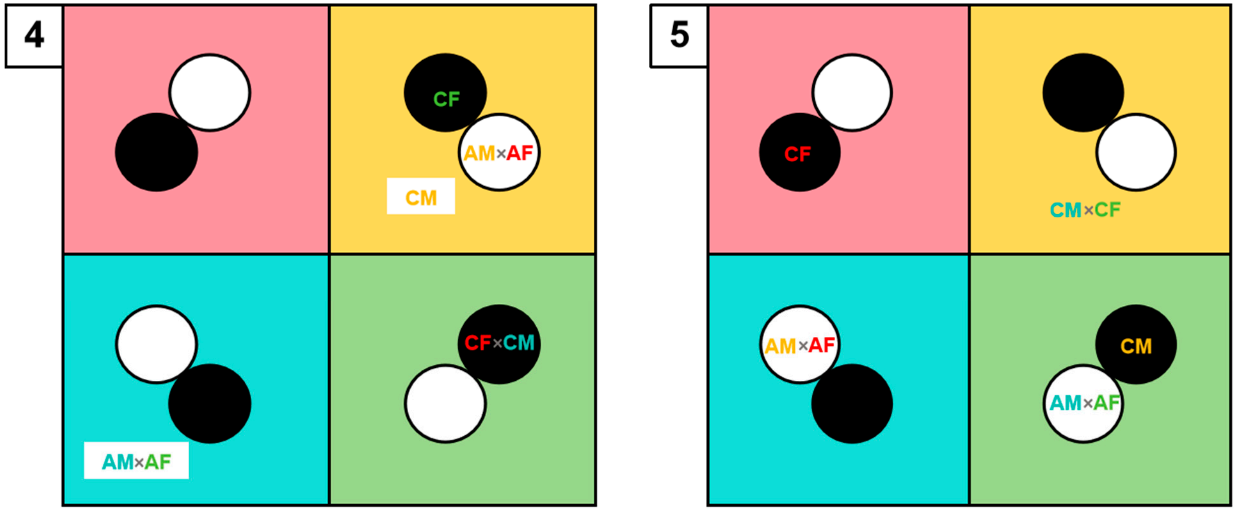

2023.7.24

2 AM

25.3℃, 99%RH

Light rain

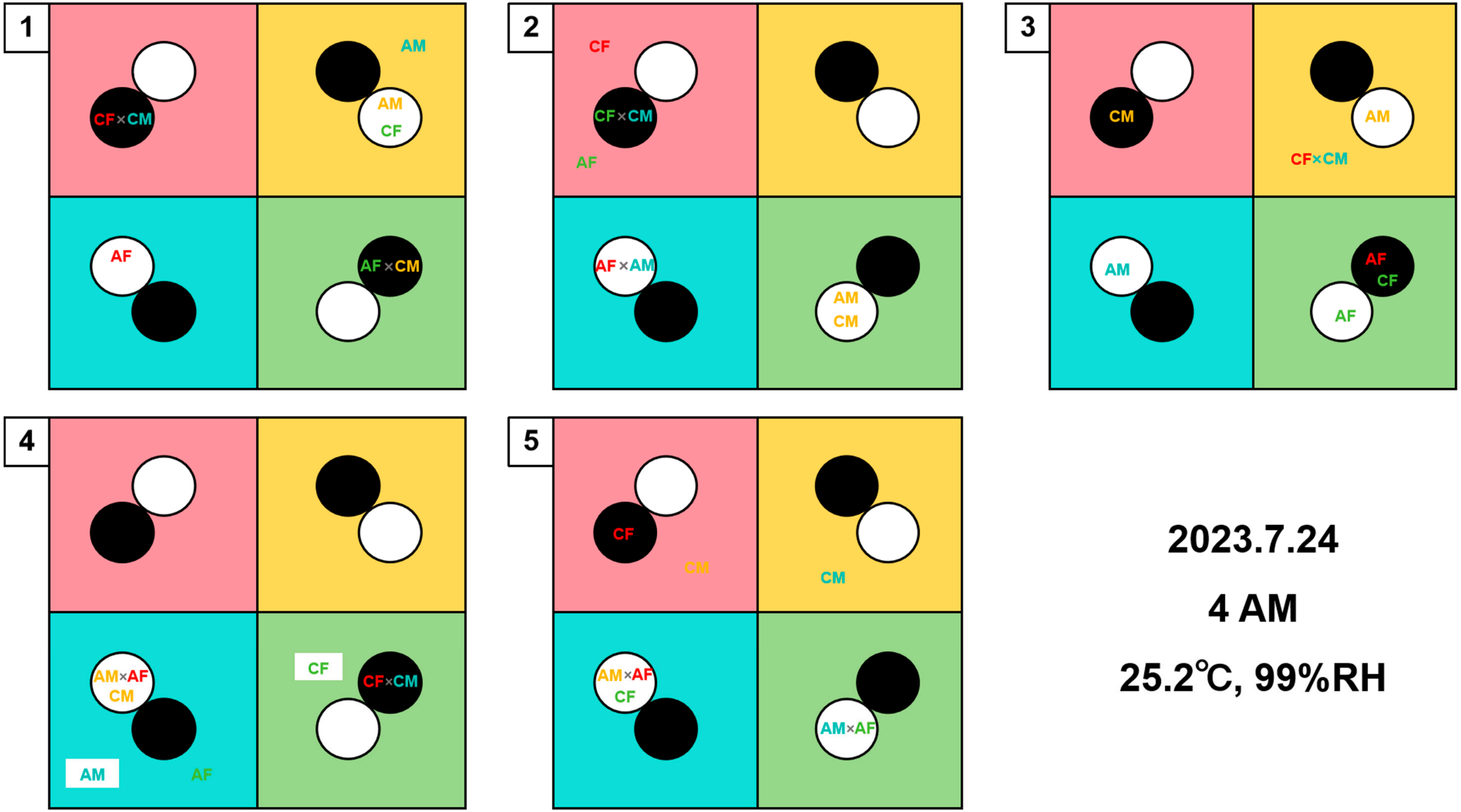

2023.7.24

4 AM

25.2℃, 99%RH

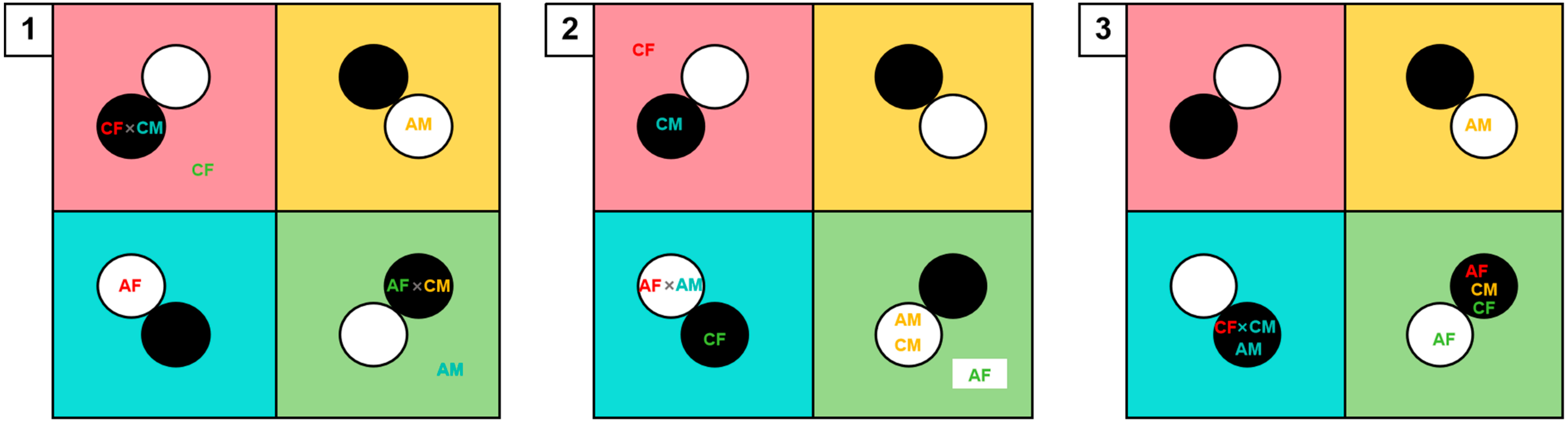

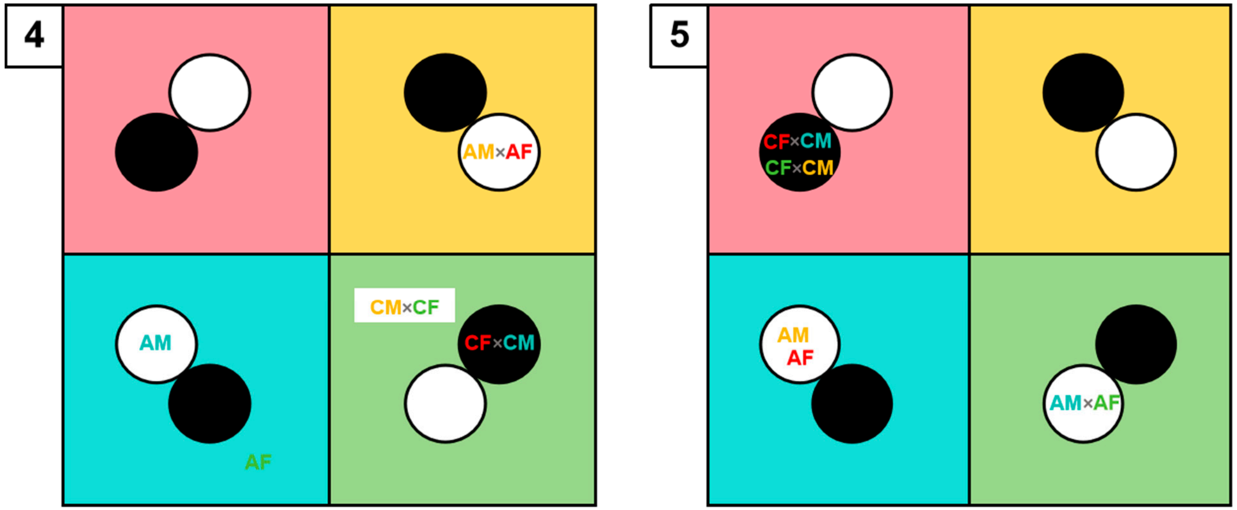

2023.7.24

6 AM

24.9℃, 99%RH

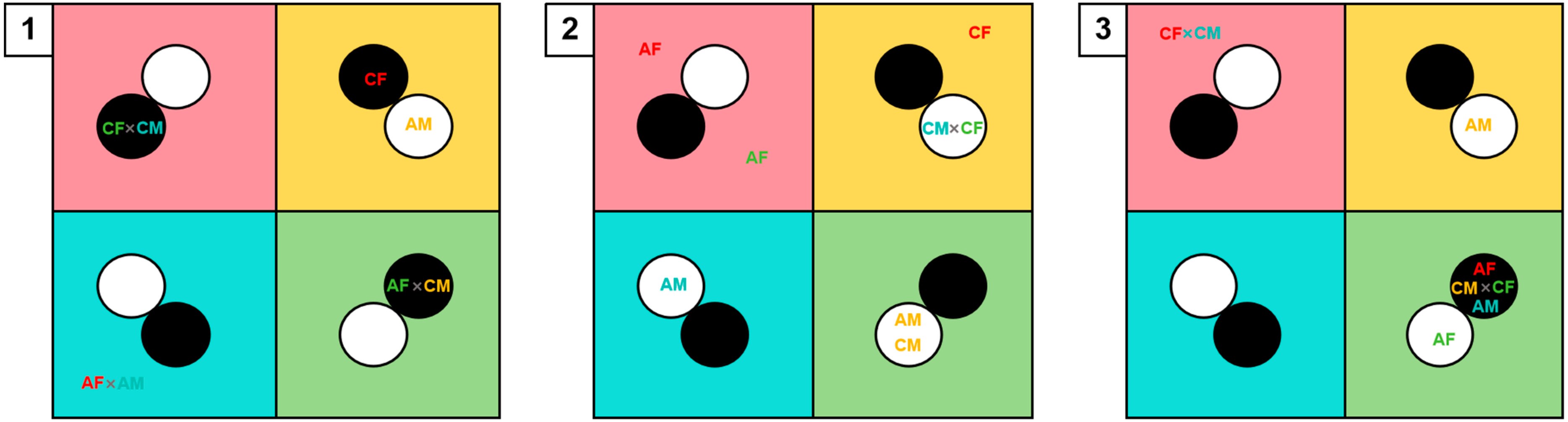

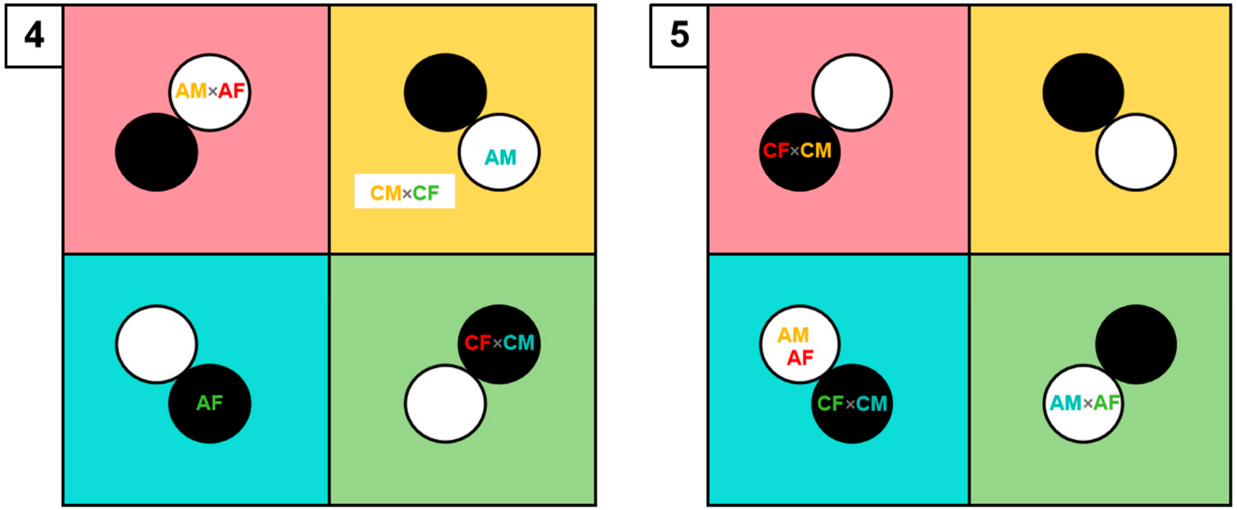

2023.7.24

8 AM

25.7℃, 99%RH

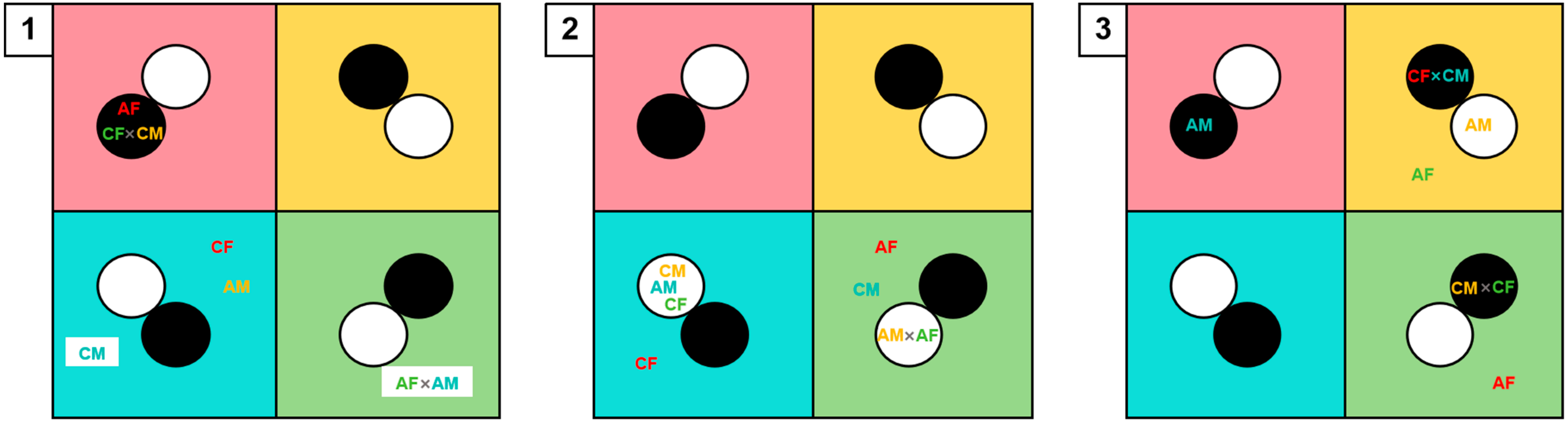

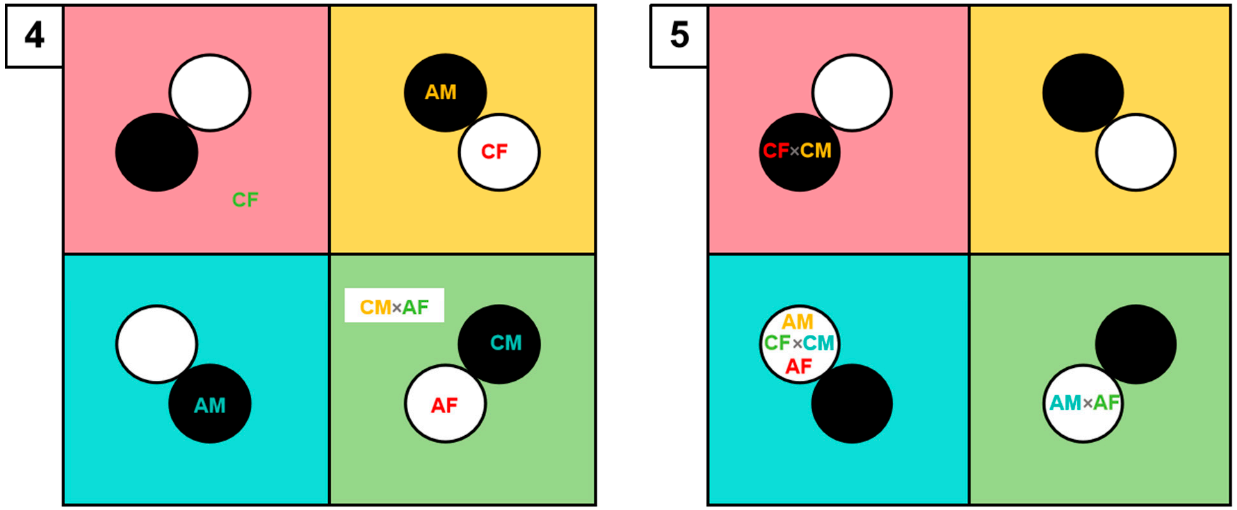

2023.7.24

10 AM

27.3℃, 79%RH

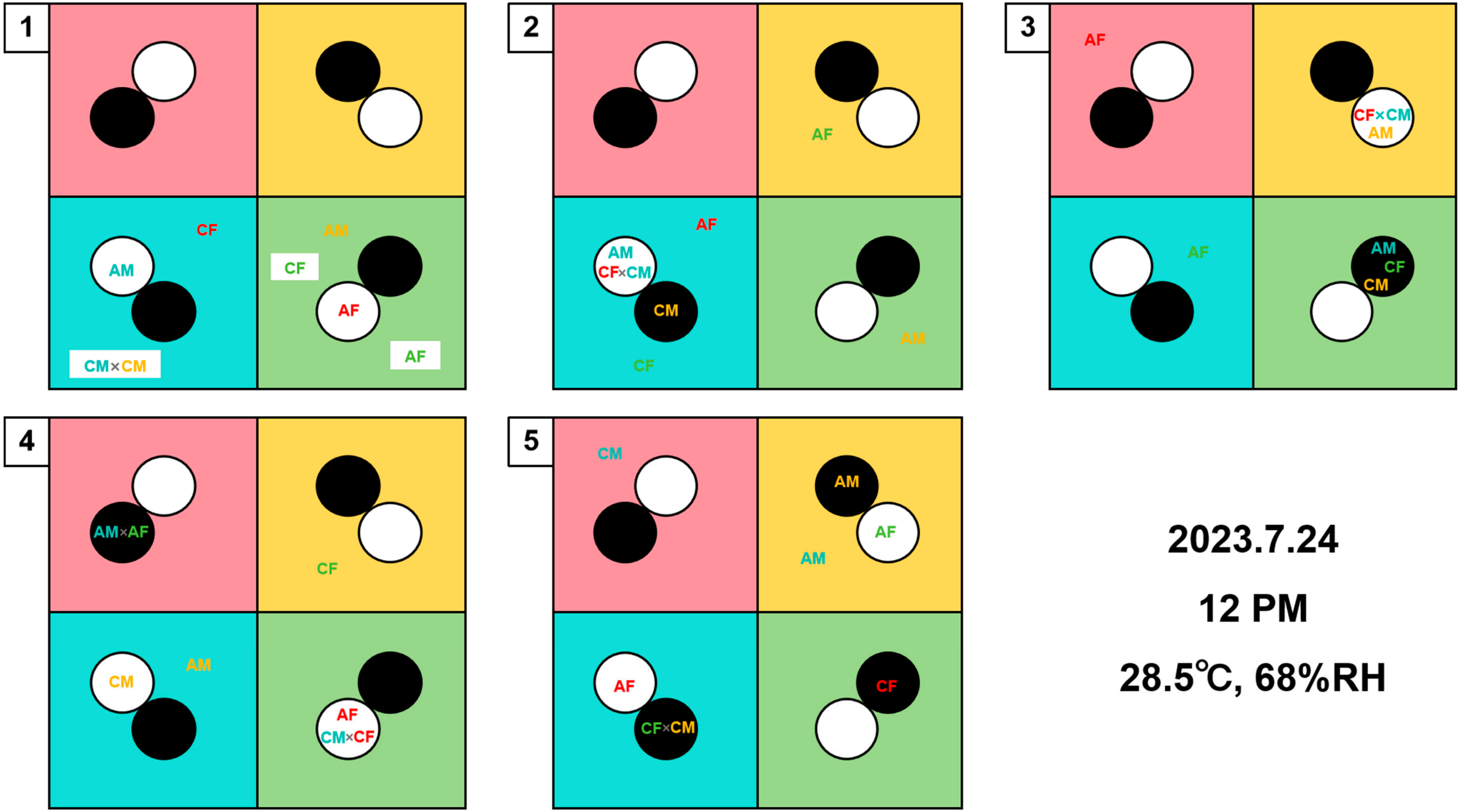

2023.7.24

12 PM

28.5℃, 68%RH

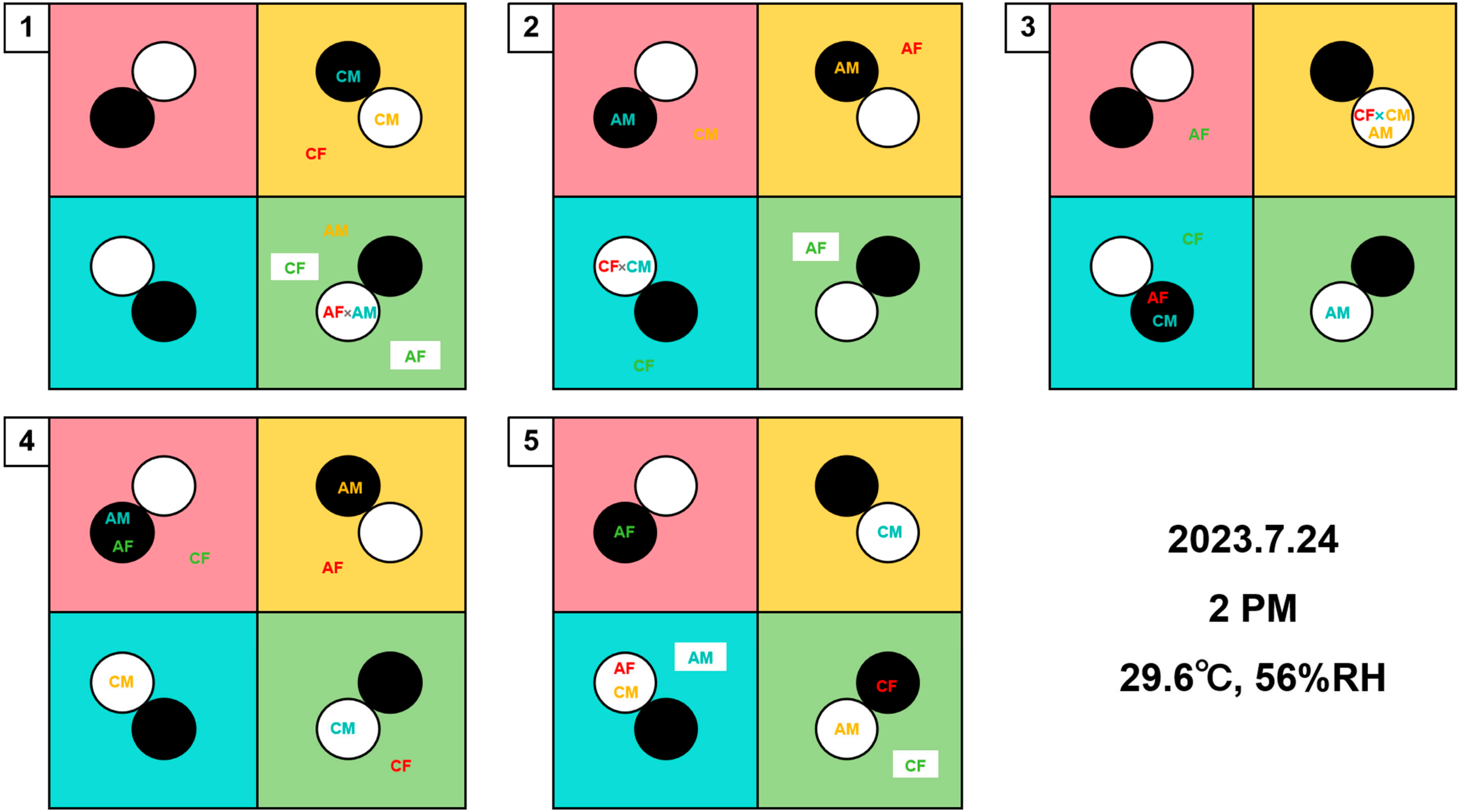
1
CM
CM
CF
AM
CF
AF×AM
AF
2
AM
CM
AM
AF
CF×CM
CF
AF
3
AF
CF×CM
AM
CF
AF
CM
AM
4
AM
AF
CF
AM
AF
CM
CM
CF
5
AF
CM
AM
AF
CM
CF
AM
CF
2023.7.24
2 PM
29.6℃, 56%RH

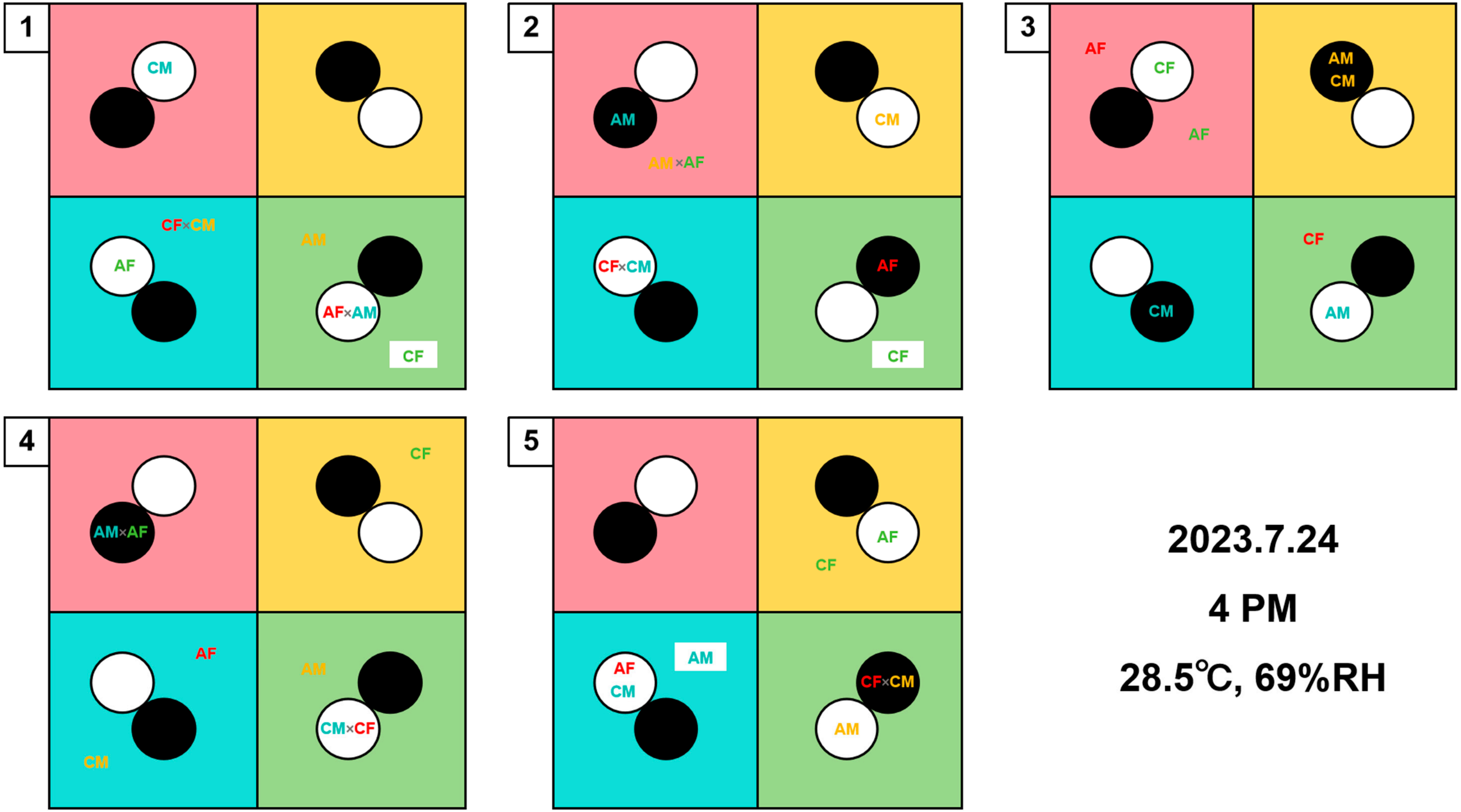
1
CM
CF×CM
AF
AM
AF×AM
CF
2
AM
AM×AF
CM
CF×CM
AF
CF
3
AF
CF
AF
AM
CM
CM
CF
AM
4
AM×AF
CF
AF
CM
AM
CM×CF
5
AF
CF
AF
CM
AM
CF×CM
AM
2023.7.24
4 PM
28.5℃, 69%RH

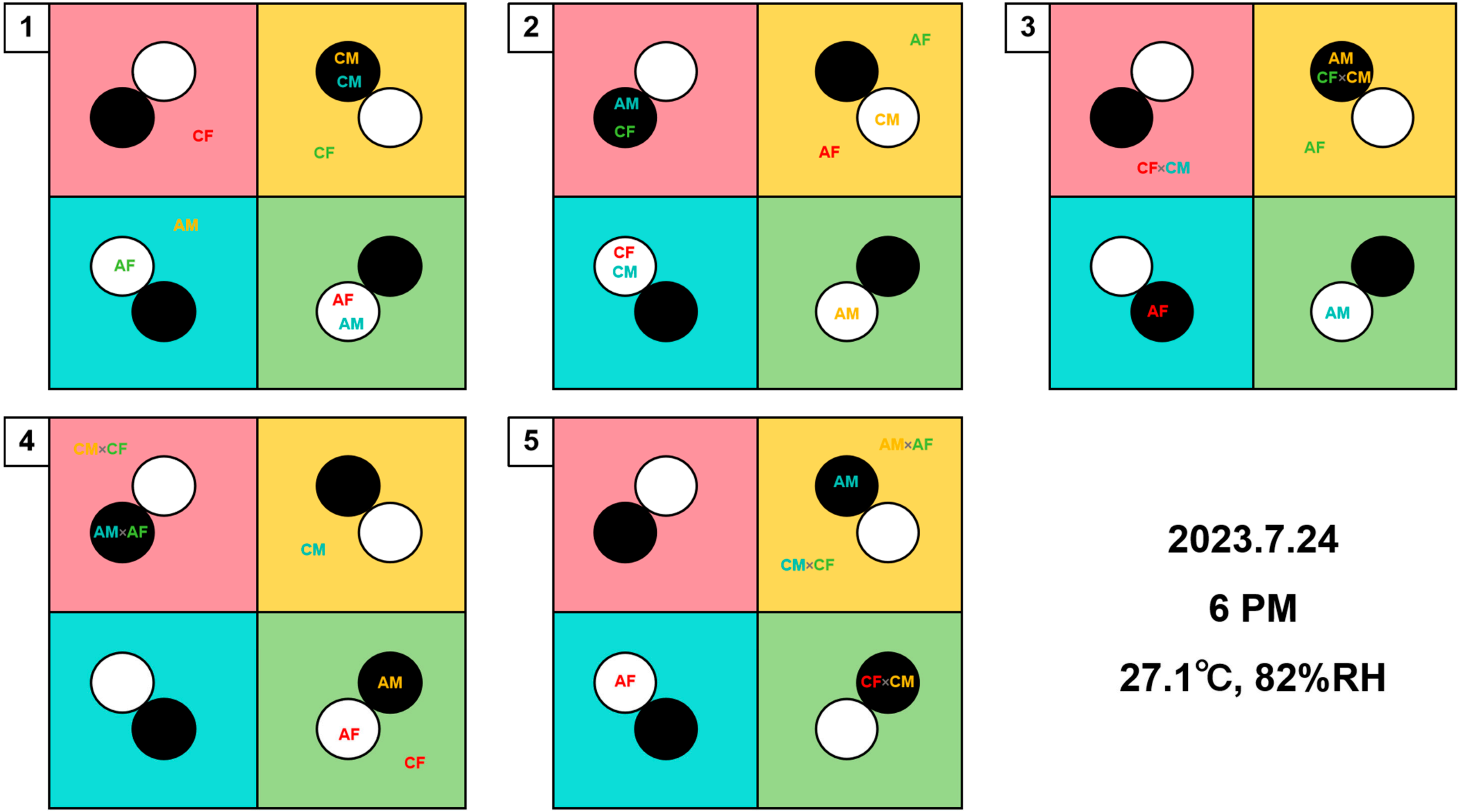
1
CM
CM
CF
CF
AM
AF
AF
AM
2
AF
AM
CF
CM
AF
CF
CM
AM
3
AM
CF×CM
AF
CF×CM
AF
AM
4
CM×CF
AM×AF
CM
AM
AF
CF
5
AM×AF
AM
CM×CF
AF
CF×CM
2023.7.24
6 PM
27.1℃, 82%RH

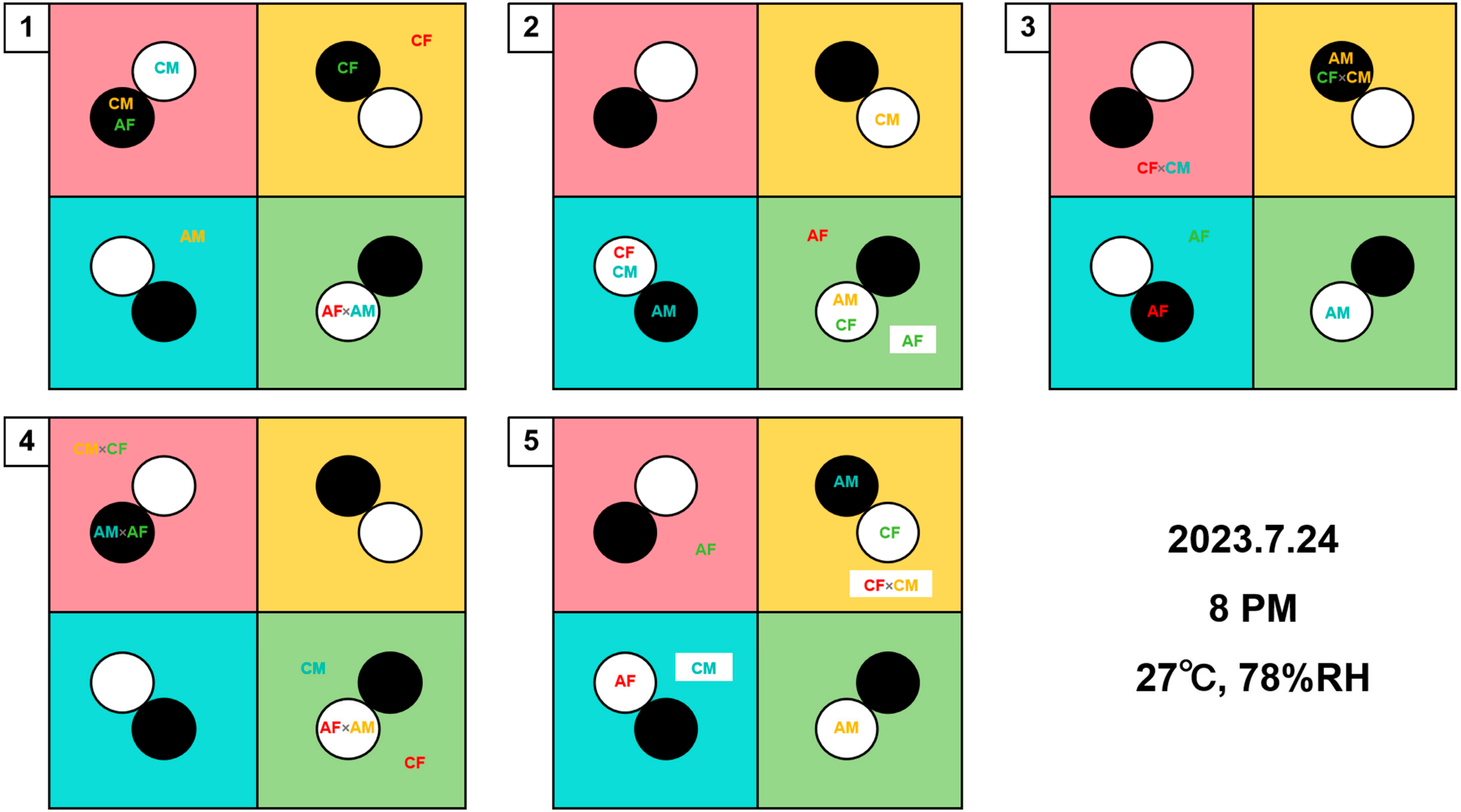

2023.7.24

8 PM

27℃, 78%RH

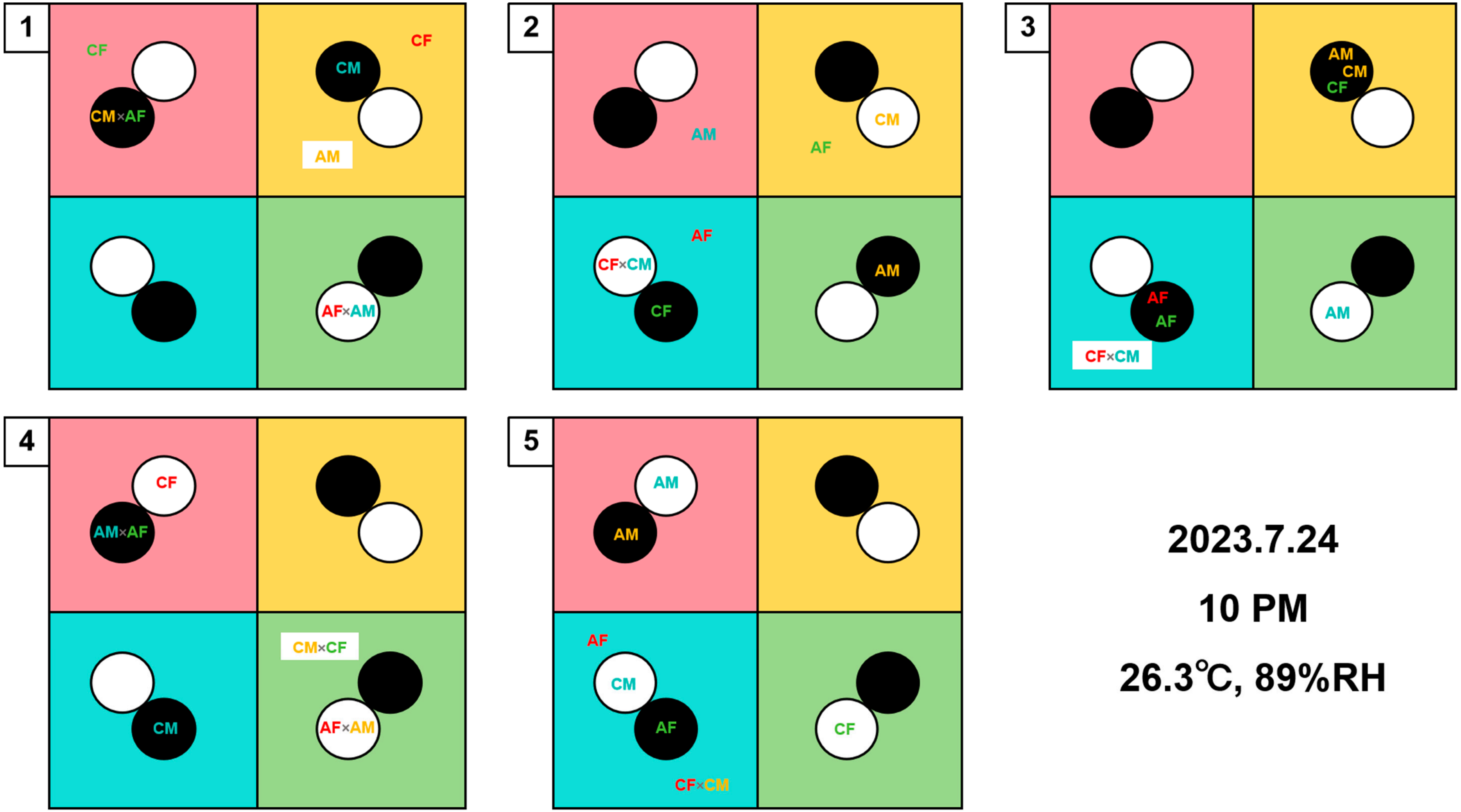

2023.7.24

10 PM

26.3℃, 89%RH

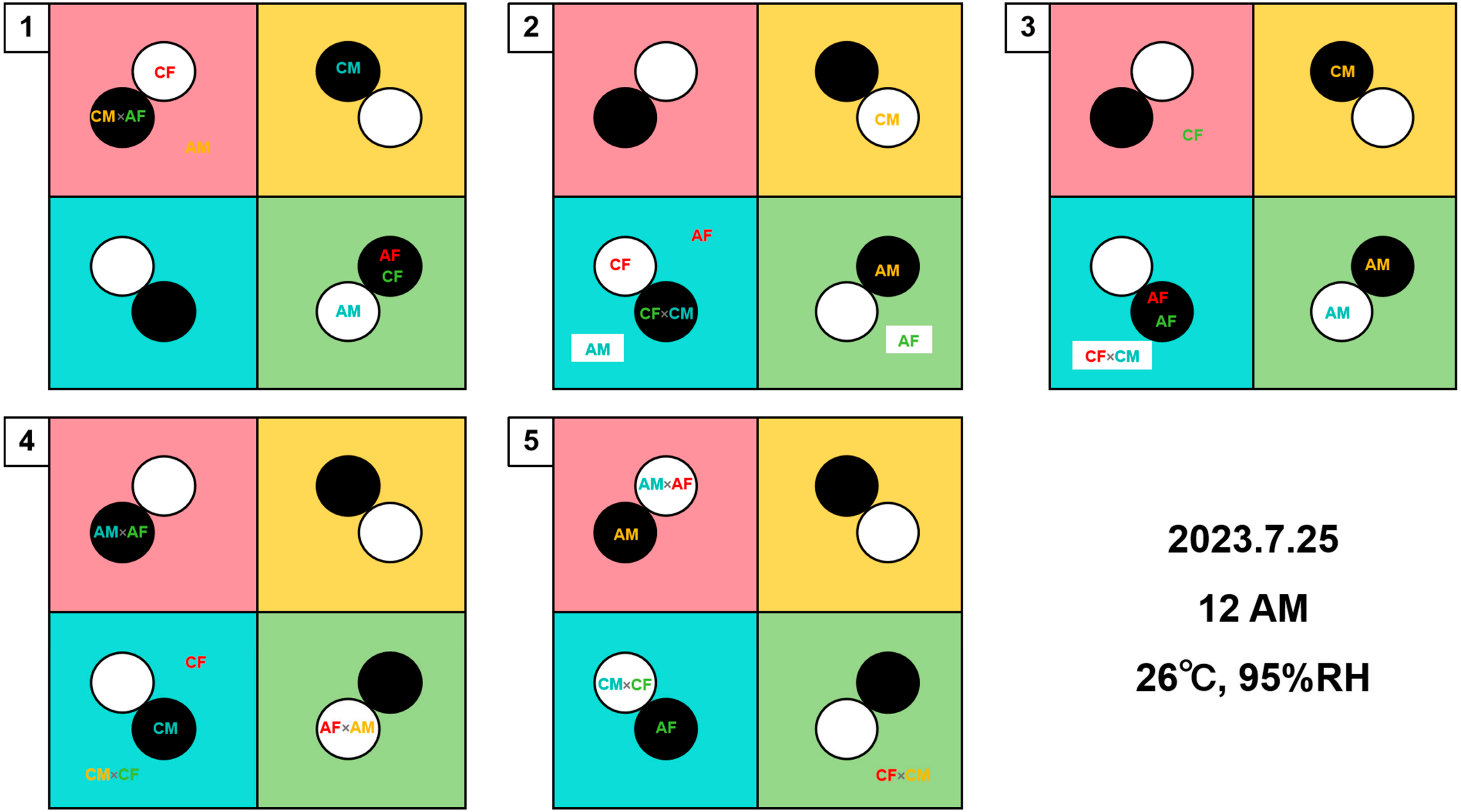
1
CF
CM×AF
AM
CM
AF
CF
AM
2
CM
AF
CF
CF×CM
AM
AM
AF
3
CM
CF
AF
AF
CF×CM
AM
AM
4
AM×AF
CF
CM
CM×CF
AF×AM
5
AM×AF
AM
CM×CF
AF
CF×CM
2023.7.25
12 AM
26℃, 95%RH

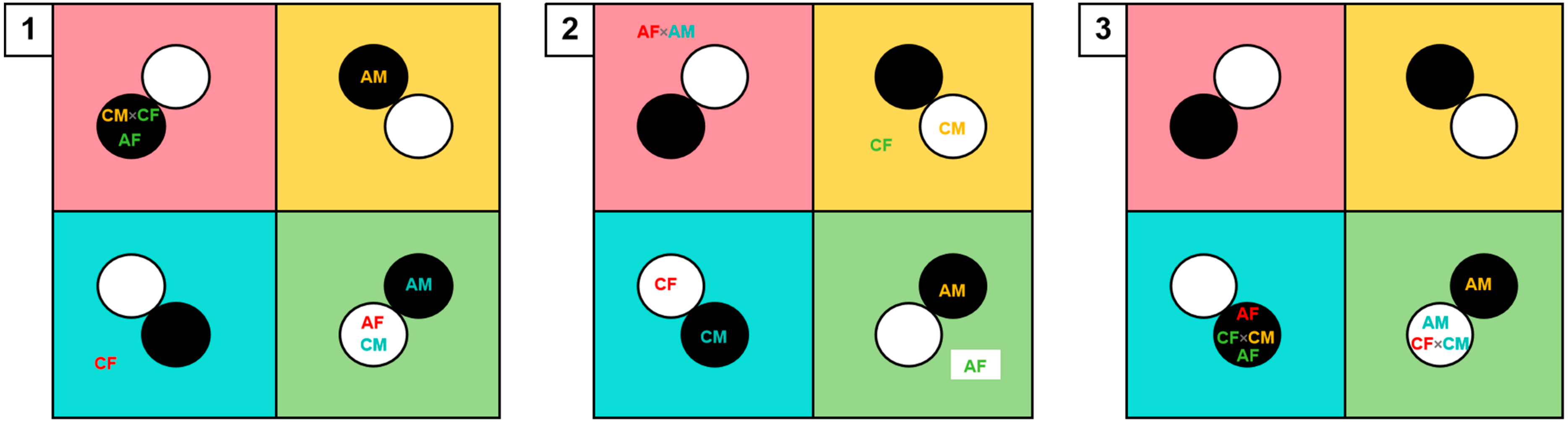

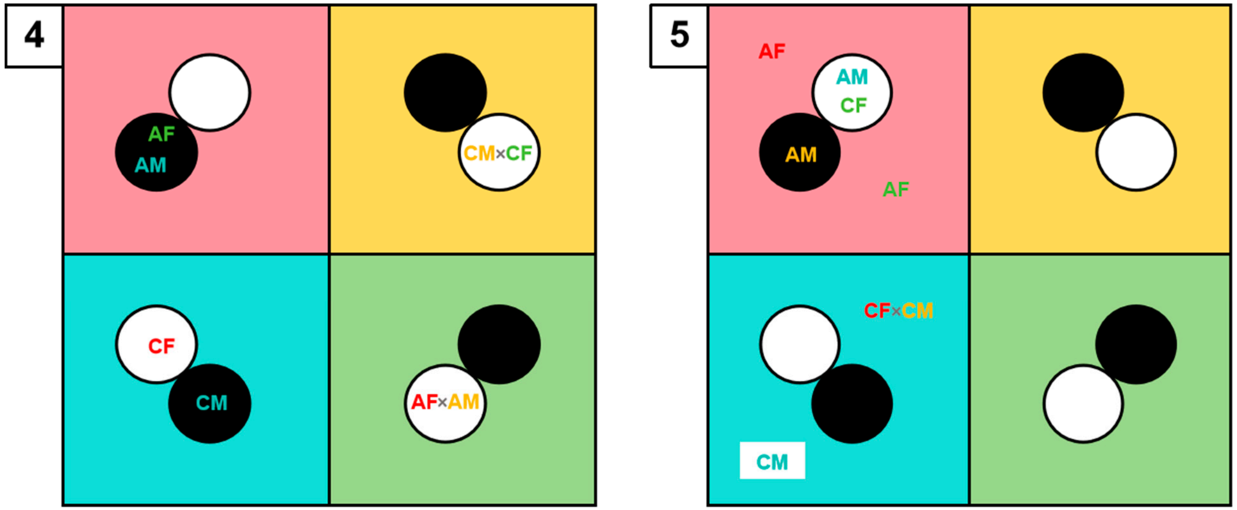

2023.7.25

2 AM

25.7℃, 99%RH

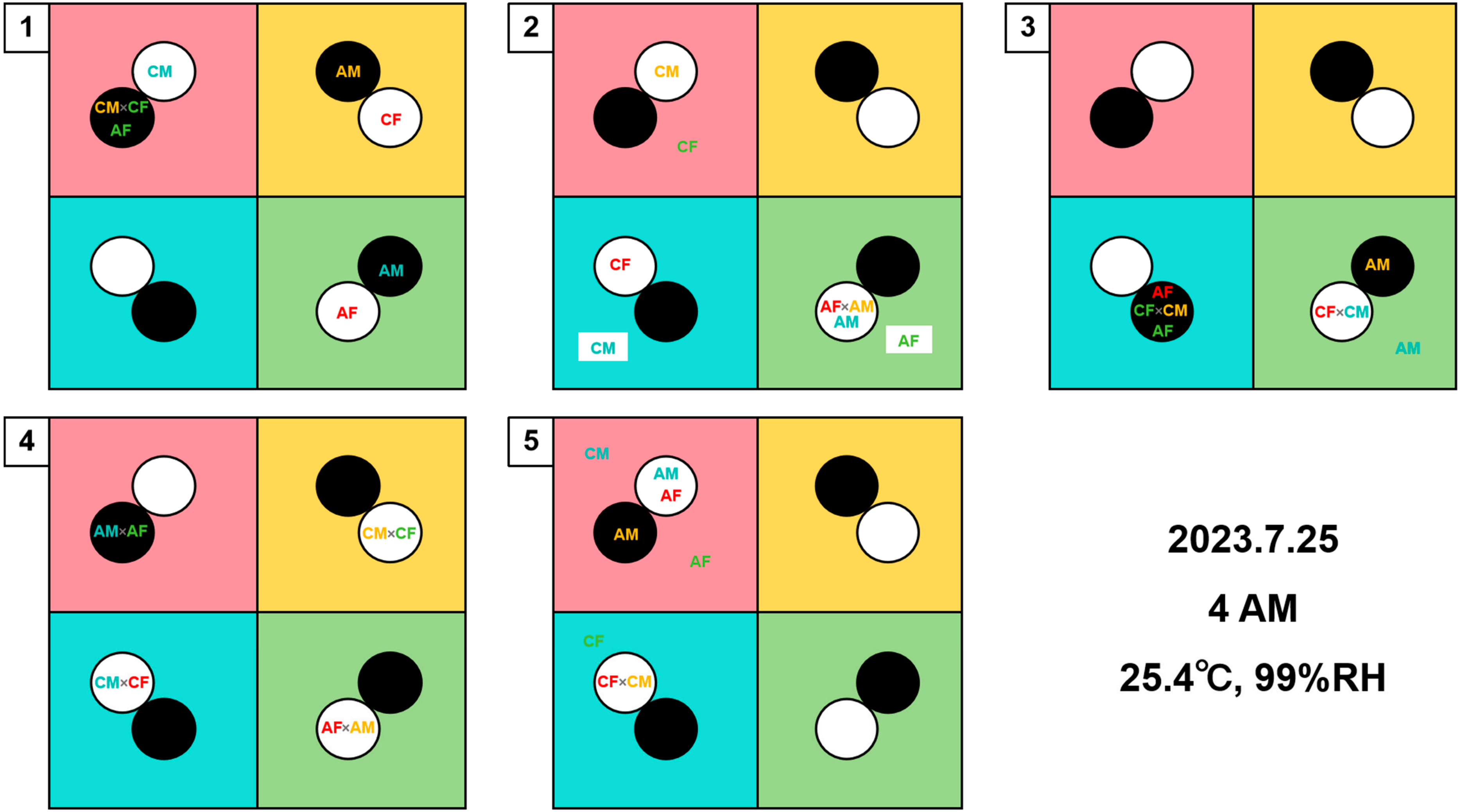

2023.7.25

4 AM

25.4℃, 99%RH

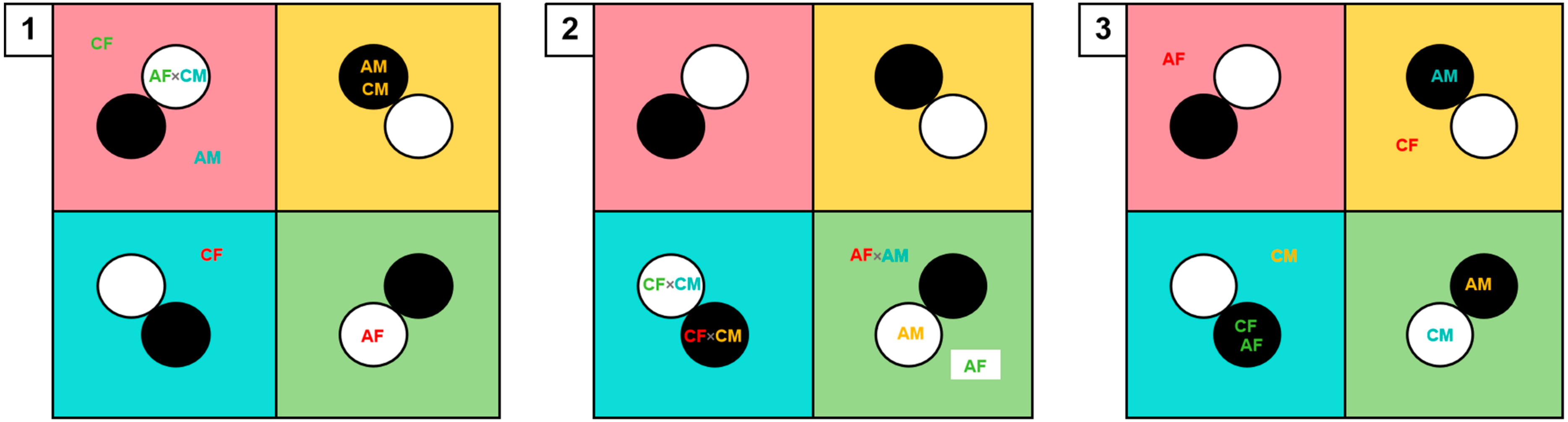

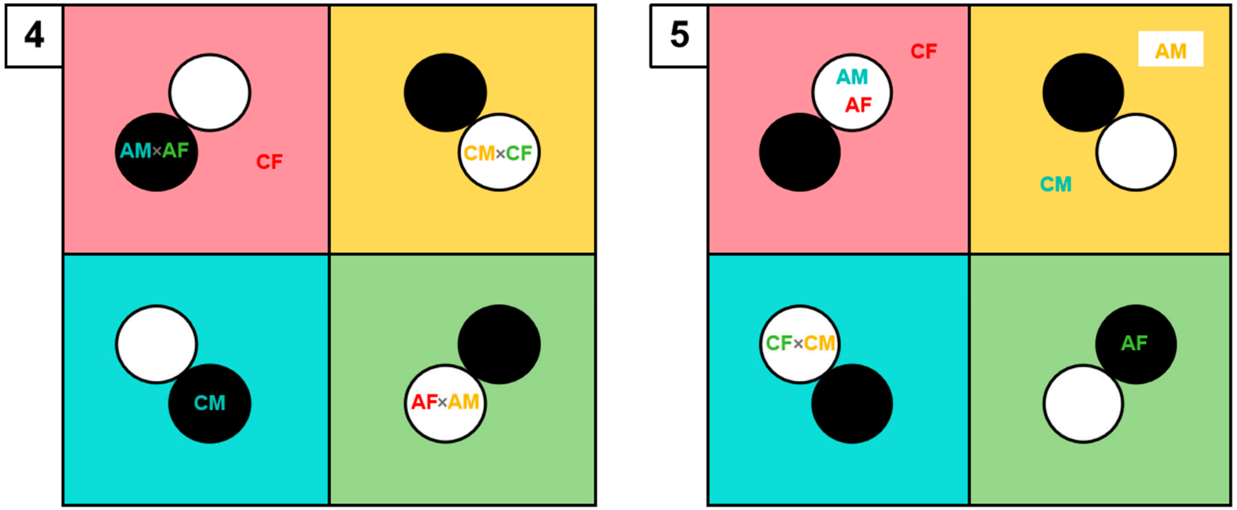

2023.7.25

6 AM

25.4℃, 99%RH

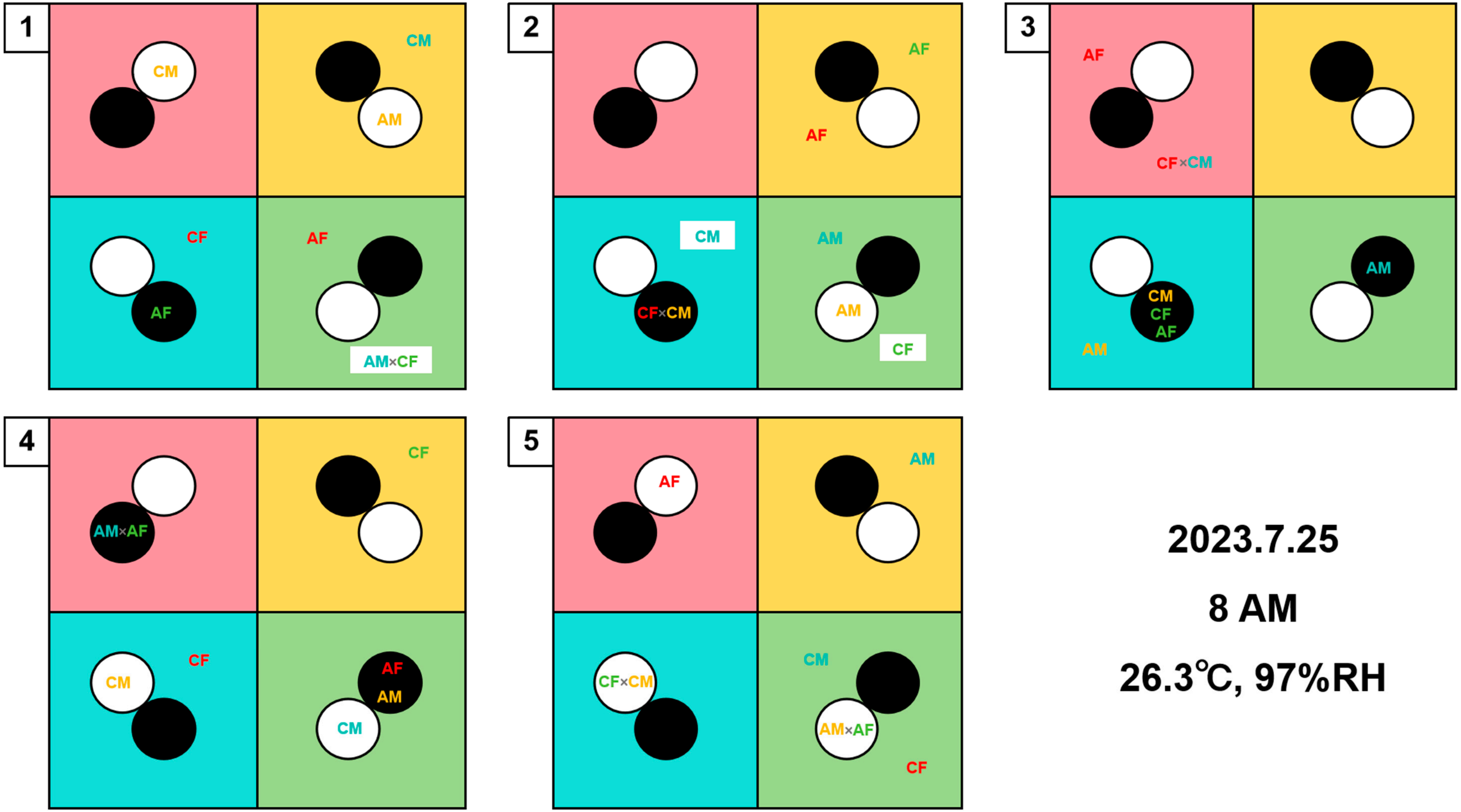

2023.7.25

8 AM

26.3℃, 97%RH

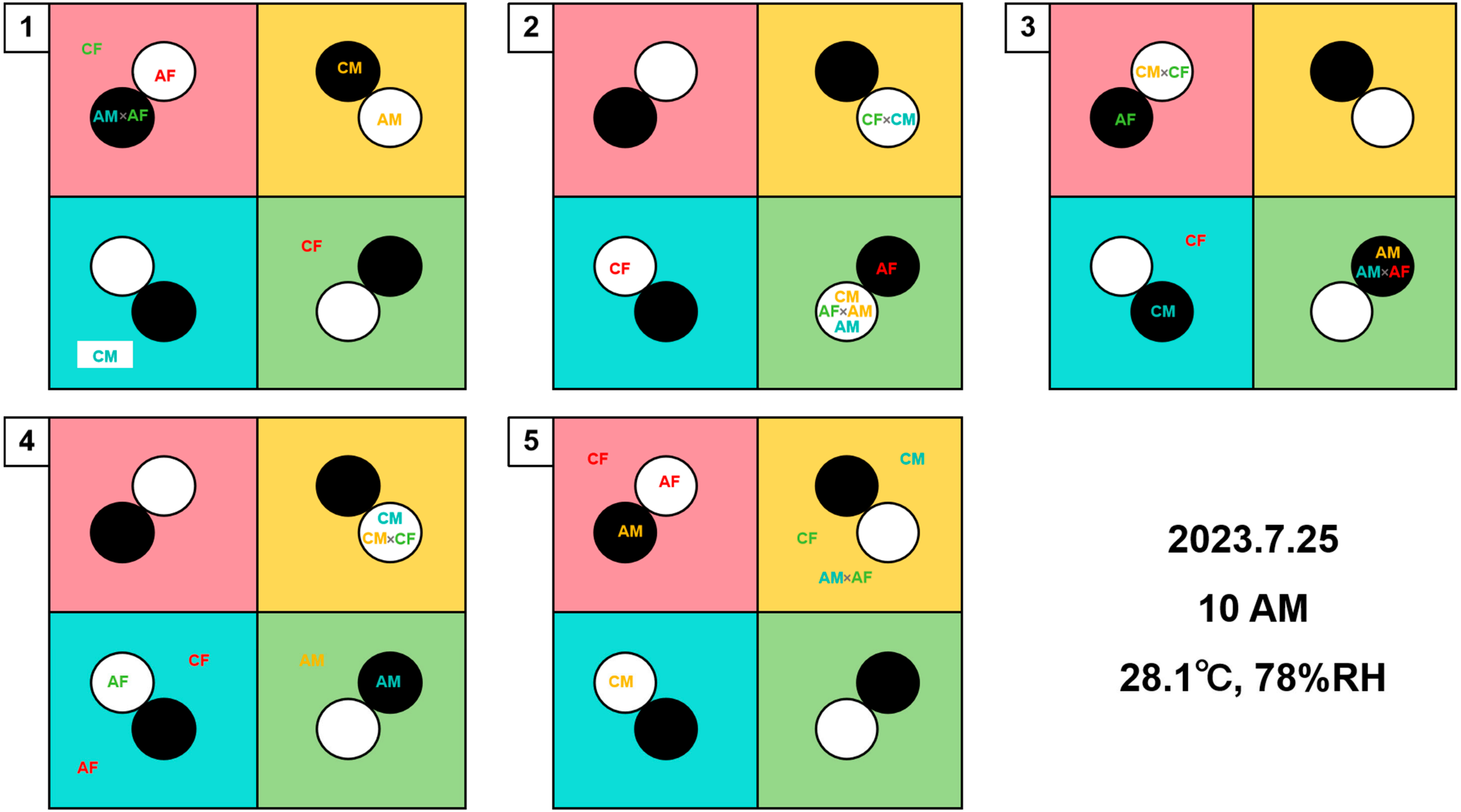

2023.7.25

10 AM

28.1℃, 78%RH

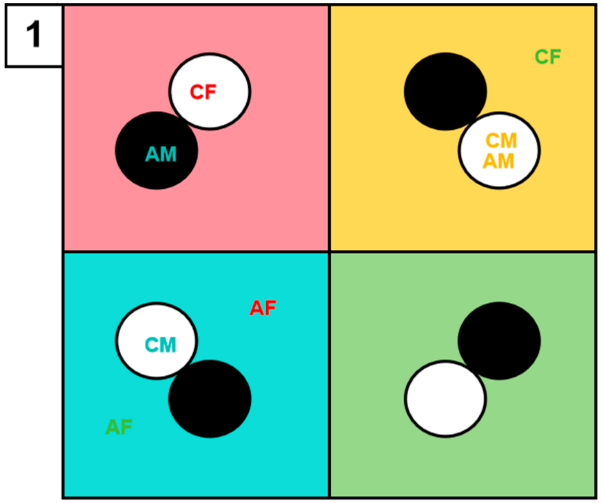

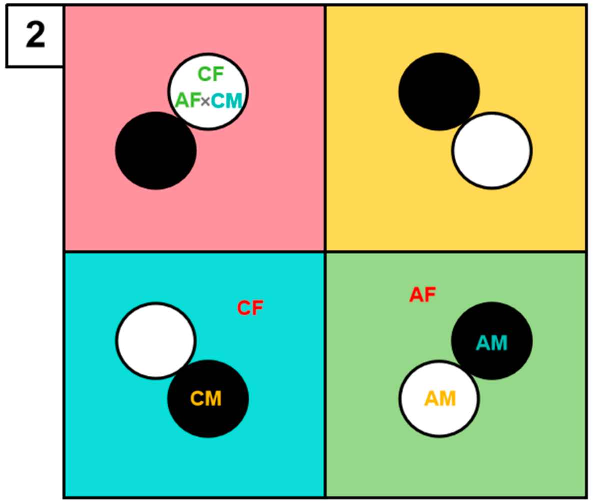

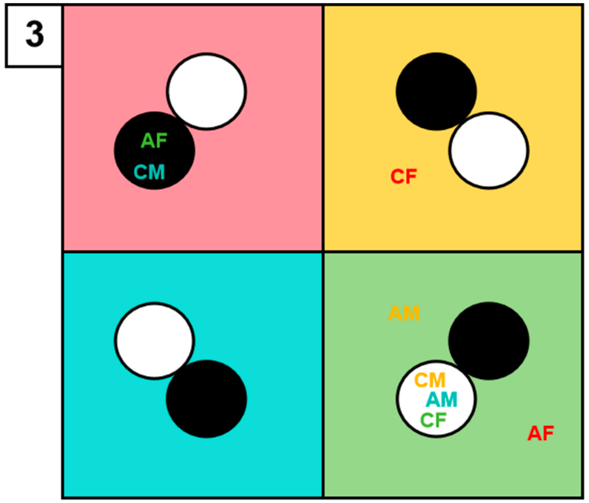

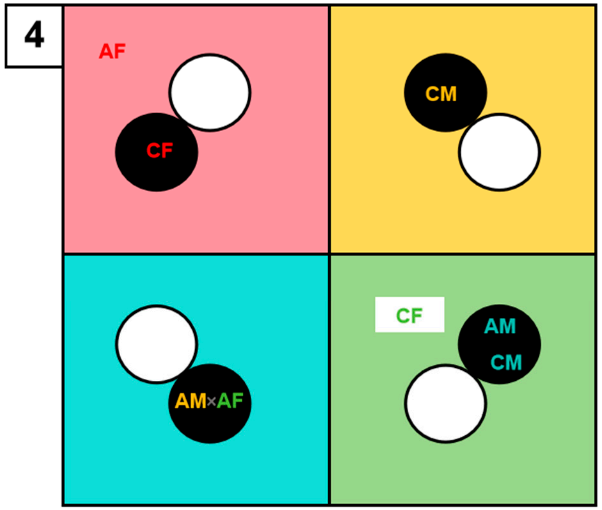

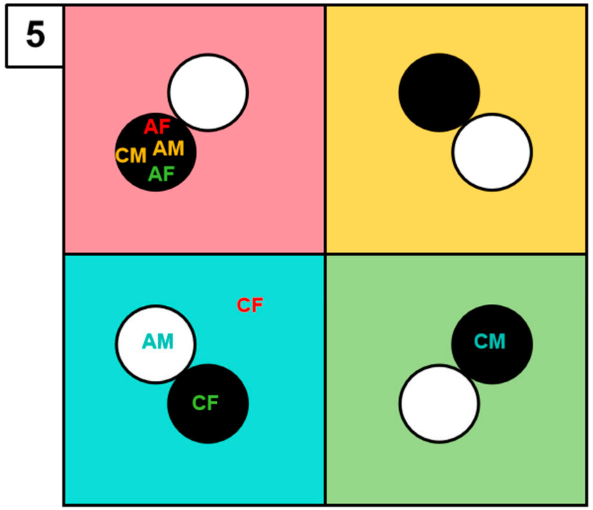

2023.7.25

12 PM

29.4℃, 68%RH

Figure S2: the positions and mounting pairs of beetles observed in the cages at the time points from August 1 to August 3 2023

In figure:

AF: female *Anoplophora glabripennis*

AM: male *Anoplophora glabripennis*

CF: female *Anoplophora chinensis*

CM: male *Anoplophora chinensis*

"×": mounting

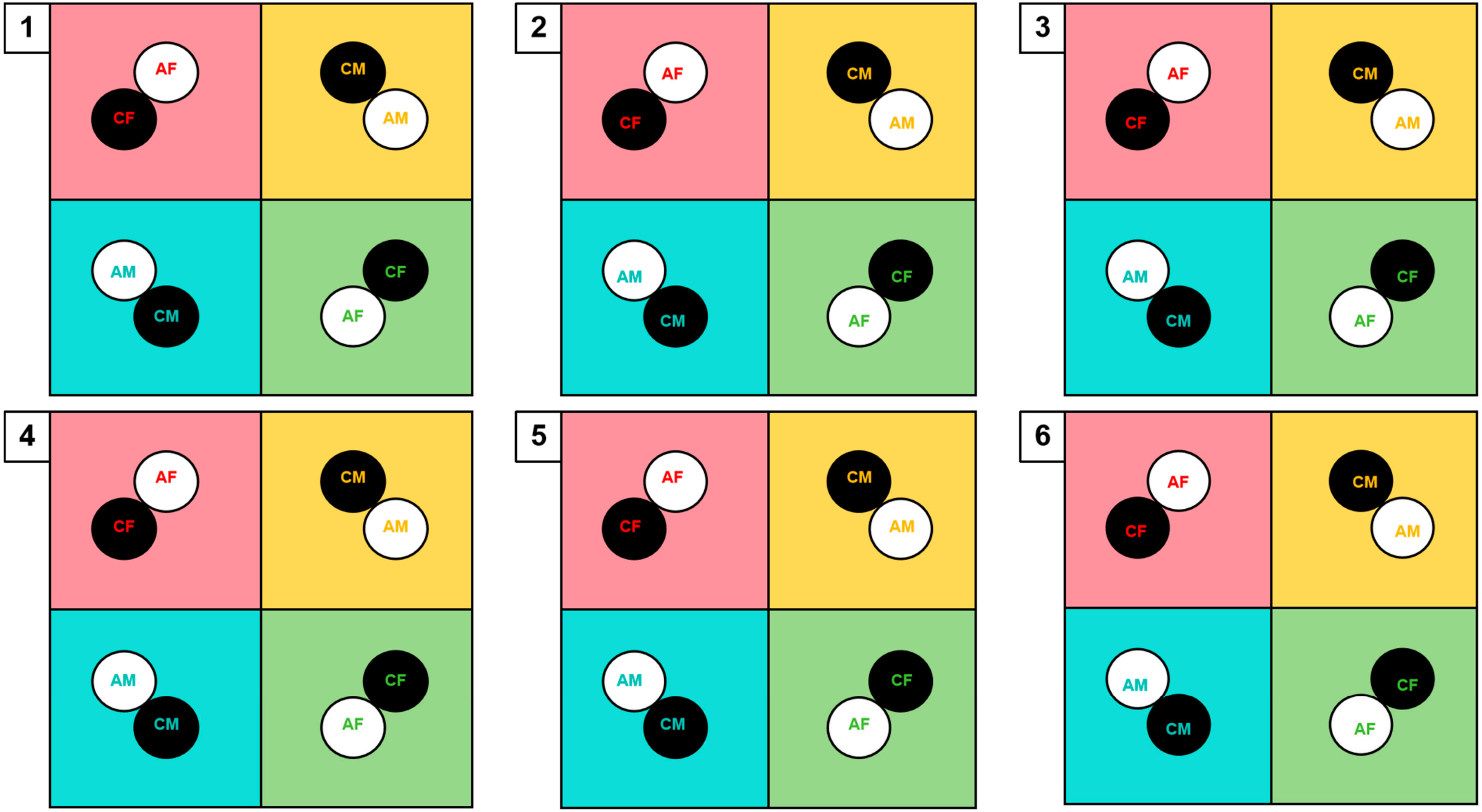

2023.8.1, 12 PM, Release point

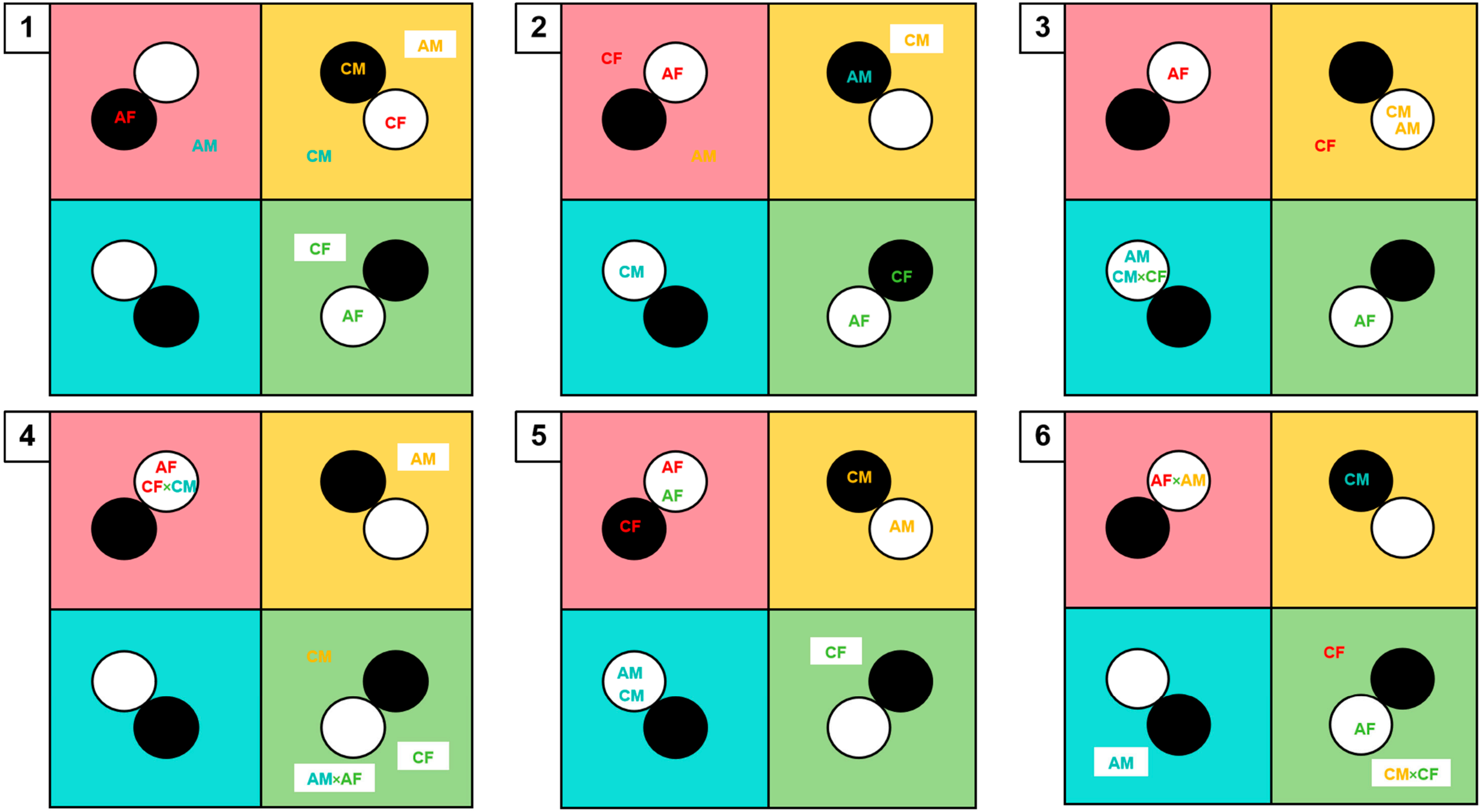

2023.8.1, 2 PM, 28.4℃, 55%RH

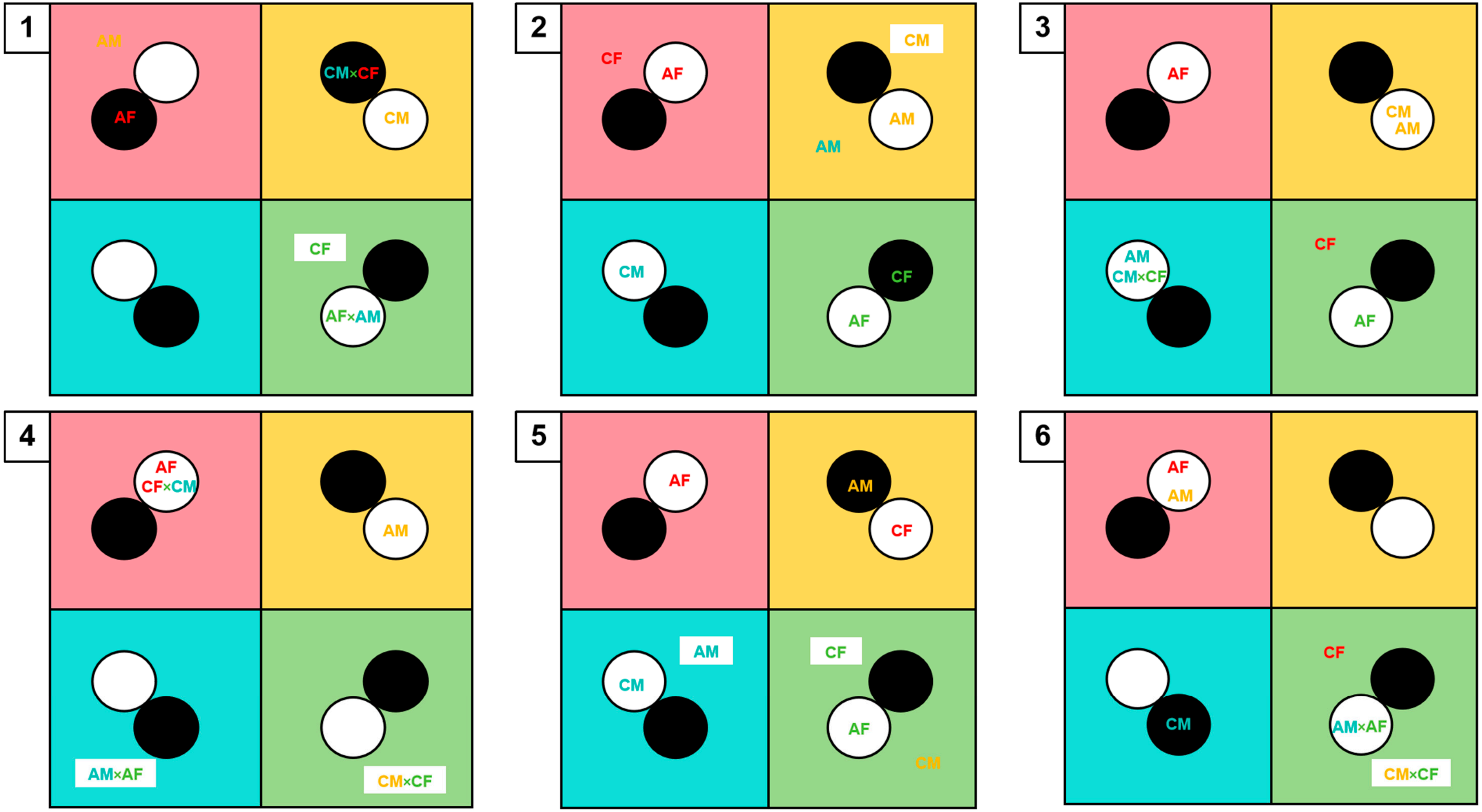

2023.8.1, 4 PM, 29.1℃, 51%RH

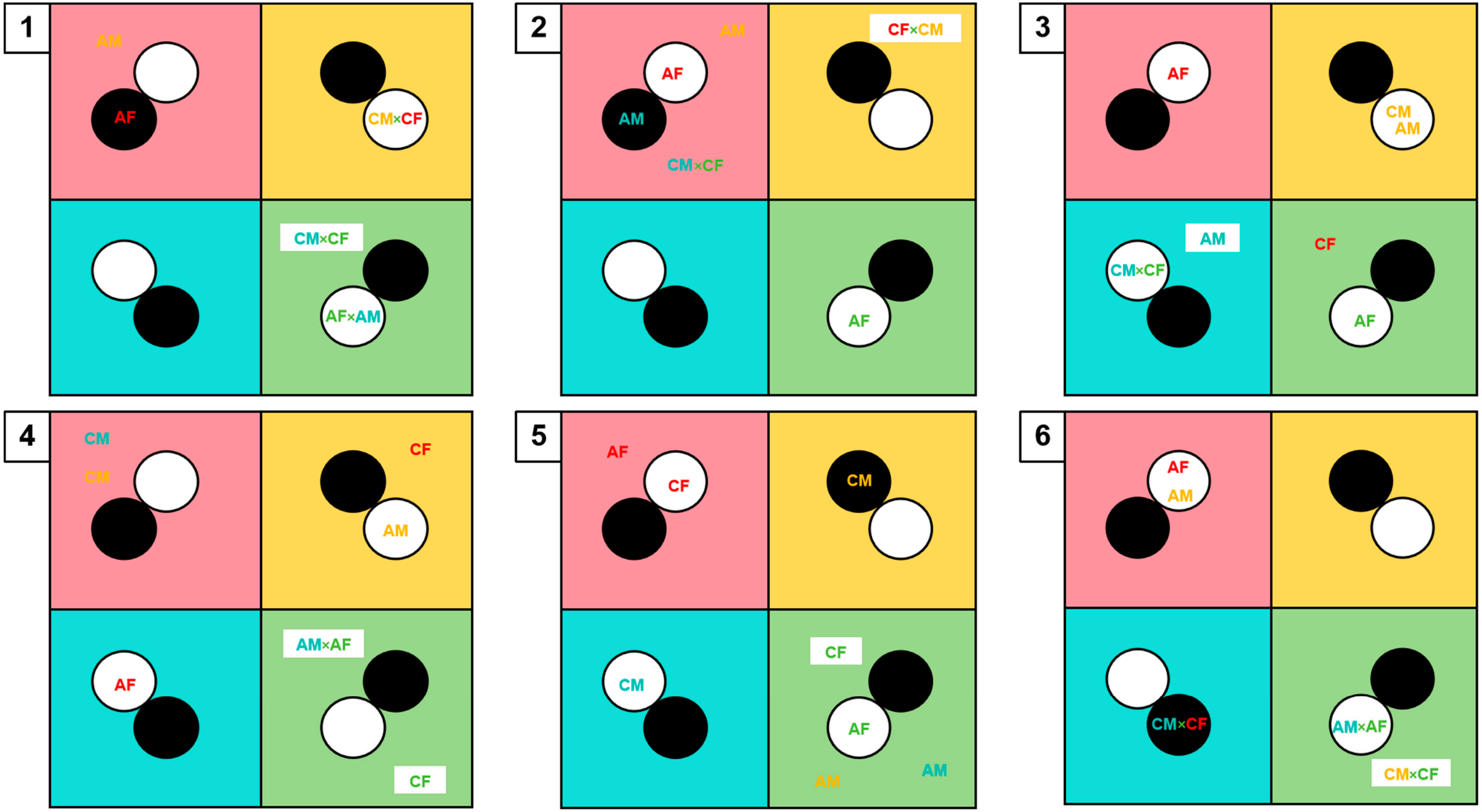

2023.8.1, 6 PM, 27.5℃, 62%RH

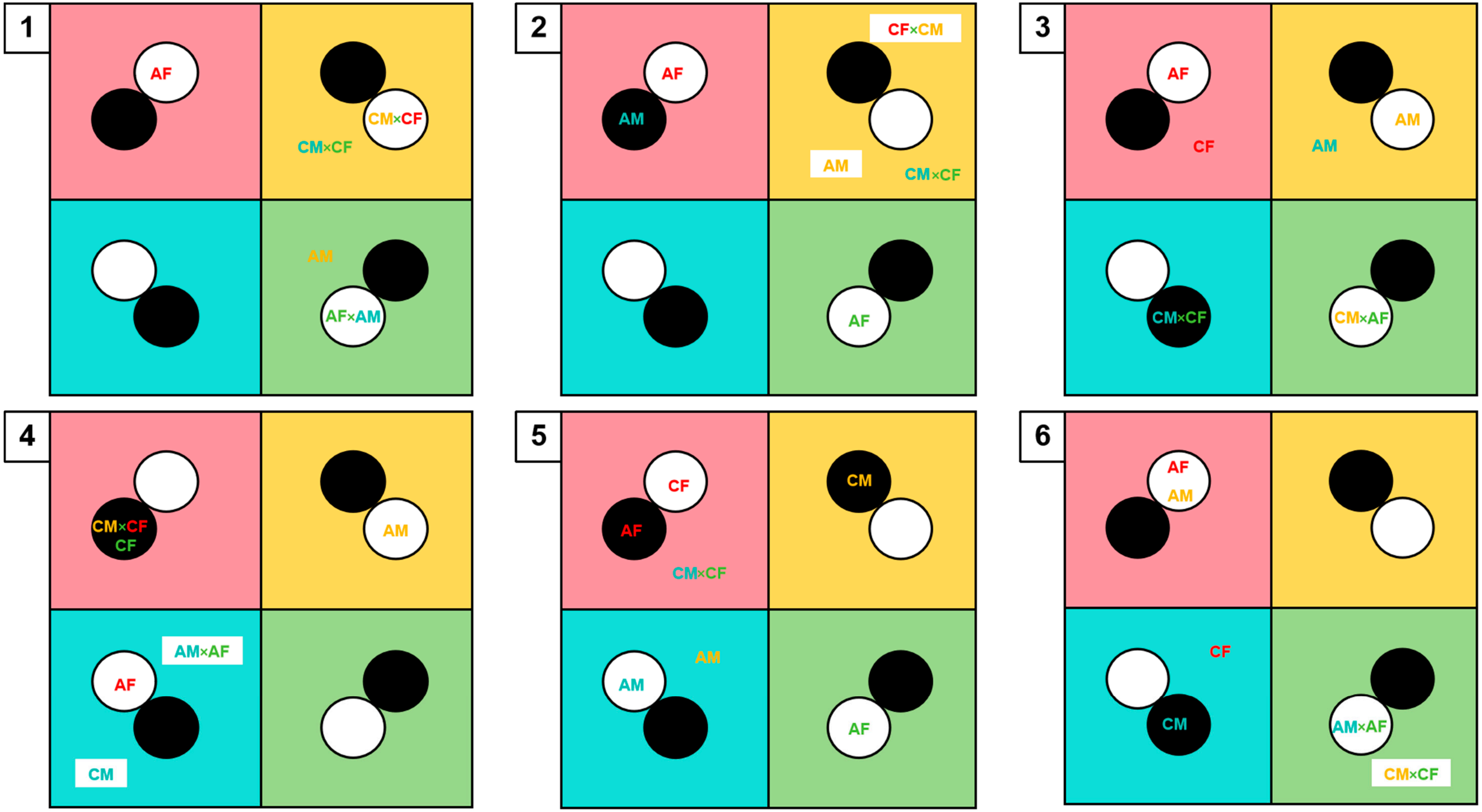

2023.8.1, 8 PM, 26.7℃, 73%RH

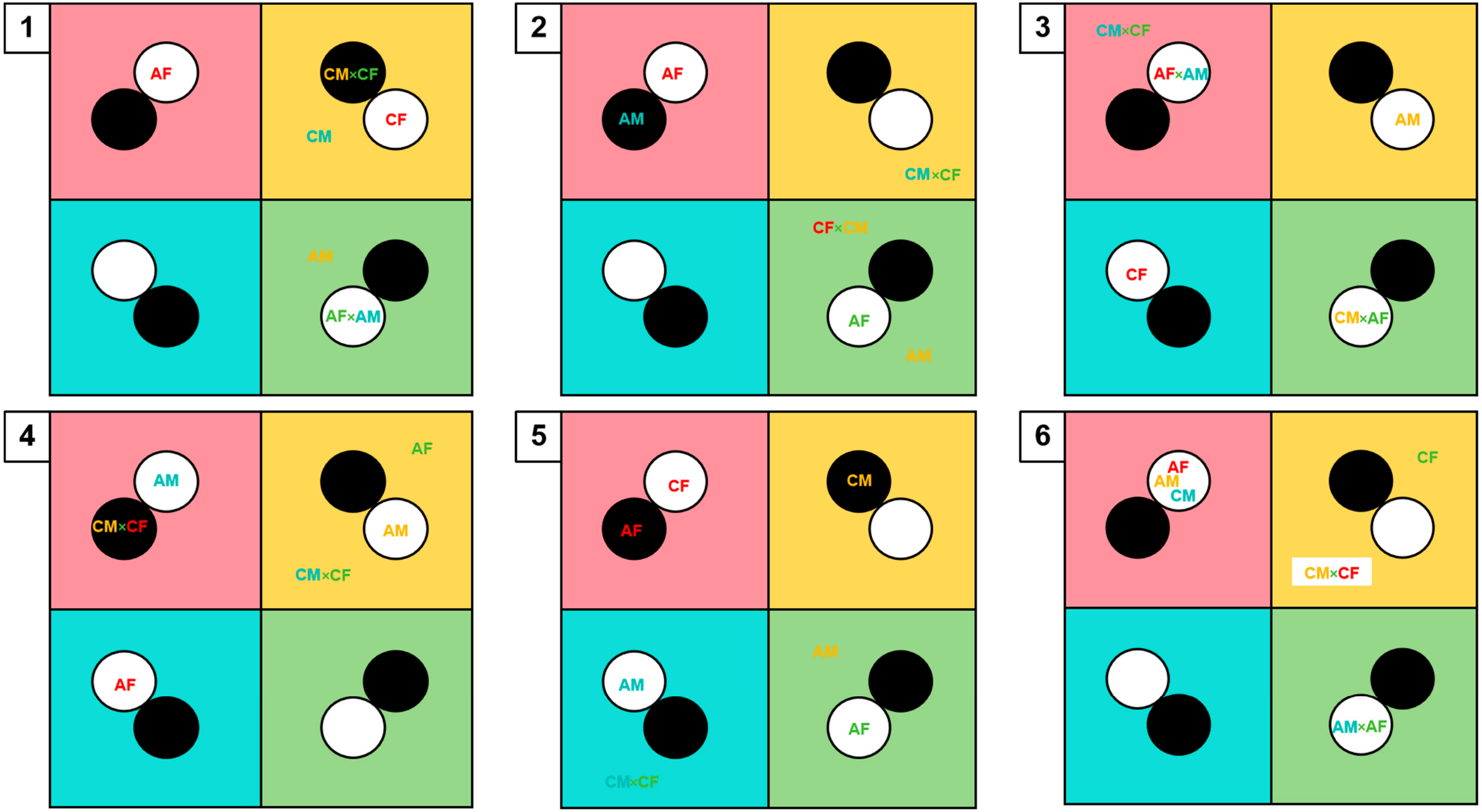

2023.8.1, 10 PM, 26.1℃, 82%RH

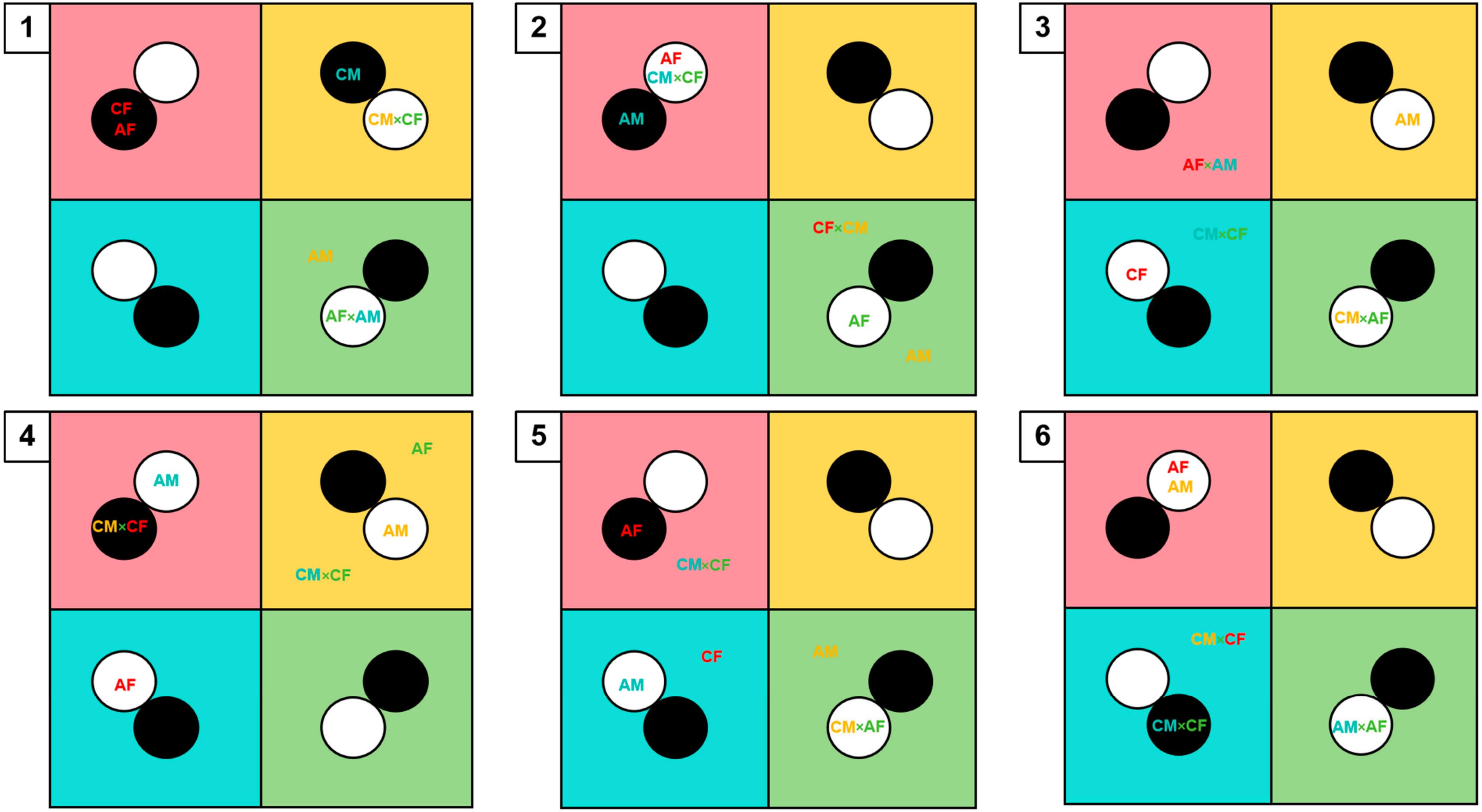

2023.8.2, 12 AM, 26℃, 85%RH

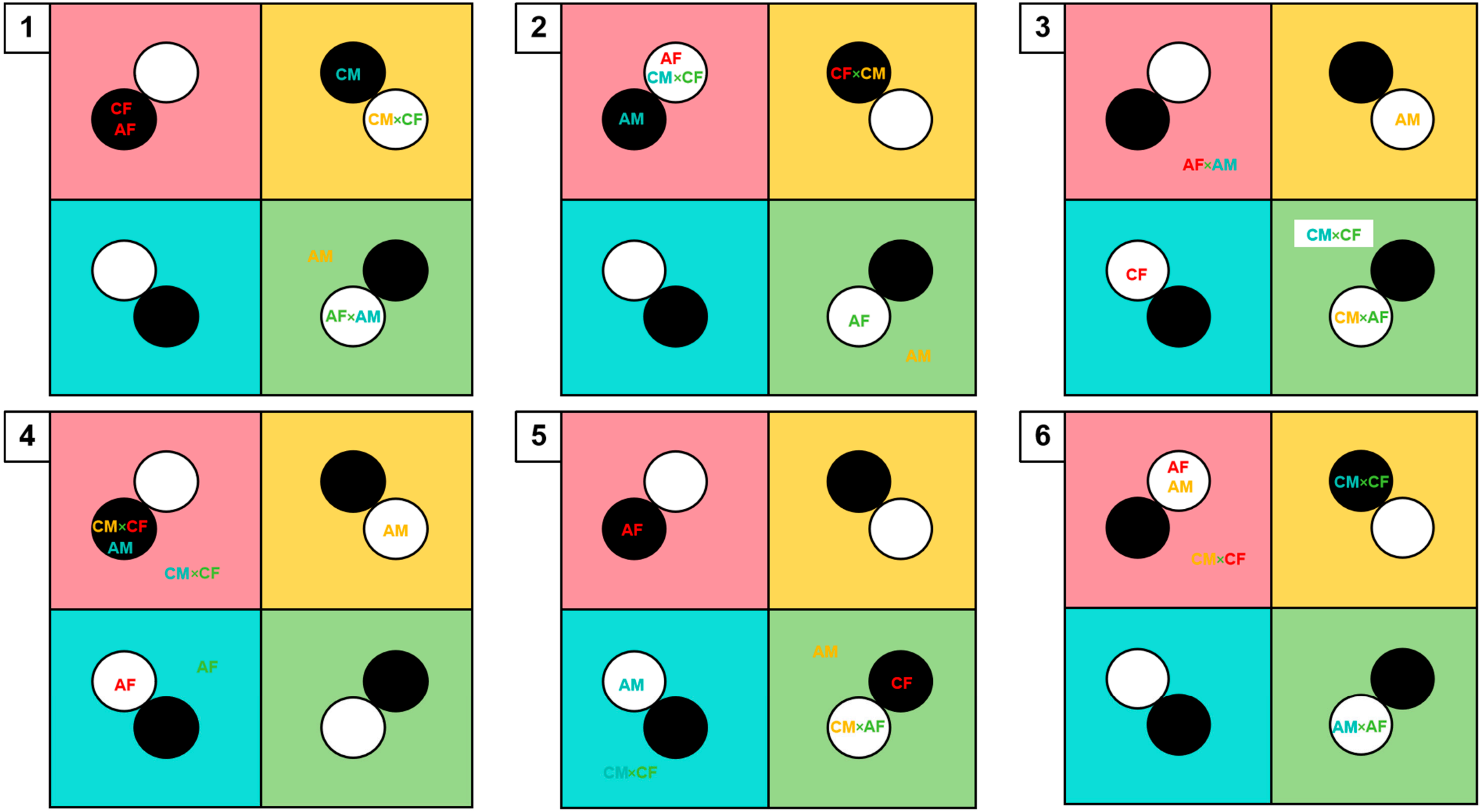

2023.8.2, 2 AM, 26.2℃, 74%RH

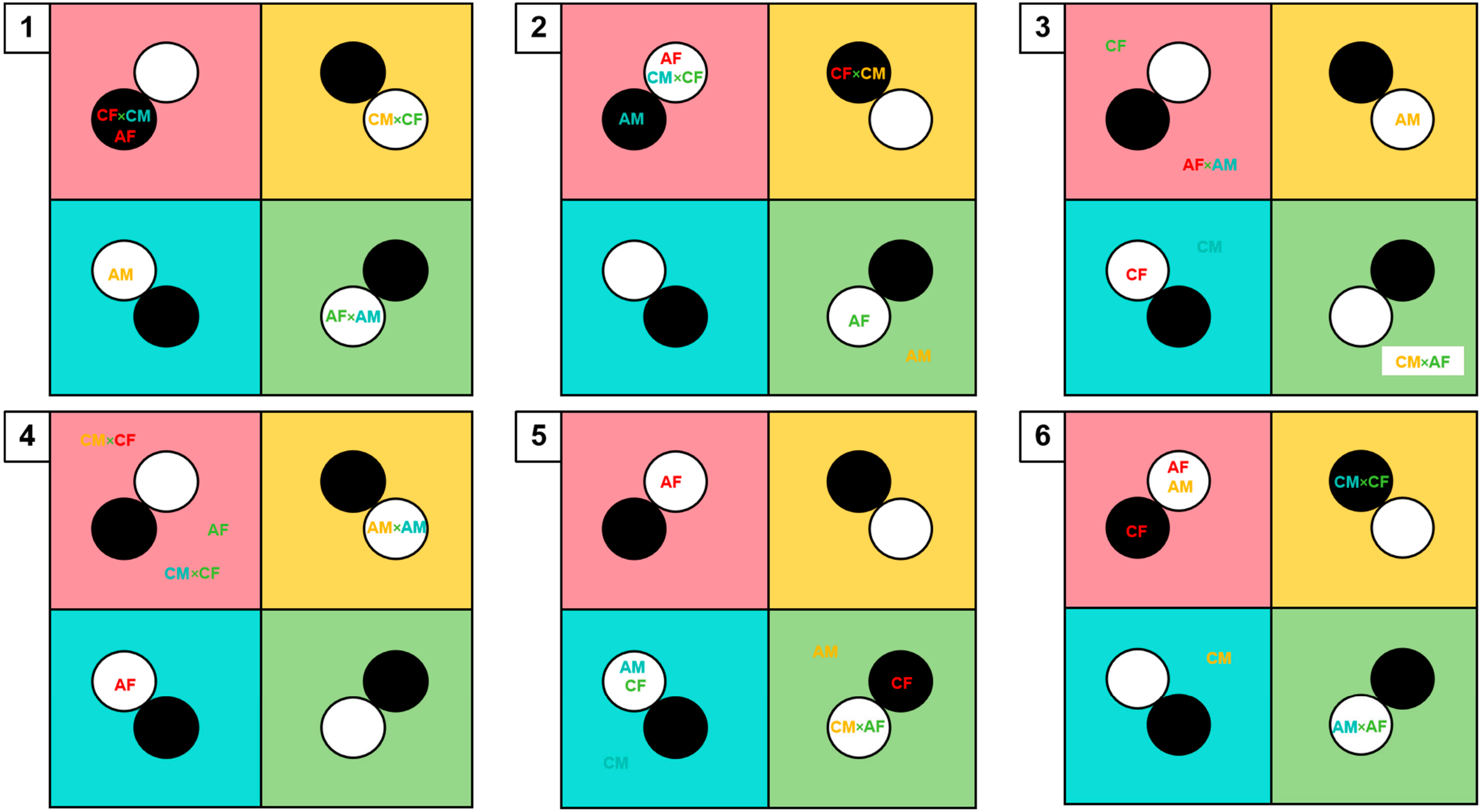

2023.8.2, 4 AM, 25.8℃, 80%RH

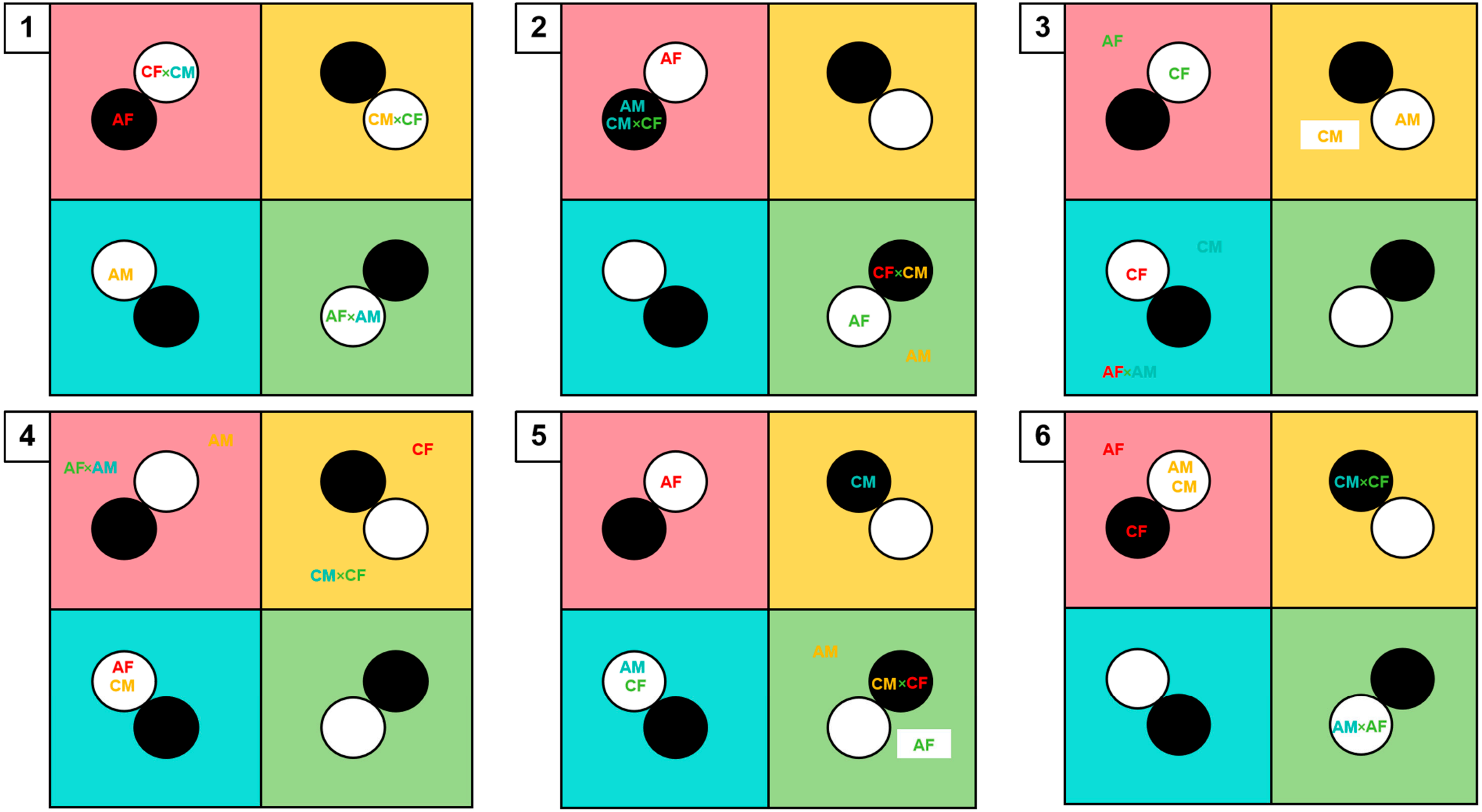

2023.8.2, 6 AM, 25.5℃, 90%RH

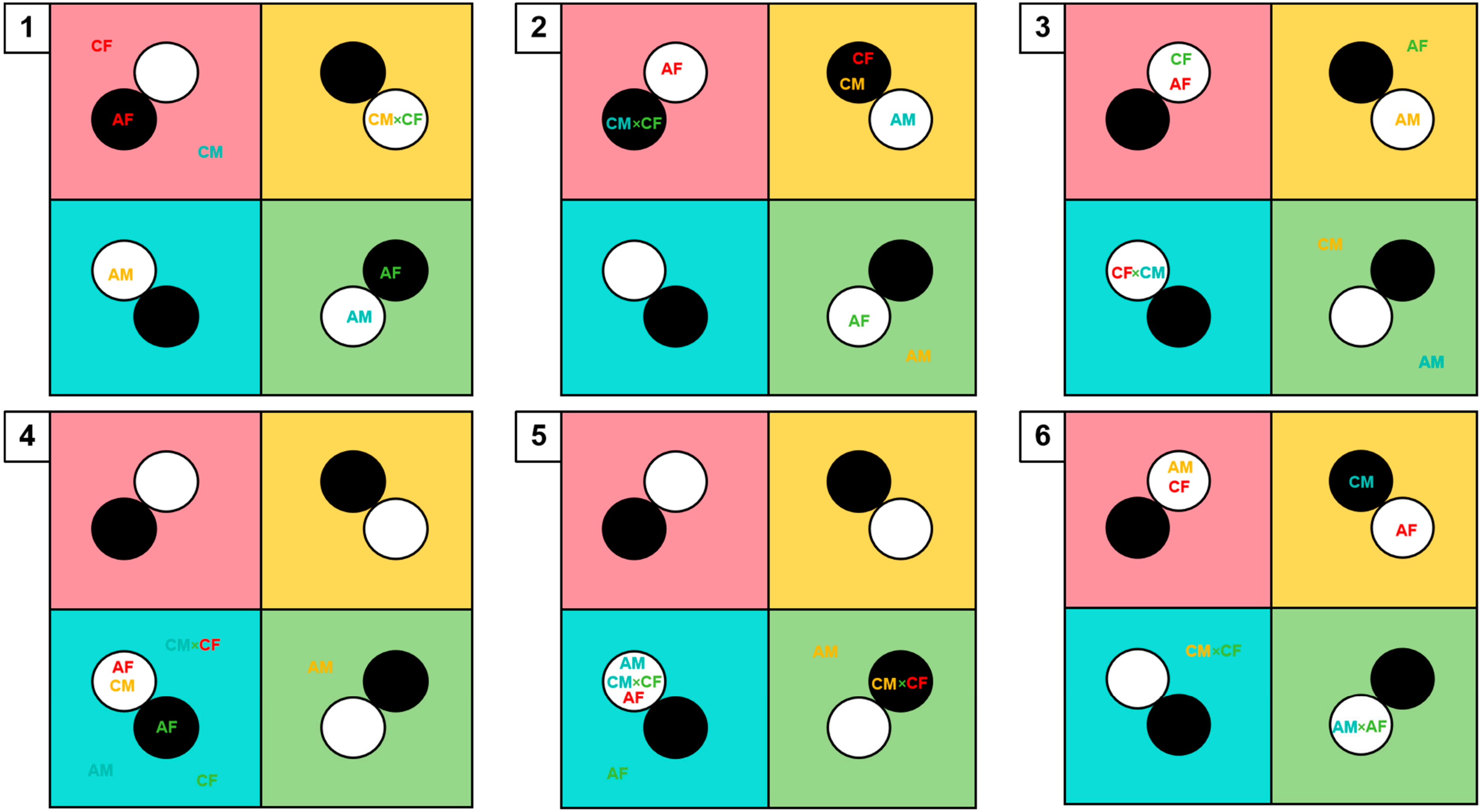

2023.8.2, 8 AM, 26.5℃, 82%RH

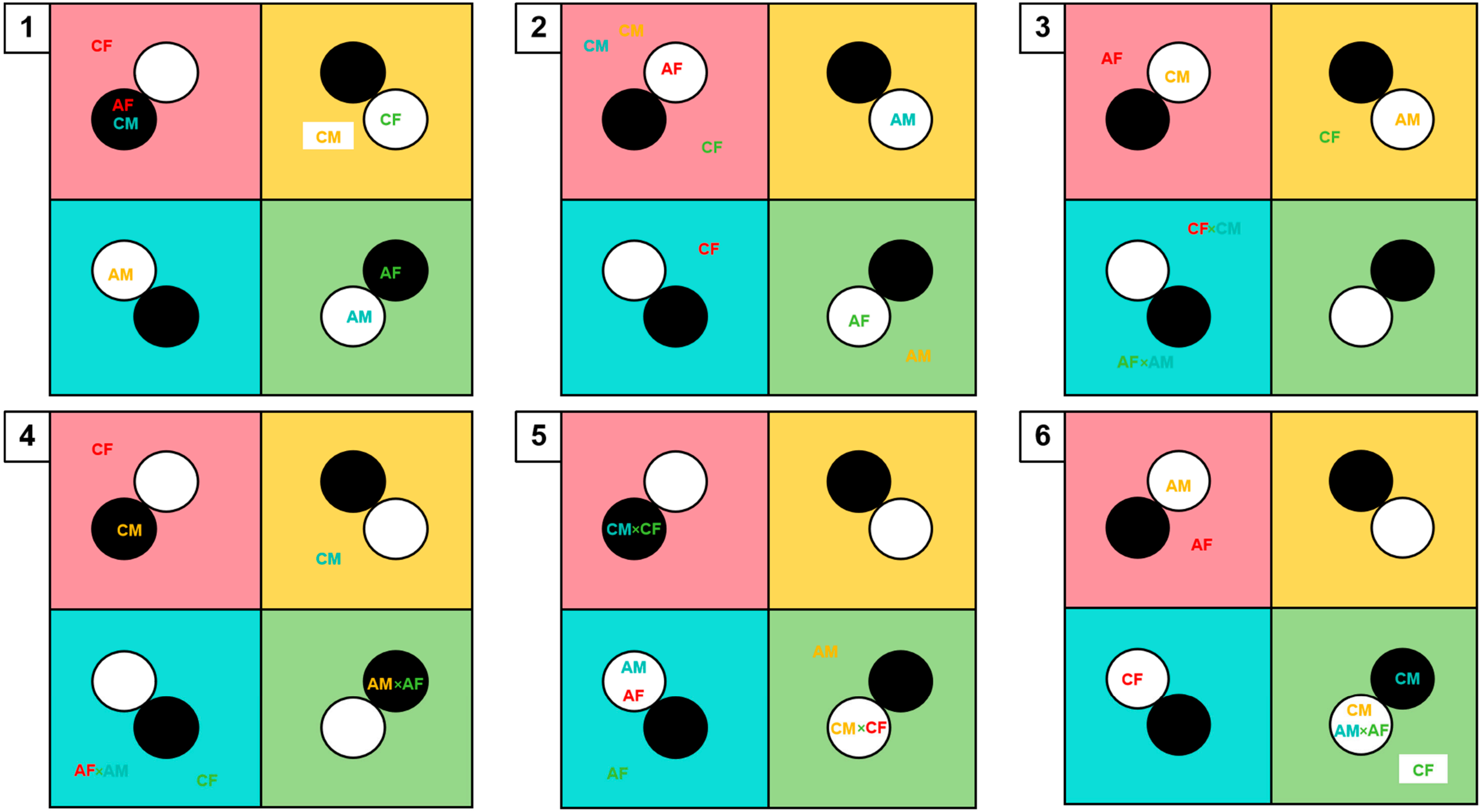

2023.8.2, 10 AM, 29.4℃, 57%RH

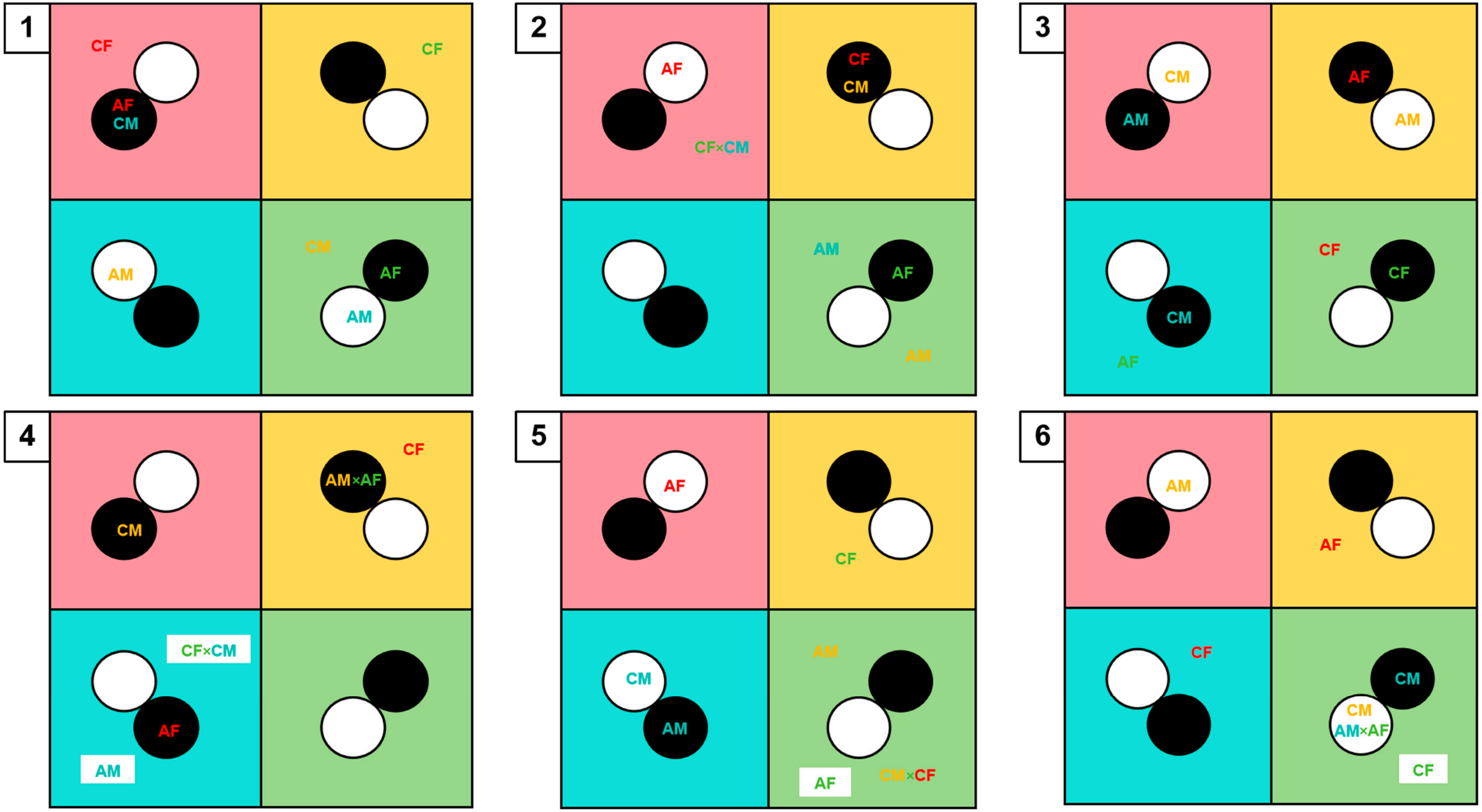

2023.8.2, 12 PM, 29.5℃, 56%RH

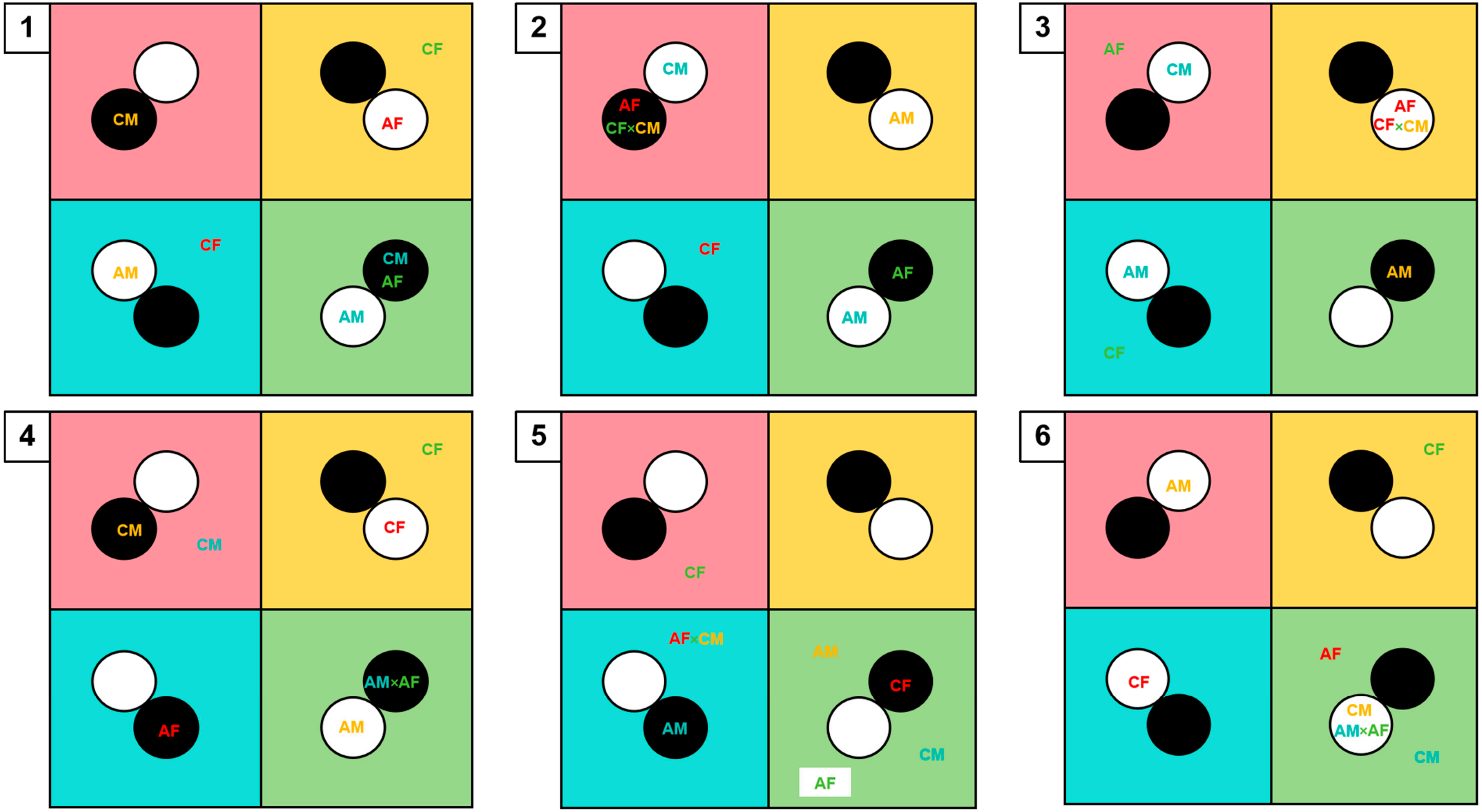

2023.8.2, 2 PM, 30.1℃, 51%RH

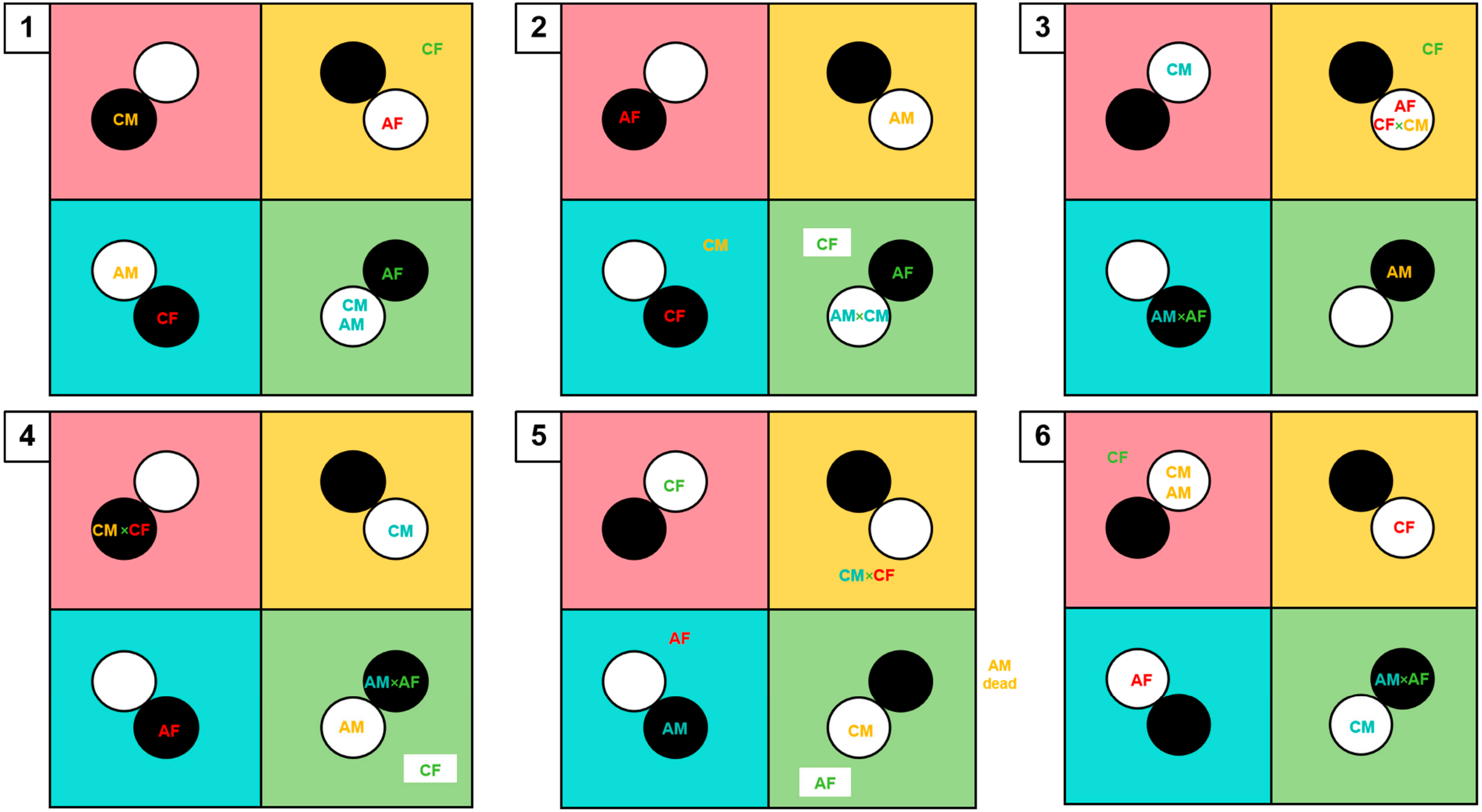

2023.8.2, 4 PM, 30℃, 54%RH

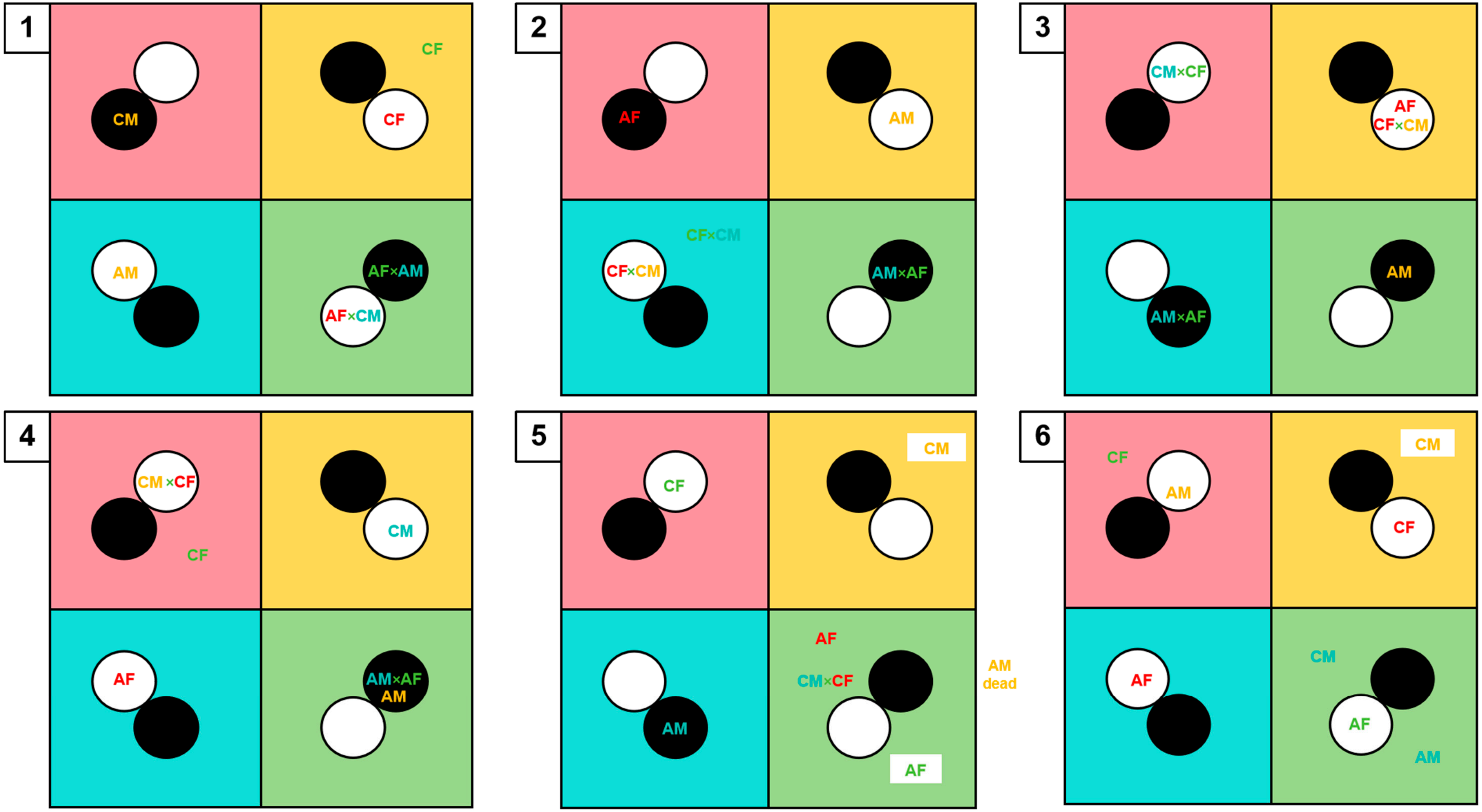

2023.8.2, 6 PM, 28.7℃, 63%RH

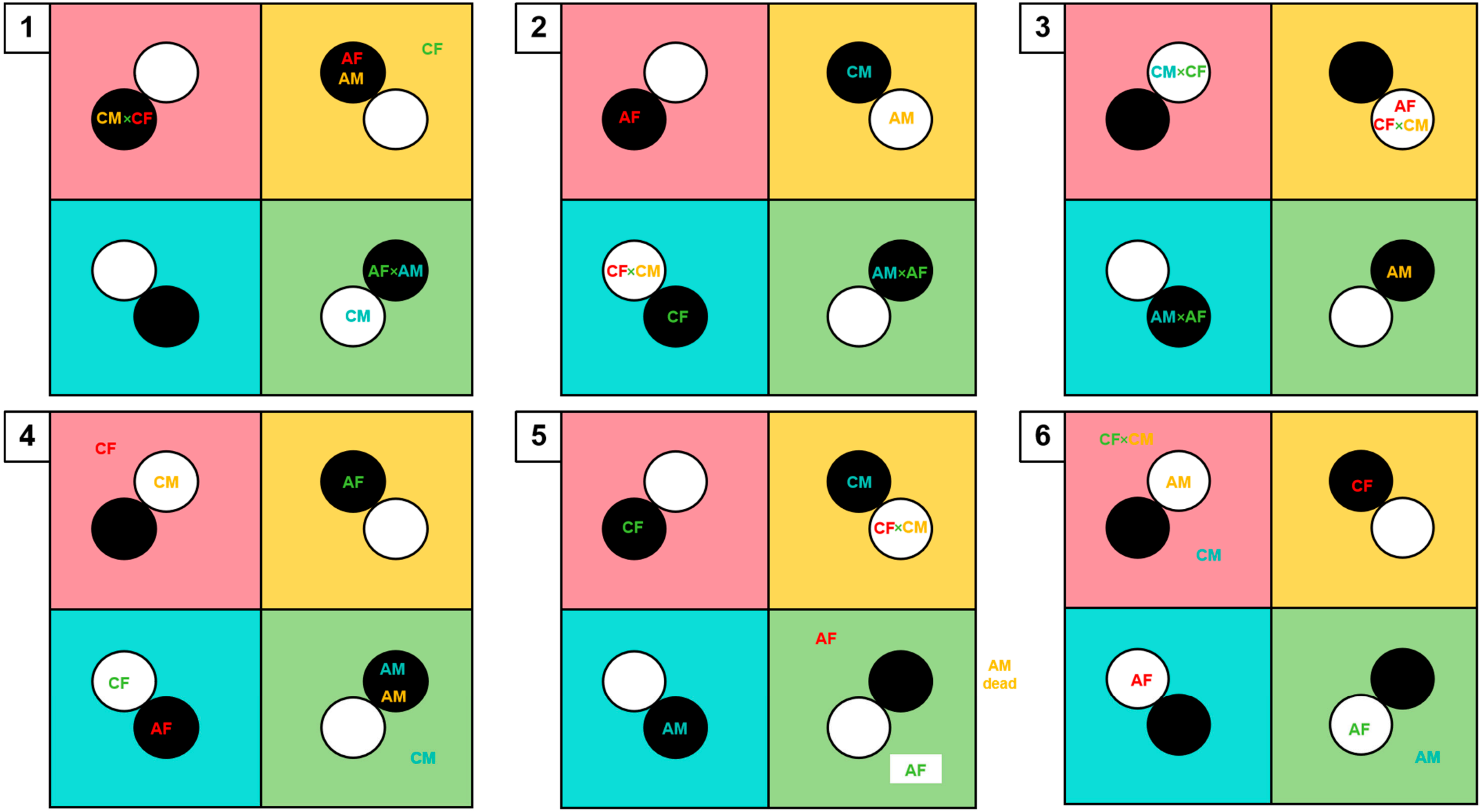

2023.8.2, 8 PM, 28.1℃, 65%RH

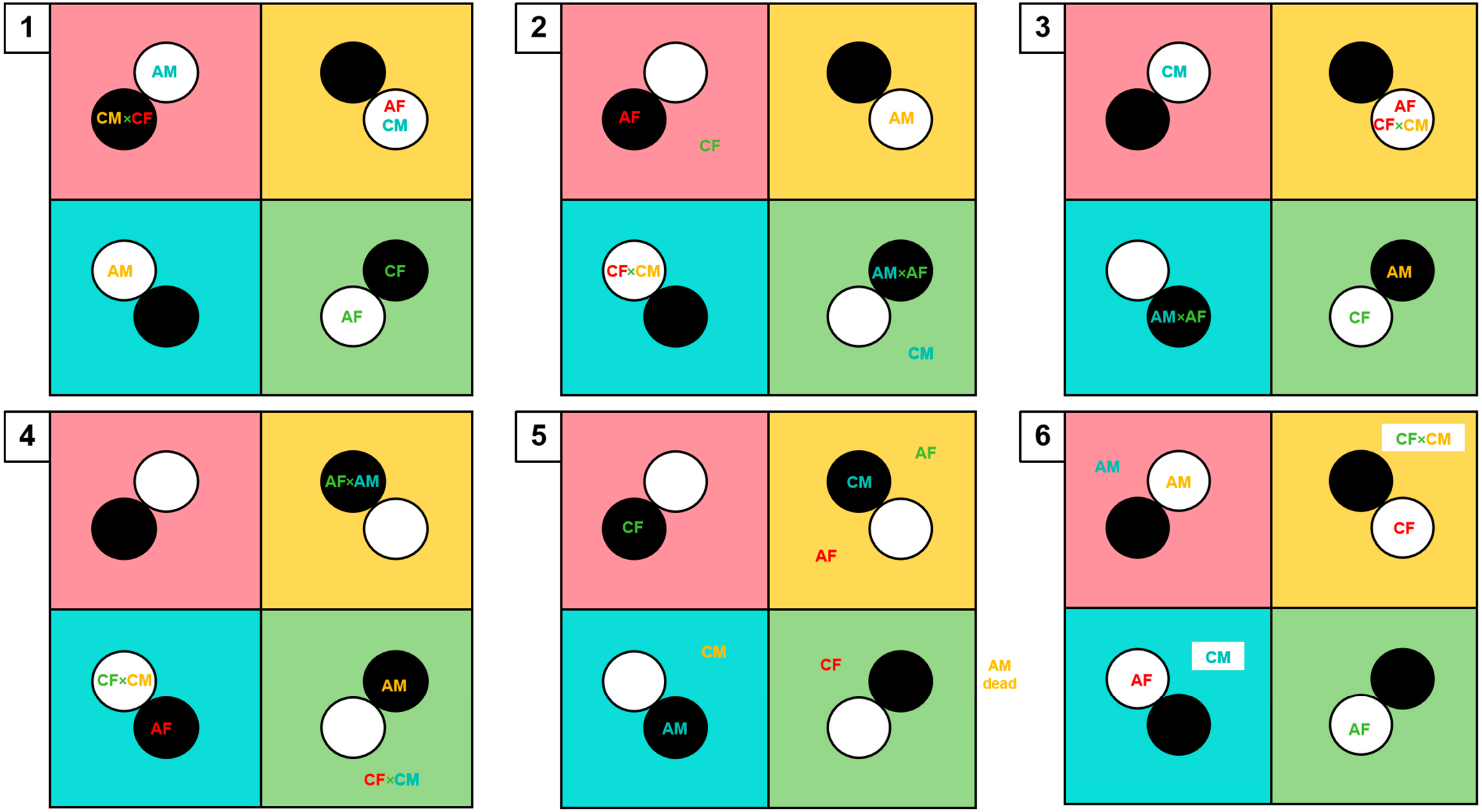

2023.8.2, 10 PM, 27.6℃, 67%RH

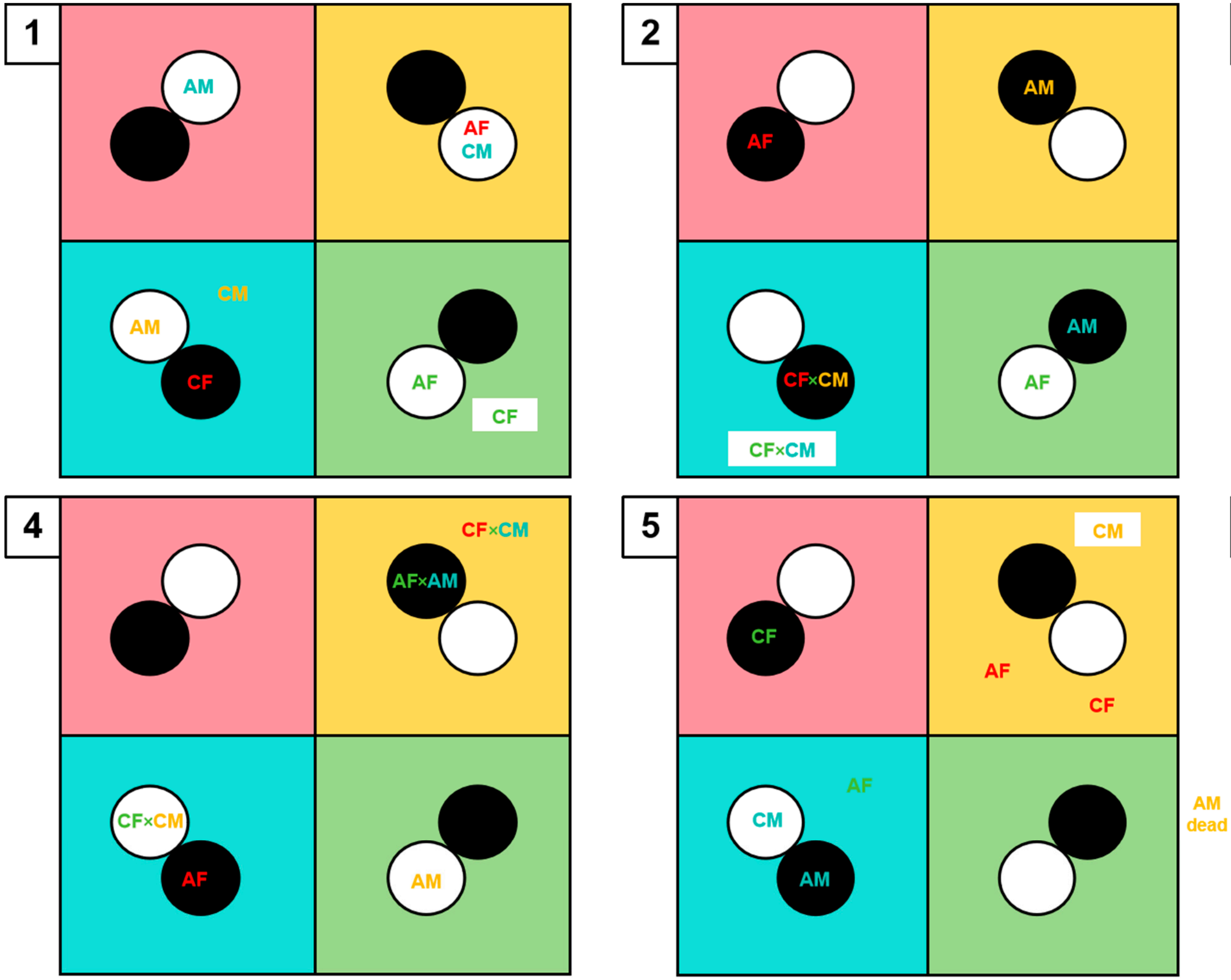

2023.8.3, 12 AM, 27℃, 73%RH

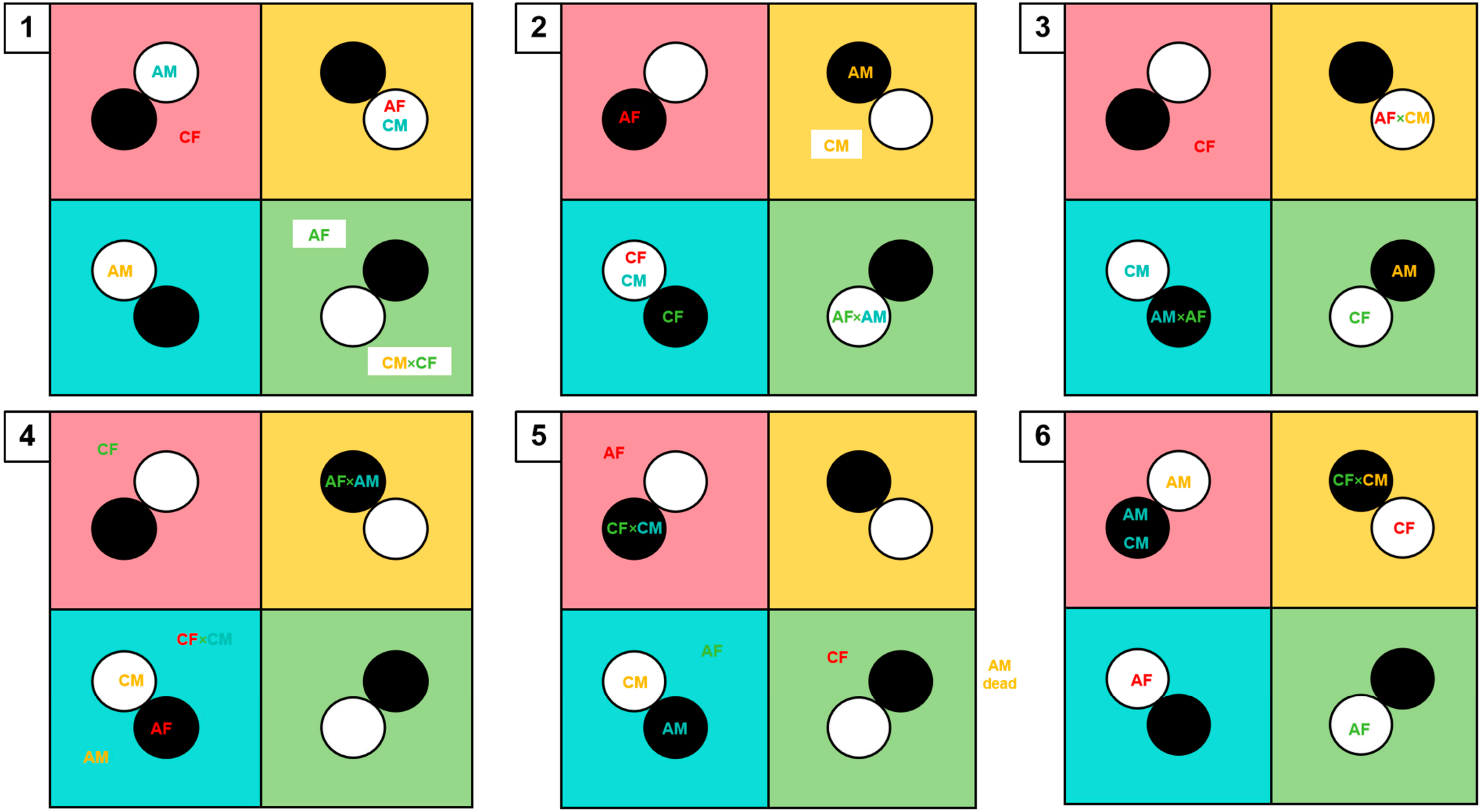

2023.8.3, 2 AM, 26.4℃, 84%RH

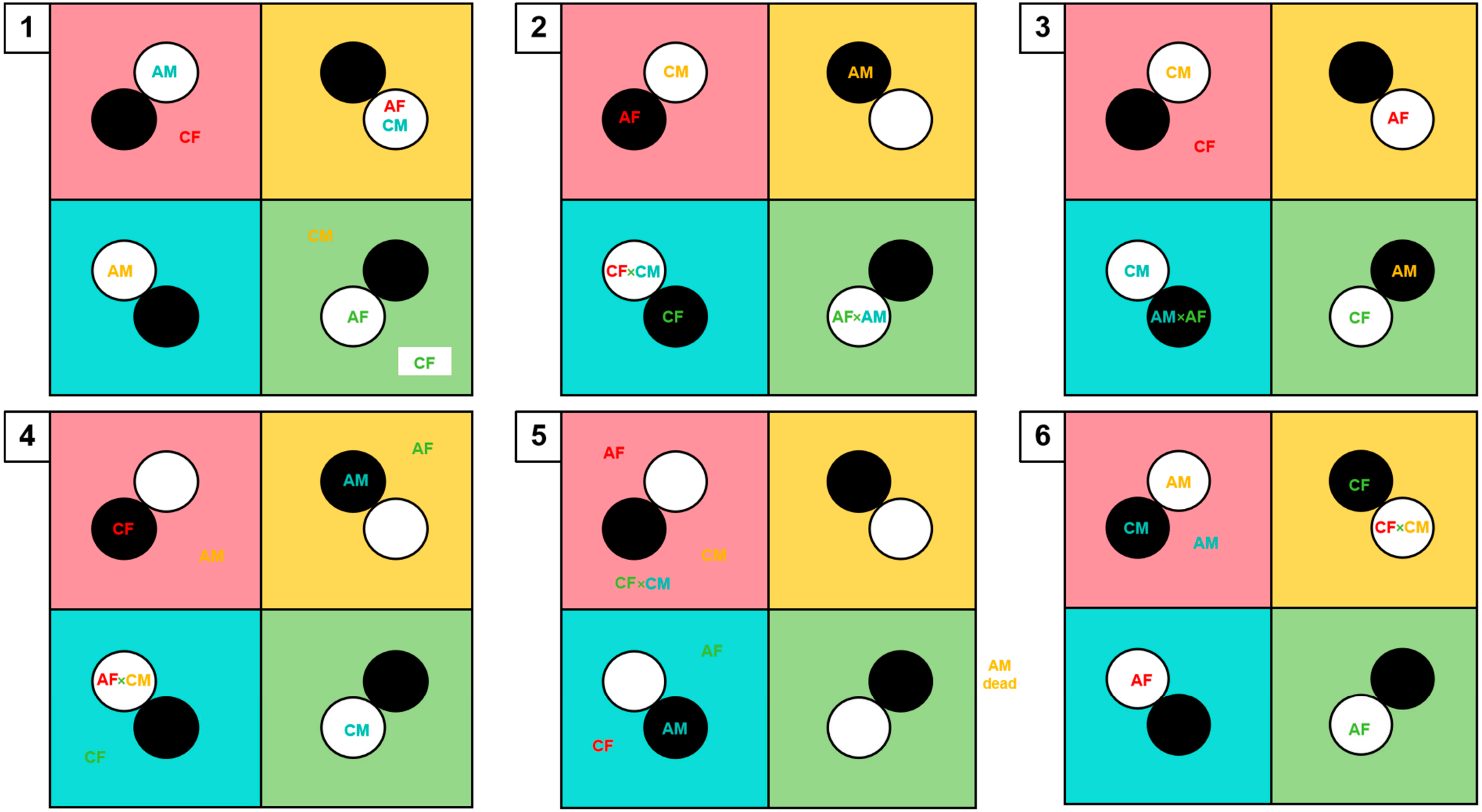

2023.8.3, 4 AM, 26℃, 87%RH

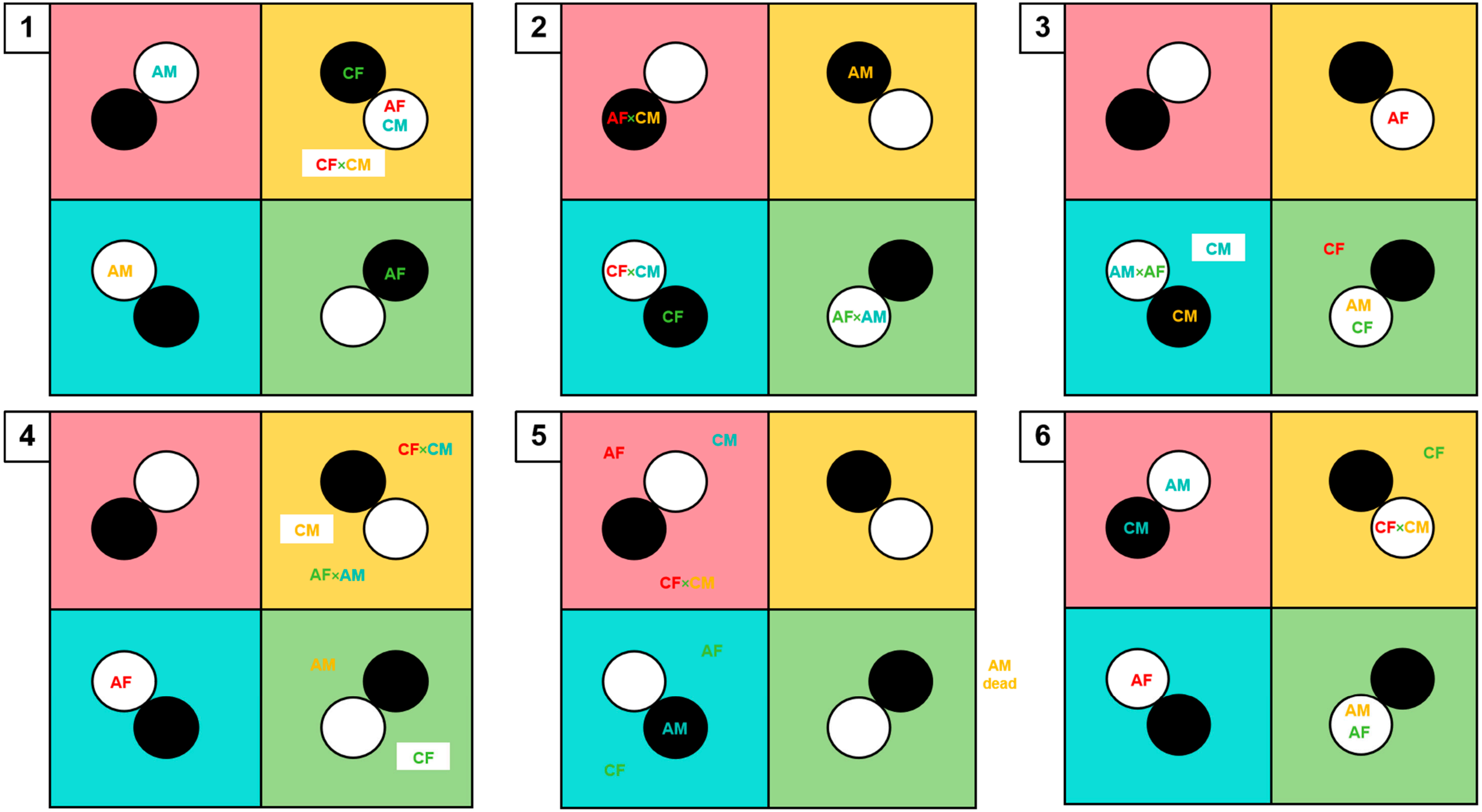

2023.8.3, 6 AM, 25.7℃, 88%RH

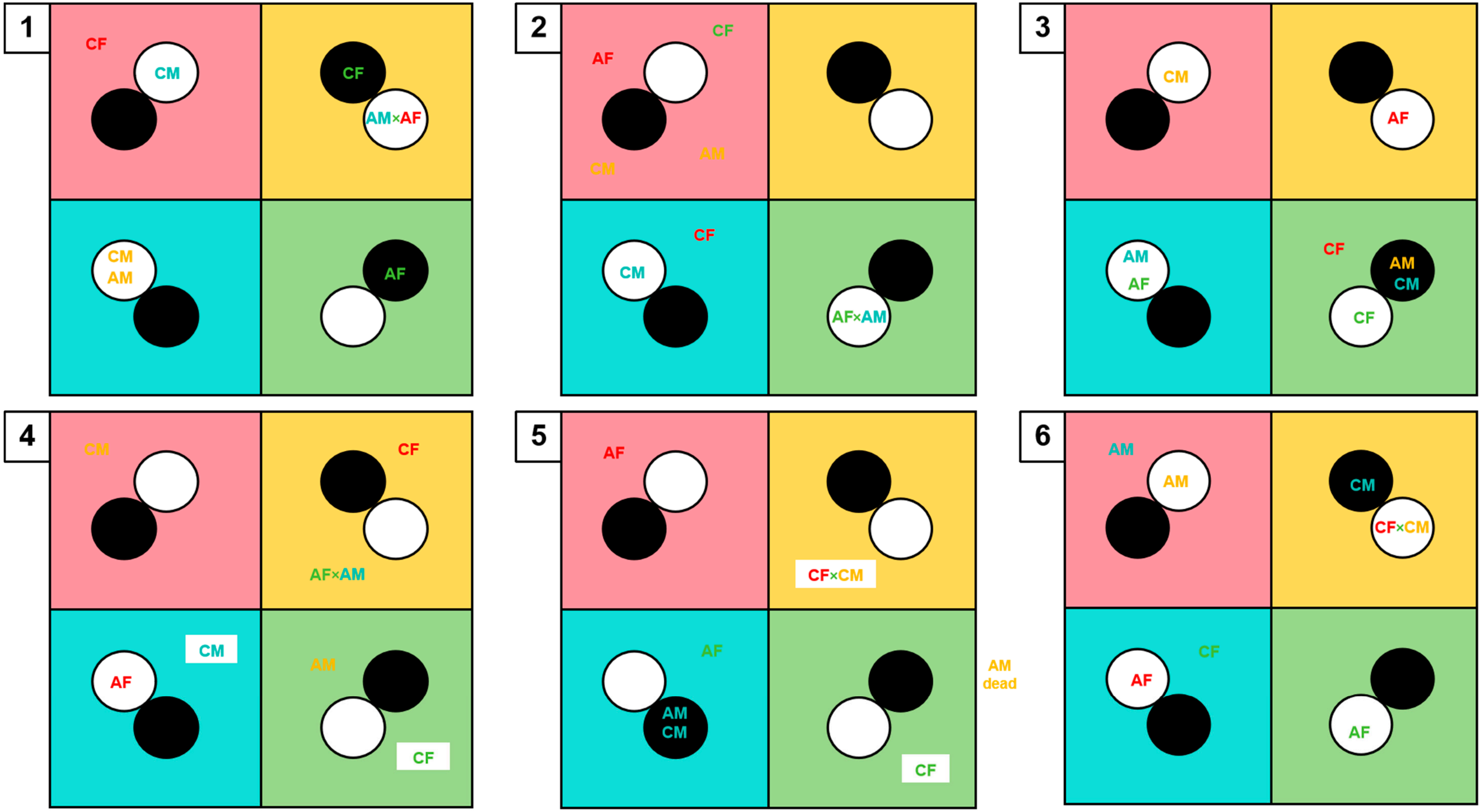

2023.8.3, 8 AM, 26.8℃, 80%RH

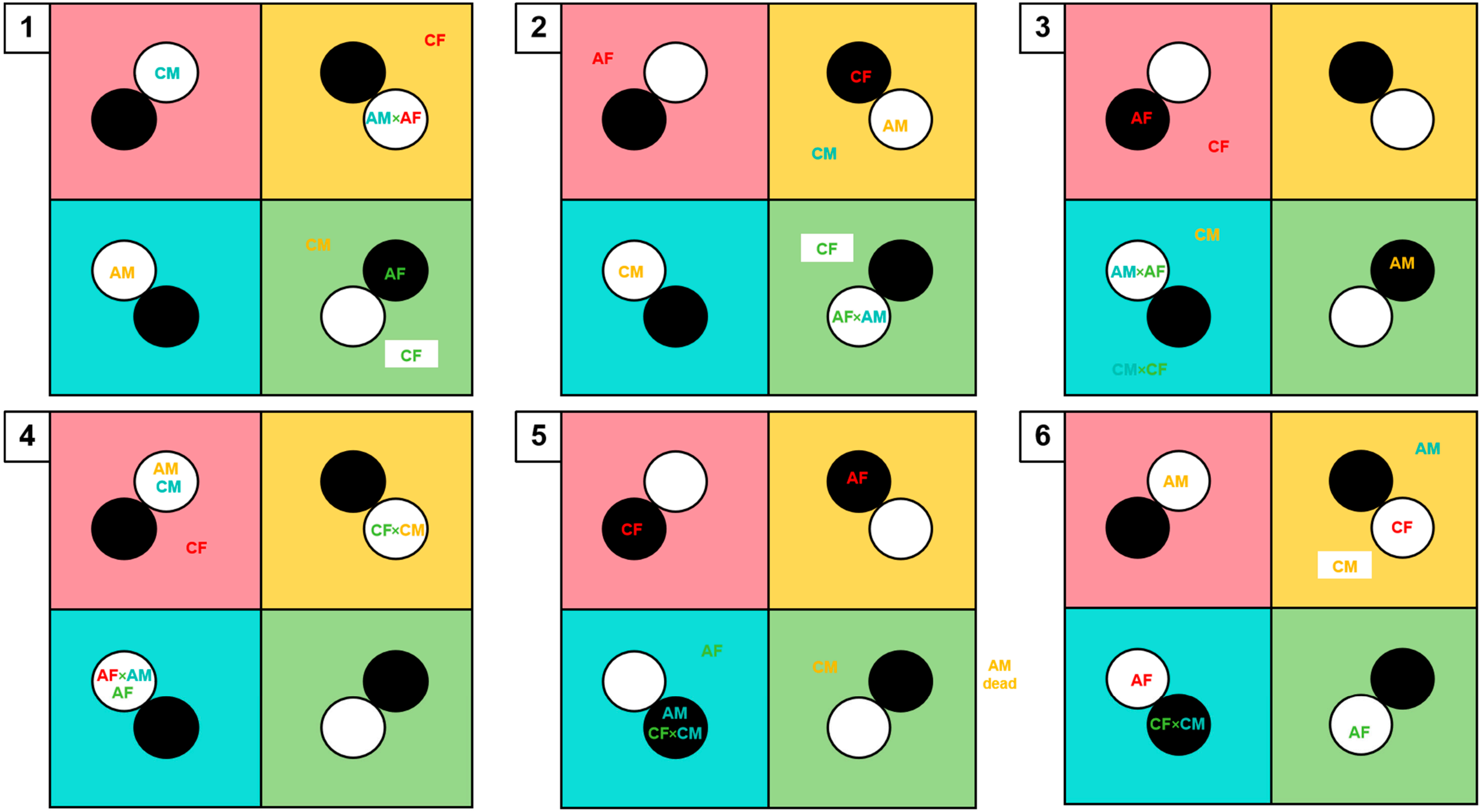

2023.8.3, 10 AM, 29.1℃, 57%RH

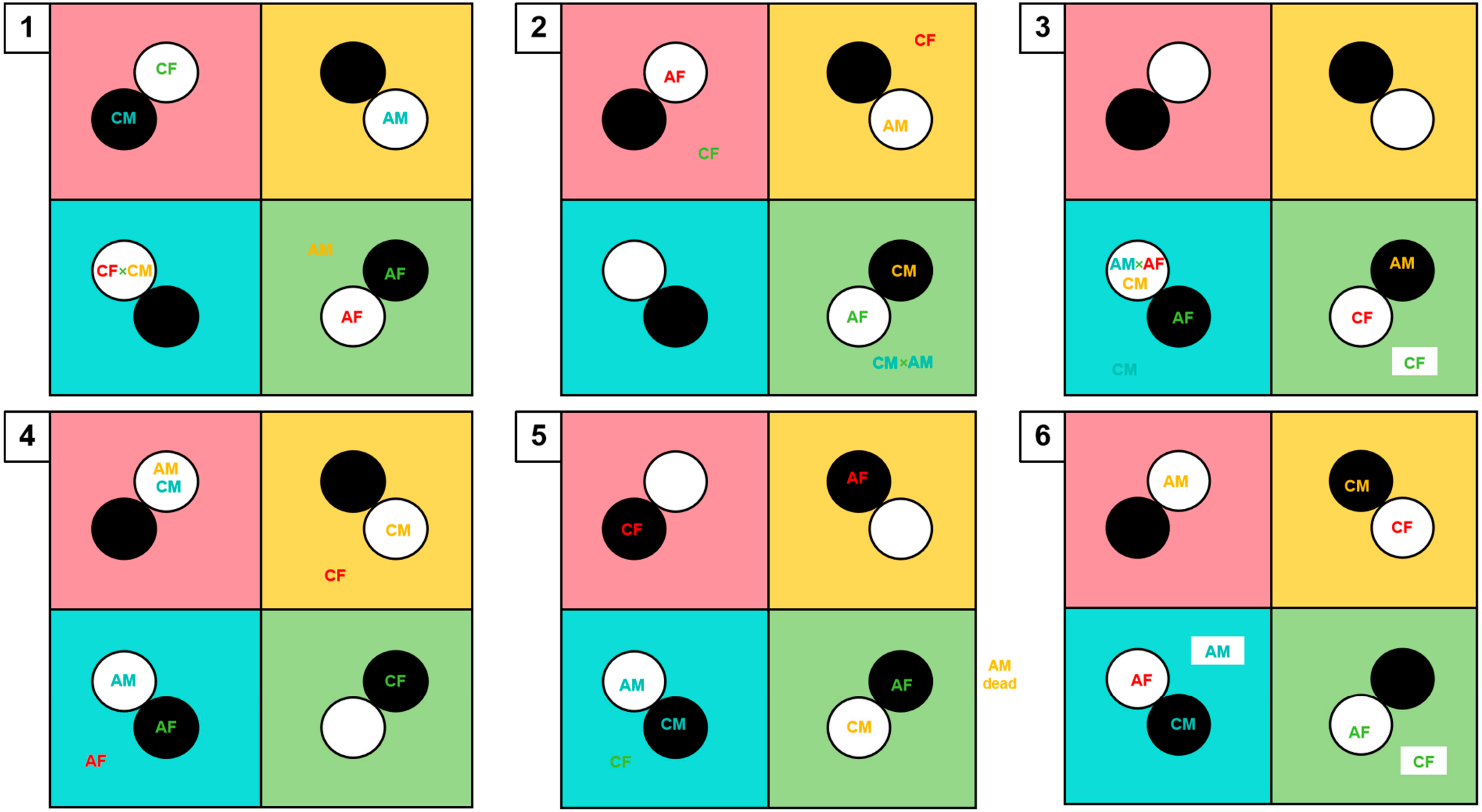

2023.8.3, 12 PM, 30.1℃, 55%RH
